# Long-Term Exposure to Particulate Matter and Mortality: An Update of the WHO Global Air Quality Guidelines Systematic Review and Meta-Analysis

**DOI:** 10.3389/ijph.2024.1607683

**Published:** 2024-09-27

**Authors:** Pablo Orellano, Maria-Iosifina Kasdagli, Román Pérez Velasco, Evangelia Samoli

**Affiliations:** ^1^ Consejo Nacional de Investigaciones Cientificas y Tecnicas (CONICET), Universidad Tecnologica Nacional, Facultad Regional San Nicolas, San Nicolas, Argentina; ^2^ Department of Hygiene, Epidemiology and Medical Statistics, Medical School, National and Kapodistrian University of Athens, Athens, Greece; ^3^ World Health Organization (WHO) Regional Office for Europe, European Centre for Environment and Health, Bonn, Germany

**Keywords:** air pollution, particulate matter, mortality, systematic review, meta-analysis

## Abstract

**Objectives:**

For the development of the 2021 global air quality guidelines, the World Health Organization (WHO) commissioned a series of systematic reviews and meta-analyses to assess the association between exposure to air pollution and all-cause and cause-specific mortality. One of these reviews, which we aim to update, focused on the effects of long-term exposure to PM_2.5_ and PM_10_ on all-cause and cause-specific mortality.

**Methods:**

The protocol for this study was registered in PROSPERO (CRD42023425327). We searched the PubMed and Embase databases for studies published between September 2018 and May 2023. Study-specific effects were pooled using random-effects models.

**Results:**

We included 106 studies in the meta-analysis, 46 studies from the previous review and 60 from this update. All exposure-outcome pairs analysed showed positive and significant associations, except for PM_10_ and cerebrovascular mortality. The certainty of the evidence was rated as high for the majority of exposure-outcome pairs.

**Conclusion:**

We included a large number of new cohorts, and provided new concentration-response functions that will inform WHO advice on the use of this information for air pollution health risk assessments.

## Introduction

According to the World Health Organization (WHO), air pollution is responsible for approximately 7 million attributable deaths annually [1]. Over the past 40 years, WHO has developed a series of publications known as “Air Quality Guidelines” (AQGs) for indoor and ambient air pollution [2], which are widely used as a reference for air quality management by governments and non-government organizations worldwide. The most recent update of these guidelines was published in 2021 and represented a significant advance in the science of air quality and health by analysing and reporting new evidence [3]. To develop these guidelines, the WHO Regional Office for Europe commissioned a series of systematic reviews of the evidence on selected health outcomes associated with long- and short-term exposure to air pollutants [2]. In particular, two of these reviews investigated the effects of long-term exposure to particulate matter (PM_2.5_ and PM_10_) [4] and nitrogen dioxide (NO_2_) and ozone (O_3_) [5] on all-cause and cause-specific mortality. Although these systematic reviews were published recently, there is a rapidly growing body of evidence addressing the same research question, including new cohorts and re-analyses of previously published studies. Accordingly, WHO commissioned new systematic reviews to update the concentration-response functions for the selected pollutants and to revisit the evaluation of the certainty of the evidence on numerical values. The results of these updates, particularly the concentration-response functions, will be used to inform the Update of the Health Risks of Air Pollution in Europe (HRAPIE-2) project, coordinated by the WHO Regional Office for Europe’s European Centre for Environment and Health (ECEH) [6]. The present study is related to one of the previously mentioned reviews, which focused on long-term exposure to particulate matter and mortality [4]. The aim was to update the results of the previous systematic review and meta-analysis that informed the 2021 update of the AQGs.

## Methods

### Protocol and Reporting

The protocol for this systematic review and meta-analysis was registered in the PROSPERO registry (https://www.crd.york.ac.uk/prospero/) under registration number CRD42023425327 prior to the preliminary search for studies. This systematic review and meta-analysis was reported according to the guidelines of the Preferred Reporting Items for Systematic Reviews and Meta-Analyses (PRISMA) standards [7], adapted to the type of information and procedures of observational epidemiological studies. Conformity of the manuscript with the PRISMA guidelines is provided in Supplementary Tables S17A, B, Supplementary File 1.

### Review Question

The research question was formulated according to the format of the Population, Exposure, Comparator, Outcomes and Study Design (PECOS) questions [9]. The content of the question was as follows:

In the general human population (P), what is the effect of long-term exposure to ambient particulate matter (PM_10_, PM_2.5_) (E) *versus* exposure to lower levels of air pollution (difference of 10 μg/m³) (C) on the risk of all-cause and cause-specific mortality (O), as reported in cohort studies (S)?

### Search Strategy

We searched for studies published in PubMed and Embase. The search timeline of the previous systematic review [4] included studies up to September/October 2018. Accordingly, our search started in September 2018 and ended in May 2023. The search strategy included a combination of free text and Medical Subject Headings (MeSH) terms related to exposure, outcome and study design. In addition, reference lists of selected studies and reviews were scanned for relevant studies not identified by the formal search strategy. The search lines for both databases can be found in Supplementary Table S18, Supplementary File 1.

We updated our search in January 2024 to assess the influence of more recent studies published after May 2023. The studies identified in this new search were not included in the meta-analysis, but were listed, their results summarised, and the effect estimates compared with our pooled estimates.

### Eligibility Criteria and Selection Procedure

We included prospective and retrospective cohort study designs, but excluded case-control studies and related designs, panel and cross-sectional studies. Other reasons for excluding studies were occupational or indoor exposure only, qualitative designs, reviews and non-human studies (i.e., *in vivo*, *in vitro*). We considered long-term exposure (of the order of months to years) to ambient PM_2.5_ and PM_10_ from any source, expressed as a concentration unit (μg/m³). Indoor and occupational exposures to these pollutants were excluded from the analysis. This long-term exposure was compared with lower levels of PM_2.5_ and PM_10_ in the same or a control population. The systematic review by Chen and Hoek [4] included case-control designs, but only three of these studies were found, and they were not included in the meta-analysis. Because the current systematic review focuses on association values and the certainty of the evidence around them, we focused on cohort studies.

Outcomes were all-cause and cause-specific mortality, including all causes (A00-Z99), circulatory diseases (I00-I99), ischaemic heart disease (IHD) (I20-I25), cerebrovascular diseases (I60-I69), respiratory diseases (J00-J99), chronic obstructive pulmonary disease (COPD) (J40-J44, J47), acute lower respiratory infections (ALRI) (J12-J18, J20-J22) and lung cancer (C33-C34: malignant neoplasm of the trachea, bronchus or lung only). See Supplementary Table S19, Supplementary File 1, for a description of the inclusion of diseases in categories and subcategories according to the International Classification of Diseases (ICD-10), 10th edition codes. Although all-cause mortality includes accidental deaths, the proportion of deaths caused by accidents, etc., is typically small (<10%) in comparison with the other causes of death [3]. Therefore, as in the previous review, we considered natural causes to be equivalent to all-cause mortality. We also included post-neonatal mortality in children aged 28 days to 12 months, which was analysed separately in the previously mentioned meta-analysis of all-cause mortality. In terms of participants, the study included the general population, of all ages, in all locations, regardless of level of development, income or urbanisation. There were no geographical restrictions, but studies limited to patient cohorts were excluded from the analysis. It is worth noting that the systematic review by Chen and Hoek [4] included infant mortality and patient-only cohorts.

PO and ES independently screened the titles and abstracts of the retrieved studies. In the final step, the full text of potentially eligible studies was assessed by the same reviewers. For exclusions at this stage, the reason was recorded in the forms. In case of disagreement, the two reviewers discussed and reached a consensus on the inclusion of the studies. All studies flagged for inclusion at the full-text assessment stage were included in the systematic review, but for the meta-analysis some studies were excluded if there was total or partial overlap between cohorts, i.e., if two or more studies reported data on the same cohort, typically in large collaborative multicentre studies. To decide how much overlapping data was acceptable to consider the studies as independent, our criterion was that both studies were included if the number of additional years reported in a cohort-specific study was equal to or greater than the number of overlapping years between the two studies, or if the number of participants in the cohorts differed by more than 25%. When overlap was identified, one of the studies was selected and the other was excluded based on the same pair of criteria: 1) the study with the larger sample size, and 2) the study with the largest time period.

### Data Collection

We extracted information on publication details (title, authors, year of publication), study characteristics (study design, location, study population), exposure to the air pollutants of interest and ambient pollution levels (mean and median concentrations), outcome (cause of death), relative risks (RR), hazard ratios (HR) and odds ratios (OR) and 95% confidence intervals (CI) with corresponding exposure increments derived from single-pollutant models. We also collected information on the shape of the concentration-response functions and on two-pollutant models. In some studies, authors reported more than one effect estimate for the same exposure-outcome pair. In this case, we selected the estimate for inclusion in the meta-analysis based on single-effect estimates derived from the model identified by the authors as “main” in the methods section. If we could not identify the main model, we selected the simpler model that included all or as many of the critical confounders as possible, i.e., age, sex, body mass index (BMI), and socioeconomic status (SES). For studies not included in the previous classification, a case-by-case assessment and other considerations, such as the size of the subcohort for the specific model or other relevant *ad hoc* criteria, were taken into account. In addition, some studies may have contributed more than one independent effect estimate for a given exposure-outcome pair, for example, when a study reported results from two or more different cohorts or sites. For this reason, the number of effect estimates may be larger than the number of studies included.

### Risk of Bias Assessment

To assess the risk of bias in individual studies, we used a domain-based risk of bias tool specifically designed for air pollution epidemiology studies. This tool was developed for the 2021 WHO global air quality guidelines. A detailed description of this tool can be found on the WHO website [12] and was also explained in the previous review [4]. Briefly, this tool considers 13 items grouped into 6 domains: confounding, selection bias, exposure assessment, outcome measurement, missing data and selective reporting, and is based on the Risk Of Bias In Non-randomised Studies (ROBINS) instrument [13].

### Data Synthesis

Effect estimates reported in individual studies could be expressed as RRs, HRs, ORs or percentage increase in the risk (Perc.-incr.), but pooled estimates were calculated and reported as RRs. HRs and ORs were considered to be numerically equivalent to RRs, in the latter based on the “rare disease assumption” [14]. Perc.-incr. were converted to RRs using the following equation:

$$RR=\frac{Perc.-incr.}{100}+1$$

The RRs were previously standardised to reflect a risk increase associated with a 10 μg/m^3^ increase in the pollutant of interest, as some studies reported estimates for this increase, while others reported values related to interquartile range or unit differences. Standardisation was performed using this equation:

$${RR}_{\left(standardised\right)}=e^{\frac{\mathrm{Ln}\left({RR}_{\left(original\right)}\right)\times 10}{{Increment}_{\left(original\right)}}}$$

We applied a random-effects model, because we assumed that some heterogeneity between studies was to be expected, given the observational nature of the included study designs. The DerSimonian-Laird estimator was chosen to estimate between-study variability [15], which was chosen in the previous review [4]. We also calculated the I^2^ and 80% prediction intervals, a measure of the observed variance without the effect of sampling error and a measure of how much effects vary across populations, respectively [16]. Prediction intervals [17] were used as a sign of heterogeneity, with the following rule: if 1) the prediction interval includes unity, and 2) the prediction interval is wider than twice the 95% CI, then there may be concerns about heterogeneity. Irrespective of the degree of heterogeneity suspected, an attempt was made to identify possible sources of potential heterogeneity through subgroup analysis and meta-regression. We investigated potential differences between WHO regions by comparing the observed Q values with their expected values, assuming a Chi-Squared distribution, while the influence of air pollution levels on the pooled effect estimates was assessed by meta-regression.

Sensitivity analyses were performed to estimate the degree of influence of the risk of bias on the effect estimates. We excluded the studies with a high risk of bias in each of the six domains and recalculated the effect estimates for studies with a low and moderate risk of bias to see if there were any changes in the direction or significance of the effect estimates.

To assess the potential for publication bias, we calculated Egger’s test and generated funnel plots to look for asymmetries.

In addition to the effect estimates, we also retrieved information on the shape of the concentration-response functions reported in individual studies and developed a narrative description of these results. We also identified studies that analysed two-pollutant models and narratively described these results in comparison with the effects estimated from single-pollutant models.

All statistical analyses were performed using the “meta” package [18] of the R statistical software, version 4.2.2 (https://www.r-project.org/).

### Certainty of the Evidence

To assess the certainty of the evidence, we used a modified version of the Grading of Recommendations, Assessment, Development and Evaluation (GRADE) approach [19], developed by an expert group convened by WHO in the context of 2021 global air quality guidelines. A description of this tool can be found in Supplementary Table S22, Supplementary File 1.

### Additional Details and Deviations From the Protocol

In the case of studies with overlapping data, we kept the same criteria for selecting the study for inclusion in the meta-analysis as in the protocol. However, we refined the definition of what could be considered as overlap. We also developed a criterion for selecting the effect estimate to be included when the same study reported more than one effect estimate. To assess heterogeneity in a given exposure-outcome pair, we also considered the consistency in the direction of effect estimates between studies and the extent to which this potential variability was explained by the subgroup analyses. In addition, we developed a set of criteria to assess the potential presence of publication bias. All these procedures are described in detail in the relevant sections of this manuscript. Finally, according to the protocol, a likelihood-based random-effects model, restricted maximum likelihood (REML), should be used. However, we decided to use the more commonly used DerSimonian-Laird estimator, as this was the statistic used in the previous review.

## Results

### Description of Studies

**FIGURE 1:**
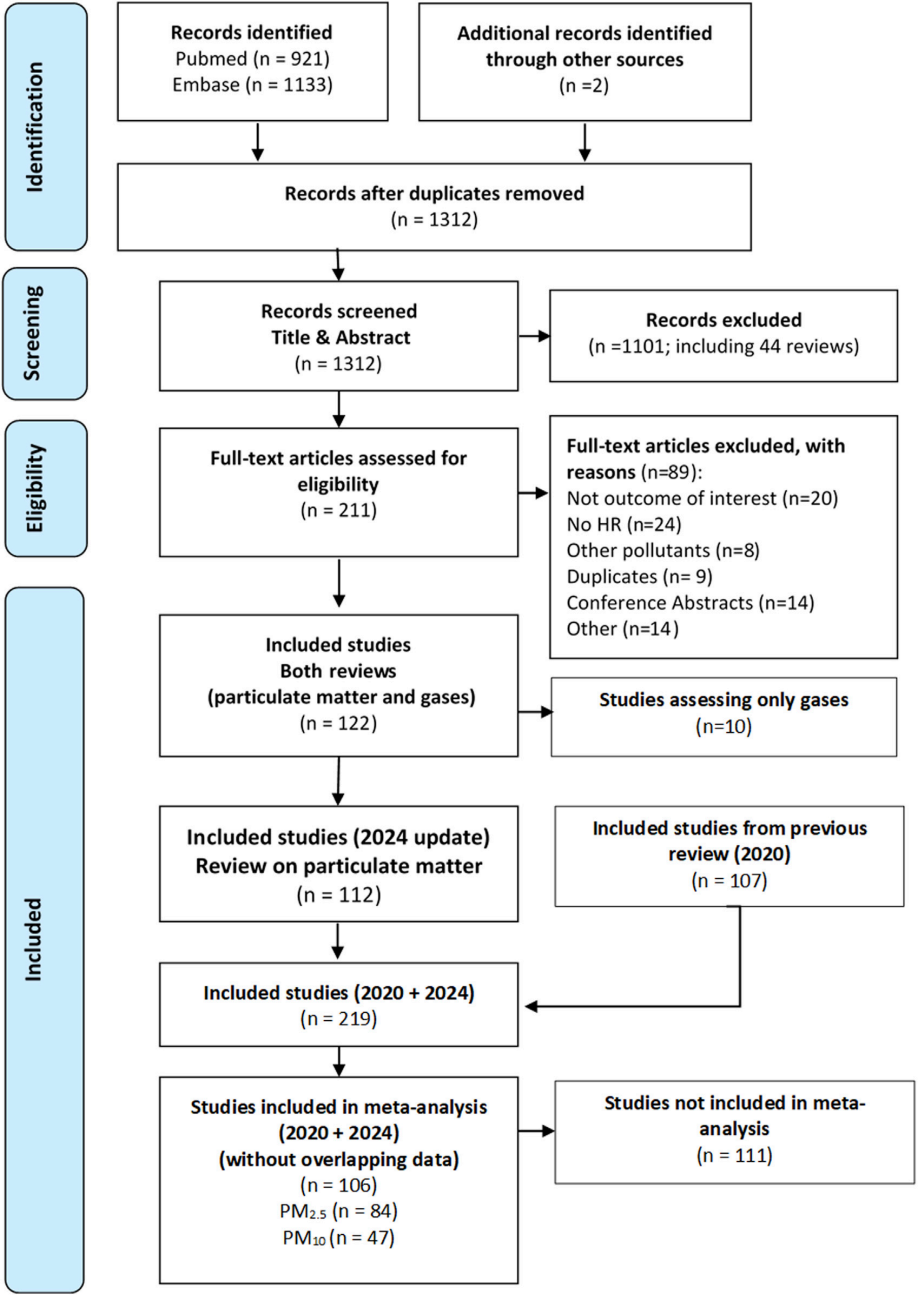
Flowchart of studies selected for inclusion in the systematic review and meta-analysis (Global, 2023–2024).

The PubMed and Embase searches for this review identified 2,054 studies, plus two additional studies identified in the bibliography of relevant reviews. After duplicate records were excluded, 1,312 studies were assessed by title and abstract. A total of 211 full-text articles were assessed for eligibility, 89 were excluded as they did not meet the inclusion criteria, and 122 were finally included for the quantitative meta-analysis of PM_2.5_, PM_10_, NO_2_ and O_3_. For this study, 112 studies from this new search analysing exposure to PM_2.5_ and PM_10_ were initially included. Starting with these 112 studies and adding the 107 studies included in the previous review [4], we applied the process of excluding studies reporting overlapping data. After this process, we finally arrived at a set of 106 studies that were included in the meta-analysis of the effects of PM_2.5_ and PM_10_ on all-cause and cause-specific mortality, including studies selected for the previous review (46 studies) and for this update (60 studies). These 106 studies contributed 379 effect estimates for all exposure-outcome pairs considered, i.e., some studies reported more than one pollutant-outcome pair for analysis. The following descriptions and results in this and subsequent sections refer to both sets of studies, those from this update and those from the previous review combined. The flowchart for this selection process is shown in Figure 1, and the excluded studies, with reasons, are shown in the Supplementary File 2. Supplementary File 3 shows the process of replacing articles with overlapping data on a case-by-case basis.

The studies covered results from several countries and regions, including five WHO regions: The European Region (EUR) (31 studies), the Region of the Americas (AMR) (34 studies), the Western Pacific Region (WPR) (36 studies), the Eastern Mediterranean Region (EMR) (1 study), the South-East Asian Region (SEAR) (2 studies), and two studies covering more than one region. The total number of participants was 948,483,820 when all cohorts were included. The majority of studies focused on adult populations (96 studies), while some studies analysed all age groups (7 studies) and three studies analysed populations under 5 years of age. We have calculated the average (mean) value of the mean/median ambient PM concentration reported in each individual study, generally mean/median values per year or over the whole study period. There were 84 studies analysing exposure to PM_2.5_ with an average of the mean/median concentration of 23.6 μg/m^3^, while 47 studies analysed PM_10_ with an average of the mean/median concentration of 43.7 μg/m^3^. These average values for PM_2.5_ are high, mainly due to the mean concentrations found in China. Information on the new studies included in this review and all studies used in further analyses can be found in Supplementary Files 4, 6.

### Risk of Bias

Of the six domains analysed using the risk of bias tool, only one domain (missing data) was free of studies with a high risk of bias. All other domains had at least one study rated as “high risk of bias” for a specific exposure-outcome pair, although the proportions were very different. The domain with the highest proportion of studies with a high risk of bias was confounding (13%), followed by exposure assessment (2%). A larger proportion of studies were classified as having a moderate risk of bias in the domains of confounding (79%) or exposure assessment (26%). Among the potential confounders not taken into account, the most common was BMI, followed by ethnicity, smoking and diet. Other potential confounders not taken into account were physical activity, marital status, socioeconomic status and year of enrolment. All these results are presented in Supplementary Figure S1, Supplementary File 1. The detailed ratings and justifications for the risk of bias assessments are provided on a case-by-case basis in Supplementary File 5 for new studies and in Supplementary File 6 for all studies included in the meta-analysis. The overall results of the risk of bias assessment by domain are reported in Supplementary Tables S23, Supplementary File 1.

### Meta-Analysis

#### All-Cause mortality

**FIGURE 2:**
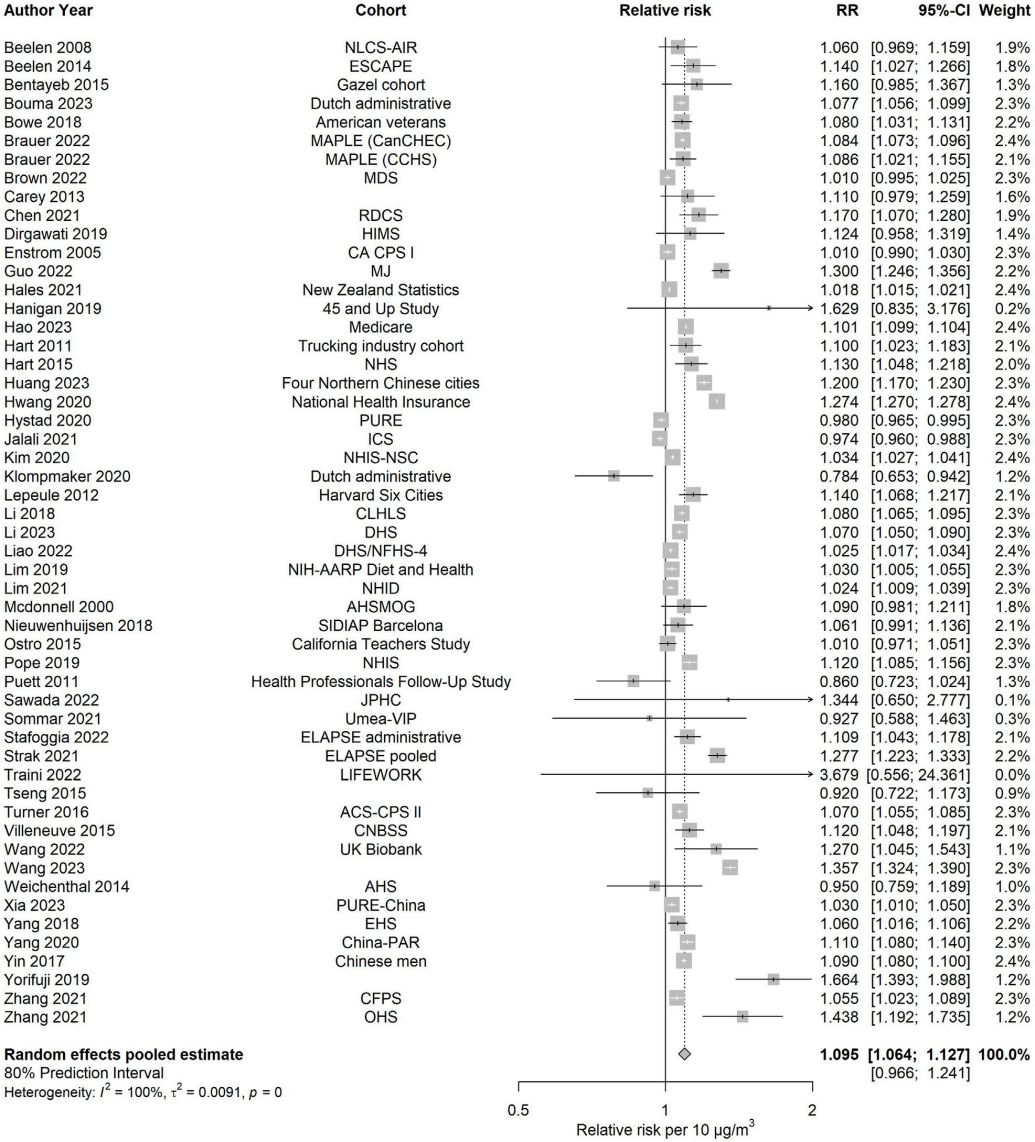
Forest plot examining the association between PM_2.5_ and all-cause mortality (Global, 2023–2024).

All the estimations for all-cause and cause-specific mortality, together with other relevant estimates, are shown in Table 1. The pooled effect estimate for PM_2.5_ and all-cause mortality was based on 53 effect estimates or 52 studies. A 10 μg/m^3^ increase in ambient PM_2.5_ was associated with an increased risk of all-cause mortality (RR: 1.095; 95% CI: 1.064–1.127). The 80% prediction interval included unity (0.966–1.241) and was more than twice the value of the 95% confidence interval. The I^2^ value was high (99.6%), meaning that much of this variability was due to between-study variability rather than sampling error. Figure 2 depicts the forest plot. No evidence of funnel plot asymmetry was observed (Supplementary Figure S16, Supplementary File 1), and the Egger’s test yielded no significant results, suggesting that publication bias is unlikely.

**TABLE 1:** Pooled effect sizes for the exposure-outcome pairs (Global, 2023–2024).

Pollutant	Outcome (mortality)	N	RR (95% CI)	PI	I^2^ (%)	E	Median pollutant level (µg/m^3^) (min.-max.)
PM_2.5_	All-cause	53	1.095 (1.064–1.127)	0.966–1.241	99.6	0.45	16.58 (4.49–72.4)
	Circulatory	42	1.127 (1.102–1.152)	1.046–1.214	95.3	<0.05	14.7 (6.78–59.3)
	IHD	34	1.143 (1.102–1.186)	1.026–1.273	94.1	<0.05	13.8 (4.08–59.3)
	Cerebrovascular	28	1.146 (1.101–1.192)	1.034–1.270	91.6	<0.05	14.2 (6.78–66.3)
	ALRI	12	1.204 (1.095–1.325)	1.005–1.443	81.5	0.12	11.3 (6.78–42.2)
	Lung cancer	26	1.093 (1.053–1.135)	0.999–1.196	84.0	<0.05	13.45 (6.78–58.35)
	Respiratory	28	1.136 (1.079–1.197)	0.990–1.305	89.4	<0.05	13.45 (4.9–58.35)
	COPD	19	1.138 (1.080–1.198)	1.023–1.266	84.2	0.31	13.45 (4.1–43.7)
	Post-neonatal	2	N/A	N/A	N/A	N/A	N/A
PM_10_	All-cause	28	1.081 (1.052–1.110)	0.991–1.179	98.1	<0.05	25.1 (4.49–112.06)
	Circulatory	26	1.080 (1.042–1.120)	0.973–1.200	98.0	0.09	28.8 (4.49–154)
	IHD	16	1.055 (1.019–1.092)	0.985–1.129	87.7	0.24	26.8 (15.9–62.05)
	Cerebrovascular	15	1.049 (0.973–1.131)	0.880–1.250	98.5	0.50	24.2 (4.49–154)
	ALRI	1	N/A	N/A	N/A	N/A	N/A
	Lung cancer	17	1.101 (1.052–1.152)	0.995–1.218	94.2	0.06	27.9 (4.49–112.06)
	Respiratory	21	1.122 (1.076–1.169)	1.019–1.235	92.3	0.05	29 (4.49–144.3)
	COPD	7	1.215 (1.027–1.438)	0.886–1.667	82.9	N/A	23.75 (4.49–144.3)
	Post-neonatal	1	N/A	N/A	N/A	N/A	N/A

N, number of estimates; RR, pooled relative risks; 95% CI, 95% confidence interval; PI, 80% prediction interval; E, Egger’s test (*p*-value); IHD, ischaemic heart disease; ALRI, acute lower respiratory infection; COPD, chronic obstructive pulmonary disease; N/A, not applicable (<10 studies for the Egger’s test or <3 studies for pooled relative risks).

The pooled effect estimate for PM_10_ and all-cause mortality was positive and significant, based on 28 effect estimates (RR: 1.081; 95% CI: 1.052–1.110). The 80% prediction interval (0.992–1.180) included unity and doubled the 95% confidence interval, while the I^2^ value was high (98.0%) (see Supplementary Figure S2, Supplementary File 1, for the forest plot). For this exposure-outcome pair, the asymmetry of the funnel plot is more pronounced, with a significant Egger’s test value, indicating a potential for publication bias (Supplementary Figure S17, Supplementary File 1).

#### Cause-Specific Mortality

For PM_2.5_ and cause-specific mortality, all associations were positive and significant, with relative risks ranging from 1.093 (lung cancer mortality) to 1.146 (cerebrovascular mortality). The forest plots for these exposure-outcome pairs are shown in Supplementary Figures S3–S9, Supplementary File 1. The 80% prediction interval included unity and was more than twice the 95% CI for lung cancer and respiratory mortality. An asymmetry in the funnel plot was observed in five exposure-outcome pairs according to the Egger’s test, which may indicate potential publication bias. The funnel plots are shown in Supplementary Figures S18–S24, Supplementary File 1.

The associations between PM_10_ and cause-specific mortality were all significant except for cerebrovascular mortality (see Supplementary Figures S10–S15, Supplementary File 1, for the forest plots). Except for respiratory mortality, all the 80% prediction intervals included unity, but in some cases the 80% prediction interval was narrower than the 95% CI, while all I^2^ values were greater than 80%. No evidence of funnel plot asymmetry was observed (see Supplementary Figures S25–S30, Supplementary File 1, for the funnel plots).

The associations between PM_2.5_ and PM_10_ and post-neonatal mortality were not included in the meta-analysis of all-cause mortality and were not meta-analysed separately because only two studies reported these estimates [8, 10]. However, these were positive and significant. There were no studies reporting on the association between PM_10_ and acute lower respiratory infections (ALRI).

### The Shape of the Concentration-Response Functions

#### General Description

Of the included studies, 33 assessed the shape of the concentration-response functions, including 25 for PM_2.5_, two for PM_10_ and six for both. A table with descriptions for each study can be found in the Supplementary File 7.

#### PM_2.5_ – All-Cause Mortality

Six studies from Australia, Denmark, Netherlands (Kingdom of the), Japan and Taiwan, China found no evidence of non-linearity [11, 20–24], while others reported non-linear associations. A supralinear concentration-response function was reported in the ELAPSE study from the European Region, in the MAPLE-CanCHEC study in Canada [25–27] and in one study from China [28], with a steeper slope at concentrations below 60 μg/m³. In contrast, the Medicare study from the United States [29] reported sublinear curves with steeper slopes in the middle ranges. The aforementioned studies from the European Region (ELAPSE) and Canada (MAPLE-CanCHEC) found no evidence for a threshold below which no effects are observed [25, 27, 30]. Conversely, the study from the United States showed that lower relative risks were estimated when PM_2.5_ concentrations were below approximately 10 μg/m^3^ [29].

#### PM_2.5_ – Cause-Specific Mortality

The associations between PM_2.5_ and cause-specific mortality were generally linear or supralinear [21, 22, 27, 31–34] for the majority of studies. An exponential shape was found for cardiovascular mortality, with steeper slopes at higher PM_2.5_ levels, e.g., above 60 or 80 μg/m^3^ [35, 36]. Non-linearity was suggested for ALRI mortality [22], lung cancer and IHD mortality [37]. However, there was no evidence for a threshold below which PM_2.5_ concentrations could be considered safe [27, 30, 34, 38].

#### PM_10_ – All-Cause and Cause-Specific Mortality

The number of studies analysing the shape of the concentration-response function is much smaller for PM_10_. The studies reported linear, supralinear and sublinear curves [20, 24, 32, 34, 39, 40]. One study investigated the existence of potential thresholds [34], but failed to find a PM_10_ level below which the risk of death is negligible.

### Subgroup Analysis

Comparisons between WHO regions are shown in Tables 2, 3, and the associated forest plots are shown in Supplementary Figures S31–S45, Supplementary File 1. For PM_2.5_, the only statistical difference between regions was for lung cancer, with higher relative risks reported in the European Region and non-significant relative risks in the Region of the Americas. The same difference was observed for PM_10_, with higher relative risks for the European Region, but non-significant relative risks for the Region of the Americas and the Western Pacific Region. Although the differences are not significant, when analysing exposure to PM_2.5_, the relative risks appear to be consistently lower in the European Region for circulatory and IHD mortality, but higher for cerebrovascular, respiratory and COPD mortality. On the contrary, for PM_10_ the higher relative risks come from the Western Pacific Region, with a value as high as 1.285 for COPD mortality, but generally with wide confidence intervals.

**TABLE 2:** Pooled effect sizes for PM_2.5_ and all-cause and cause-specific mortality. Subgroup analysis by World Health Organization region (Global, 2023–2024).

Outcome (mortality)	WHO region	N	RR (95% CI)	I^2^ (%)	80% PI	*p*-value
All-cause	EUR	12	1.103 (1.038–1.173)	84.4	0.905–1.346	0.19
	AMR	17	1.075 (1.055–1.096)	89.9	1.005–1.151	
	WPR	19	1.139 (1.068–1.216)	99.9	0.855–1.518	
Circulatory	EUR	10	1.091 (1.008–1.179)	72.6	0.875–1.360	0.51
	AMR	15	1.147 (1.107–1.188)	86.9	1.022–1.287	
	WPR	15	1.141 (1.102–1.181)	97.3	1.003–1.297	
IHD	EUR	7	1.113 (1.042–1.188)	19.0	0.977–1.268	0.47
	AMR	17	1.166 (1.126–1.207)	68.4	1.058–1.284	
	WPR	9	1.155 (1.070–1.247)	96.0	0.898–1.485	
Cerebrovascular	EUR	7	1.156 (1.037–1.290)	54.7	0.873–1.532	0.60
	AMR	9	1.183 (1.103–1.269)	65.5	0.976–1.435	
	WPR	11	1.128 (1.062–1.197)	96.2	0.932–1.365	
ALRI	AMR	6	1.245 (1.093–1.419)	87.2	0.835–1.858	0.87
	WPR	5	1.220 (0.996–1.494)	78.0	0.638–2.333	
Lung cancer	EUR	6	1.248 (1.179–1.321)	0.0	1.151–1.352	<0.01
	AMR	12	1.022 (0.984–1.061)	49.5	0.932–1.120	
	WPR	8	1.118 (1.037–1.205)	90.9	0.898–1.391	
Respiratory	EUR	8	1.213 (1.025–1.435)	81.7	0.735–2.000	0.53
	AMR	12	1.100 (1.034–1.170)	74.5	0.920–1.316	
	WPR	8	1.094 (1.007–1.188)	88.6	0.854–1.402	
COPD	EUR	3	1.217 (1.077–1.375)	0.0	0.552–2.684	0.09
	AMR	9	1.071 (1.019–1.126)	52.1	0.950–1.207	
	WPR	7	1.182 (1.023–1.364)	88.0	0.768–1.817	

N, number of estimates; RR, pooled relative risks; 95% CI, 95% confidence interval; I^2^, test for heterogeneity; PI, 80% prediction interval; *p*-value, significance of test for difference between subgroups (interaction); IHD, ischaemic heart disease; ALRI, acute lower respiratory infection; COPD, chronic obstructive pulmonary disease; WHO, World Health Organization; EUR, European Region; AMR, Region of the Americas; WPR, Western Pacific Region.

**TABLE 3:** Pooled effect sizes for PM_10_ and all-cause and cause-specific mortality. Subgroup analysis by World Health Organization region (Global, 2023–2024).

Outcome (mortality)	WHO region	N	RR (95% CI)	I^2^ (%)	80% PI	*p*-value
All-cause	EUR	16	1.086 (1.058–1.115)	90.3	0.992–1.190	0.17
	AMR	6	1.033 (0.986–1.082)	79.1	0.886–1.204	
	WPR	6	1.092 (1.012–1.180)	99.5	0.823–1.450	
Circulatory	EUR	13	1.041 (1.014–1.069)	75.2	0.971–1.116	0.15
	WPR	11	1.102 (1.025–1.184)	99.2	0.844–1.439	
IHD	EUR	7	1.013 (0.975–1.052)	62.6	0.922–1.112	0.45
	AMR	5	1.056 (0.988–1.128)	32.8	0.888–1.256	
	WPR	4	1.093 (0.909–1.314)	96.8	0.467–2.557	
Cerebrovascular	EUR	7	0.991 (0.981–1.001)	0.0	0.978–1.004	0.49
	WPR	6	1.066 (0.864–1.315)	99.4	0.495–2.297	
Lung cancer	EUR	8	1.227 (1.090–1.381)	94.9	0.853–1.764	0.03
	AMR	3	1.065 (0.877–1.293)	76.8	0.110–10.309	
	WPR	6	1.038 (0.999–1.079)	83.8	0.927–1.163	
Respiratory	EUR	11	1.110 (1.007–1.225)	91.7	0.816–1.510	0.36
	AMR	3	1.065 (1.006–1.128)	0.0	0.737–1.540	
	WPR	7	1.128 (1.067–1.191)	94.3	0.959–1.326	
COPD	EUR	3	1.228 (1.057–1.428)	0.0	0.463–3.260	0.78
	WPR	3	1.285 (0.971–1.700)	92.8	0.038–43.703	

N, number of estimates; RR, pooled relative risks; 95% CI, 95% confidence interval; I^2^, test for heterogeneity; PI, 80% prediction interval; *p*-value, significance of test for difference between subgroups (interaction); IHD, ischaemic heart disease; ALRI, acute lower respiratory infection; COPD, chronic obstructive pulmonary disease; WHO, World Health Organization; EUR, European Region; AMR, Region of the Americas; WPR, Western Pacific Region.

PM levels did not explain the heterogeneity in the associations between PM_2.5_ and all-cause and cause-specific mortality. On the contrary, the associations with circulatory and cerebrovascular mortality were modified by the effect of ambient PM_10_ levels, resulting in higher relative risks for increases in these concentrations, as shown in Supplementary Table S1, Supplementary File 1. We also compared smaller and larger studies, but found no significant differences, except for PM_2.5_ and cerebrovascular mortality, with higher relative risks in smaller studies (Supplementary Tables S20, S21, Supplementary File 1).

### Sensitivity Analysis

Excluding studies with different types of bias led to changes in both directions (Tables 4, 5), i.e., higher and lower relative risks compared to the original value, but these changes were not important in terms of significance. However, there was one exception, the association between PM_10_ and COPD, where the exclusion of two studies with high risk of confounding bias resulted in a non-significant but positive association.

**TABLE 4:** Sensitivity analysis excluding studies with high risk of bias in specific domains (PM_2.5_) (Global, 2023–2024).

Outcome	RoB domain	N	RR (95% CI)	80% PI
All-cause	Original RR	53	1.095 (1.064–1.127)	0.966–1.241
	Confounding	46	1.100 (1.080–1.121)	1.027–1.179
	Exposure	51	1.094 (1.062–1.127)	0.964–1.241
	Outcome	51	1.096 (1.064–1.129)	0.965–1.244
	Selection	53	1.095 (1.064–1.127)	0.966–1.241
	Selective rep.	52	1.095 (1.064–1.127)	0.966–1.241
Circulatory	Original RR	42	1.127 (1.102–1.152)	1.046–1.214
	Confounding	37	1.137 (1.107–1.168)	1.046–1.237
	Exposure	41	1.124 (1.099–1.149)	1.043–1.211
	Outcome	41	1.132 (1.107–1.159)	1.051–1.221
IHD	Original RR	34	1.143 (1.102–1.186)	1.026–1.273
	Confounding	30	1.144 (1.093–1.198)	1.007–1.301
Cerebrovascular	Original RR	28	1.146 (1.101–1.192)	1.034–1.270
	Confounding	26	1.161 (1.112–1.213)	1.042–1.295
	Selection	28	1.146 (1.101–1.192)	1.034–1.270
	Exposure	28	1.146 (1.101–1.192)	1.034–1.270
ALRI	Original RR	12	1.204 (1.095–1.325)	1.005–1.443
	Confounding	11	1.233 (1.096–1.388)	0.991–1.534
Lung cancer	Original RR	26	1.093 (1.053–1.135)	0.999–1.196
	Confounding	25	1.106 (1.058–1.155)	0.995–1.228
	Selection	26	1.093 (1.053–1.135)	0.999–1.196
	Exposure	26	1.093 (1.053–1.135)	0.999–1.196
	Selective rep.	25	1.092 (1.052–1.134)	0.998–1.195
Respiratory	Original RR	28	1.136 (1.079–1.197)	0.990–1.305
	Confounding	28	1.136 (1.079–1.197)	0.990–1.305
	Exposure	27	1.143 (1.085–1.205)	0.995–1.313
	Selection	28	1.136 (1.079–1.197)	0.990–1.305
	Selective rep.	27	1.134 (1.076–1.195)	0.987–1.302
COPD	Original RR	19	1.138 (1.080–1.198)	1.023–1.266
	Confounding	18	1.157 (1.090–1.228)	1.027–1.303
	Selection	19	1.138 (1.080–1.198)	1.023–1.266

N, number of estimates; RR, pooled relative risks; 95% CI, 95% confidence interval; PI, 80% prediction interval; IHD, ischaemic heart disease; ALRI, acute lower respiratory infection; COPD, chronic obstructive pulmonary disease; Confounding, confounding bias; Selection, selection bias; Exposure, exposure bias; Outcome, outcome bias; Selective rep., selective reporting bias; Original RR, pooled RR calculated without exclusion of studies.

**TABLE 5:** Sensitivity analysis excluding studies with high risk of bias in specific domains (PM_10_) (Global, 2023–2024).

Outcome	RoB domain	N	RR (95% CI)	80% PI
All-cause	Original RR	28	1.081 (1.052–1.110)	0.991–1.179
	Confounding	25	1.078 (1.047–1.109)	0.987–1.177
	Selection	27	1.080 (1.050–1.110)	0.990–1.178
	Exposure	27	1.078 (1.049–1.108)	0.988–1.176
Circulatory	Original RR	26	1.080 (1.042–1.120)	0.973–1.200
	Confounding	21	1.099 (1.051–1.149)	0.973–1.241
	Selection	25	1.079 (1.040–1.120)	0.972–1.199
	Exposure	25	1.057 (1.031–1.084)	0.993–1.126
IHD	Original RR	16	1.055 (1.019–1.092)	0.985–1.129
	Confounding	12	1.054 (1.002–1.110)	0.952–1.168
Cerebrovascular	Original RR	15	1.049 (0.973–1.131)	0.880–1.250
	Confounding	11	1.092 (0.942–1.266)	0.778–1.531
	Selection	14	1.048 (0.970–1.132)	0.878–1.250
	Exposure	14	1.029 (0.985–1.075)	0.952–1.113
Lung cancer	Original RR	17	1.101 (1.052–1.152)	0.995–1.218
	Confounding	13	1.102 (1.036–1.172)	0.964–1.259
	Selection	16	1.099 (1.050–1.151)	0.993–1.217
	Exposure	16	1.096 (1.047–1.146)	0.991–1.211
Respiratory	Original RR	21	1.122 (1.076–1.169)	1.019–1.235
	Confounding	18	1.128 (1.076–1.183)	1.015–1.254
	Selection	20	1.121 (1.075–1.169)	1.017–1.235
	Exposure	20	1.122 (1.077–1.170)	1.019–1.237
COPD	Original RR	7	1.215 (1.027–1.438)	0.886–1.667
	Confounding	5	1.218 (0.996–1.491)	0.827–1.795
	Selection	6	1.233 (1.035–1.469)	0.884–1.720

N, number of estimates; RR, pooled relative risks; 95% CI, 95% confidence interval; PI, 80% prediction interval; IHD, ischaemic heart disease; ALRI, acute lower respiratory infection; COPD, chronic obstructive pulmonary disease; Confounding, confounding bias; Selection, selection bias; Exposure, exposure bias; Outcome, outcome bias; Selective rep., selective reporting bias; Original RR, pooled RR calculated without exclusion of studies.

### Two Pollutant Models

Of the included studies, 21 reported the effect of a second pollutant. In general, the inclusion of gases (NO_2_, NO_x_, O_3_, O_x_, SO_2_) or black carbon in the models did not change the associations between particulate matter and mortality [11, 29]. In a few cases the adjustment attenuated the effect, although still leading to significant associations [25, 26, 30, 41]. In contrast, a DUELS study found an increase in the association between PM_2.5_ and non-accidental mortality after adjustment for NO_2_ [42]. However, two studies found that the association with respiratory mortality was not significant after adjustment for NO_2_ [27, 43]. The results from two pollutant models can be found in the Supplementary File 7 on a case-by-case basis.

### Assessment of the Certainty of Evidence

A summary of the certainty of the evidence ratings for each exposure-outcome pair is given in Supplementary Table S16, Supplementary File 1. Both associations of all-cause mortality with PM_2.5_ and PM_10_ were rated as high certainty of the evidence. Of all the pairs analysed for cause-specific mortality, three combinations of PM_2.5_ or PM_10_ were rated as having moderate certainty of evidence, while all the other exposure-outcome pairs (12) were rated as “high”. A more detailed description of each domain is given in Supplementary Table S22, Supplementary File 1, while the full certainty of the evidence rating for each exposure-outcome pair is shown in Table 6 for PM_2.5_ and all-cause mortality and in Supplementary Tables S2–S15, Supplementary File 1, for the other exposure-outcome pairs. It should be noted that the associations between PM_10_ and ALRI and both associations with post-neonatal mortality were not assessed due to the small number of studies. The results of the certainty of evidence assessment by domain are presented in Supplementary Table S24, Supplementary File 1.

**TABLE 6:** Certainty of the evidence for PM_2.5_ and all-cause mortality (Global, 2023–2024).

Domain	Judgement	Downgrade/upgrade and conclusion
Starting point assessment		Moderate certainty of evidence (3/4)
Limitations in studies	Excluding studies with high risk of bias had little effect on pooled effects	No downgrading (0)
Indirectness	All studies addressed the PECOS question directly	No downgrading (0)
Inconsistency	Heterogeneity was suspected based on the results of the 80% prediction interval. However, the studies showed consistent positive associations	No downgrading (0)
Imprecision	Pooled effects calculated using more than 940,000 person-years	No downgrading (0)
Publication bias	The funnel plot showed no asymmetry	No downgrading (0)
Large effect size	The pooled RR is not large enough to rule out the possibility of unmeasured confounding by other factors	No upgrading (0)
All plausible confounding biases RR to zero	Several potential confounders that would shift the RR in either both directions	No upgrading (0)
Dose-response gradient	Positive and significant association found in the main analysis	Upgrade one level (+1)
Conclusion		High certainty of evidence (4/4)

PECOS, population, exposure, comparator, outcome and study; RR, relative risk.

### Search Update

In January 2024, 201 new records were retrieved. Of these, 30 were included by title/abstract and finally five were included for a narrative description of the results and for comparison with our meta-analytic estimates. Some studies were excluded because they reported on cohorts already included in our meta-analysis, many studies considered particulate matter only to adjust for other risk factors in the models, and there were other reasons for exclusion, which can be seen in the Supplementary File 2. Of the five new studies identified, one study analysed data from the “Scottish Longitudinal Study (SLS),” which included people aged 17 and over in 2002 [44], and found estimates that were higher than our pooled values. For example, for PM_2.5_ and all-cause or respiratory mortality, the hazard ratio was 1.035 (95% CI: 1.023–1.047) and 1.062 (95% CI: 1.028–1.096), respectively, per 1 μg/m^3^ increase in PM_2.5_. Another study from northern Europe [45] also reported higher estimates for all-cause mortality with exposure to PM_2.5_ in 1990, with a hazard ratio of 1.40 (95% CI: 1.04–1.87) per 5 μg/m^3^ increase. Three studies reported data from China with slightly different results. While one study found higher values for PM_2.5_ and all-cause (HR: 1.25; 95% CI: 1.04–1.50) and cardiovascular (HR: 1.38; 95% CI: 1.02–1.86) mortality [46], another study based on data from the China-HEART cohort [47] reported lower values for PM_2.5_ and all-cause (HR: 1.022; 95% CI: 1.014–1.030) and cause-specific endpoints, although the estimates were positive and significant in all cases. Specifically for lung cancer mortality, another study from southern China [48] reported estimates that were higher than our pooled estimates, for example, the hazard ratio between PM_2.5_ and lung cancer was 1.042 (95% CI: 1.033–1.052) per 1 μg/m^3^ increase. Taken together, the new studies strengthen the evidence for an association between PM and mortality, as the information provided is in the same direction as our estimates.

## Discussion

This updated systematic review and meta-analysis greatly expanded the evidence base of the previous related review that informed the 2021 WHO global air quality guidelines. We have found evidence of significant and positive associations between long-term exposure to PM_2.5_ and PM_10_ and all-cause and cause-specific mortality, with the exception of exposure to PM_10_ and cerebrovascular mortality, for which the association was positive but not significant. The associations with post-neonatal mortality were not meta-analysed due to small sample sizes, but the associations were significant in individual studies. The other association for which no estimates were calculated due to small sample size was PM_10_ and acute lower respiratory infection (ALRI). Compared with the previous review, our concentration-response functions were generally higher, while for PM_10_ and mortality from circulatory disease and COPD we found significant associations, contrary to previous results. In this sense, the trend of the new evidence in the published literature seems to show a consistent increase in the relative risks between PM exposure and health.

We reported a number of exposure-outcome pairs with wide prediction intervals, indicating heterogeneous results around the estimates, consistent with the previous review. The decision to classify a pooled effect estimate as heterogeneous has negative implications for the certainty of the evidence assessment, so caution was needed. For some associations with wide prediction intervals, we qualitatively analysed the forest plots and relative risks in individual studies and decided that this heterogeneity was not relevant enough, at least to downgrade the evidence. This was the case, for example, for PM_10_ and all-cause mortality. The rationale was that a degree of heterogeneity is to be expected in observational studies [49, 50], and for this particular association the relative risks across studies were consistently positive and significant, increasing certainty in the association. It is worth noting that heterogeneity, while a known limitation, can actually be seen as an asset: a persistently positive value is a way of showing that the effect is consistent across different contexts, an indication of external validity [51]. Beyond the relevance given to heterogeneity, we sought to explain its possible source by performing subgroup analyses by WHO region and by ambient pollutant levels. In the first analysis, we detected differences only for lung cancer mortality and PM_2.5_ or PM_10_, with higher effect estimates for Europe and lower for the Americas and the Western Pacific Region. This difference between regions may be due, among other things, to differences in the source of air pollution. It is worth noting that the penetration of diesel car engines in Europe has been much greater than in the United States, an event referred to as the “European diesel car boom” [52]. However, the diesel fleet in the United States is much larger, so this difference may be better explained by differences in PM composition and other factors. In the second analysis, the meta-regression showed differences only for PM_10_ and circulatory and cerebrovascular mortality, with a positive regression slope, i.e., the effect estimates were higher for higher levels of ambient pollutants. This implies that differences in ambient concentrations of PM_10_ can partly explain the heterogeneity in these pollutant-outcome pairs.

According to the literature, publication bias is a problematic issue. Indeed, some of the exposure-outcome pairs did showed asymmetry in the funnel plots, as assessed by Egger’s test and visual inspection. However, this asymmetry may be strongly influenced by heterogeneity, and the funnel plot may be inappropriate to indicate publication bias [53]. In the light of the above considerations, an additional criterion was used in the analysis of the certainty of the evidence. This involved examining the discrepancy between larger and smaller studies. When comparing the two groups, if no statistical differences were observed, the possibility of publication bias was considered insignificant, as the likelihood of publication in larger studies is unlikely to be influenced by the study results, as explained in the methodology. These differences were found only for PM_2.5_ and circulatory, cerebrovascular and COPD mortality, but for this last exposure-outcome pair the funnel plot was not flagged for asymmetry. Publication bias was then only considered relevant for the first two pairs.

In terms of risk of bias assessment, the proportion of studies with a high risk of bias in each domain was generally low, with the confounding domain being the most prominent. The problem in this domain was mainly related to the lack of adjustment for two of the critical confounders: body mass index and smoking. It should be noted that the measurement of these two variables requires the use of individual questionnaires developed for the purpose of the research, whereas most administrative cohorts do not have access to this information. It is interesting to note that some studies adjusted for these two variables using an indirect method [54], which has proven to be a good alternative when individual data are not available [55]. For studies using this approach, we applied a “moderate” risk of bias rating. It is worth pointing out that the sensitivity analysis based on the risk of bias domains is not affected by the difference between moderate and low rates, as these two categories are considered equivalent for this analysis. In any case, the sensitivity analysis based on the exclusion of studies at high risk of bias did not affect the results, which supports the strength of the evidence for an association.

The shape of the concentration-response functions was investigated in a number of studies. In line with the previous systematic review and the suggestions from the 2021 WHO global air quality guidelines, it was generally assumed to be linear or near-linear, with no evidence of thresholds. It is worth noting that some studies found supralinear functions, with the steepest part of the curve at low exposures, reinforcing the idea that tackling air pollution could lead to health improvements even in areas with low air pollution levels [26].

As a complementary analysis, we reviewed the studies to look for differences between single and two-pollutant models in terms of effect estimates. With some exceptions, the effect estimates were generally attenuated when adjusting for a second pollutant in regression models, although these associations were still positive and significant.

Compared to Chen and Hoek [4], this update found higher estimates for the association between PM_2.5_ and PM_10_ and all-cause and cause-specific mortality, but with wider 95% confidence intervals. The only exceptions were for PM_2.5_ and lung cancer, and for PM_10_ and IHD. In addition, the certainty of the evidence remains high for the majority of the associations. The relative risk values and certainty of the evidence ratings comparing the current and previous review are shown in Table 7. Similarly, the estimates considered in the “Health Risks of Air Pollution in Europe” (HRAPIE) project [56], an international project coordinated by the WHO Regional Office for Europe, were also lower than those reported in this current review, but were based on much less evidence. The greater heterogeneity observed in this review compared with the previous one is probably due to the inclusion of a large number of studies, particularly from the Western Pacific Region. In particular, in the previous review, the number of studies from the Americas was more than double the number of studies from the other regions, whereas in this review these numbers were balanced. In fact, this higher heterogeneity around relative risks in the Western Pacific Region can be seen in the forest plots of the subgroup analysis by WHO region. The contribution of individual studies with wide confidence intervals to the estimated heterogeneity is not very relevant, because these studies have a low weight in the meta-analysis and their exclusion does not affect the pooled relative risk values or the heterogeneity measures.

**TABLE 7:** Comparison of the relative risks and certainty of the evidence between the previous review [4] and this update, for selected outcomes (Global, 2018 and 2023–2024).

Pollutant	Outcome (mortality)	Current systematic review			Chen and Hoek [4]		
		N	RR (95% CI)	Certainty of the evidence	N	RR (95% CI)	Certainty of the evidence
PM_2.5_	All-cause	53	1.095 (1.064–1.127)	High	25	1.08 (1.06, 1.09)	High
	Circulatory	42	1.127 (1.102–1.152)	Mod.	21	1.11 (1.09, 1.14)	High
	IHD	34	1.143 (1.102–1.186)	High	22	1.16 (1.10, 1.21)	High
	Cerebrovascular	28	1.146 (1.101–1.192)	Mod.	16	1.11 (1.04, 1.18)	High
	ALRI	12	1.204 (1.095–1.325)	High	4	1.16 (1.01, 1.34)	High
	Lung cancer	26	1.093 (1.053–1.135)	High	15	1.12 (1.07, 1.16)	High
	Respiratory	28	1.136 (1.079–1.197)	High	17	1.10 (1.03, 1.18)	Mod.
	COPD	19	1.138 (1.080–1.198)	High	11	1.11 (1.05, 1.17)	High
PM_10_	All-cause	28	1.081 (1.052–1.110)	High	17	1.04 (1.03, 1.06)	High
	Circulatory	26	1.080 (1.042–1.120)	High	15	1.04 (0.99, 1.10)	Mod.
	IHD	16	1.055 (1.019–1.092)	High	13	1.06 (1.01, 1.10)	Mod.
	Cerebrovascular	15	1.049 (0.973–1.131)	Mod.	9	1.01 (0.83, 1.21)	Low
	Lung cancer	17	1.101 (1.052–1.152)	High	13	1.08 (1.04, 1.13)	High
	Respiratory	21	1.122 (1.076–1.169)	High	13	1.12 (1.06, 1.19)	High
	COPD	7	1.215 (1.027–1.438)	High	5	1.19 (0.95, 1.49)	Mod.

N, number of estimates; RR, pooled relative risks; 95% CI, 95% confidence interval; IHD, ischaemic heart disease; ALRI, acute lower respiratory infection; COPD, chronic obstructive pulmonary disease; Mod., moderate.

### Limitations

When interpreting the results of the present study, several limitations must be taken into account, in addition to those of heterogeneity and publication bias already discussed. Statistical tests to evaluate heterogeneity and publication bias have been of limited value, so more qualitative methods were needed in this review to assess these issues, with their limitations related to subjective judgements. Another limitation is that although more recent results have been published from areas outside Canada, the United States and Europe, they still come from countries that are predominantly high- and middle-income economies.

### Conclusion

In conclusion, this study updated the previous systematic review and meta-analysis that informed the 2021 WHO global air quality guidelines on the effects of long-term exposure to PM on mortality, by including a large number of new studies. The results provide up-to-date evidence on the influence of air pollution on mortality, a picture of the rapidly increasing number of studies being conducted on this issue, and an outlook on the challenges of interpreting and appraising the evidence.
