# Supplementary material for: Long-Term Exposure to Particulate Matter and Mortality: An Update of the WHO Global Air Quality Guidelines Systematic Review and Meta-Analysis
Source: Int J Public Health. 2024 Sep 27;69:1607683. doi: 10.3389/ijph.2024.1607683 (PMC11466858; doi:10.3389/ijph.2024.1607683)
Supplement: Supplementary file 6 [file DataSheet1.docx]

*International Journal of Public Health.*

**Long-term exposure to particulate matter and mortality: An update of the WHO global air quality guidelines systematic review and meta-analysis**

Supplementary Material

**Table of Contents**

[**TABLE S1** | Meta-regression based on average pollutant concentration (Global, 2023-2024). 5](#_Toc176438700)

[**TABLE S2** | Certainty of the evidence for PM_10_ and all-cause mortality (Global, 2023-2024). 6](#_Toc176438701)

[**TABLE S3** | Certainty of the evidence for PM_2.5_ and circulatory mortality (Global, 2023-2024). 7](#_Toc176438702)

[**TABLE S4** | Certainty of the evidence for PM_2.5_ and ischaemic heart disease (IHD) mortality (Global, 2023-2024). 8](#_Toc176438703)

[**TABLE S5** | Certainty of the evidence for PM_2.5_ and cerebrovascular mortality (Global, 2023-2024). 9](#_Toc176438704)

[**TABLE S6** | Certainty of the evidence for PM_2.5_ and acute lower respiratory infection (ALRI) mortality (Global, 2023-2024). 10](#_Toc176438705)

[**TABLE S7** | Certainty of the evidence for PM_2.5_ and lung cancer mortality (Global, 2023-2024). 11](#_Toc176438706)

[**TABLE S8** | Certainty of the evidence for PM_2.5_ and respiratory mortality (Global, 2023-2024). 12](#_Toc176438707)

[**TABLE S9** | Certainty of the evidence as assessed for PM_2.5_ and chronic obstructive pulmonary disease (COPD) mortality (Global, 2023-2024). 13](#_Toc176438708)

[**TABLE S10** | Certainty of the evidence for PM_10_ and circulatory mortality (Global, 2023-2024). 14](#_Toc176438709)

[**TABLE S11** | Certainty of the evidence for PM_10_ and ischaemic heart disease (IHD) mortality (Global, 2023-2024). 15](#_Toc176438710)

[**TABLE S12** | Certainty of the evidence for PM_10_ and cerebrovascular mortality (Global, 2023-2024). 16](#_Toc176438711)

[**TABLE S13** | Certainty of the evidence for PM_10_ and lung cancer mortality (Global, 2023-2024). 17](#_Toc176438712)

[**TABLE S14** | Certainty of the evidence for PM_10_ and respiratory mortality (Global, 2023-2024). 18](#_Toc176438713)

[**TABLE S15** | Certainty of the evidence for PM_10_ and chronic obstructive pulmonary disease (COPD) mortality (Global, 2023-2024). 19](#_Toc176438714)

[**TABLE S16** | Summary of certainty of the evidence assessments for each exposure-outcome pair (Global, 2023-2024). 20](#_Toc176438715)

[**FIGURE S1** | Summary of the risk of bias assessment (Global, 2023-2024). 21](#_Toc176438716)

[**FIGURE S2** | Forest plot examining the association between PM_10_ and all-cause mortality (Global, 2023-2024). 22](#_Toc176438717)

[**FIGURE S3** | Forest plot examining the association between PM_2.5_ and circulatory mortality (Global, 2023-2024). 23](#_Toc176438718)

[**FIGURE S4** | Forest plot examining the association between PM_2.5_ and ischaemic heart disease (IHD) mortality (Global, 2023-2024). 24](#_Toc176438719)

[**FIGURE S5** | Forest plot examining the association between PM_2.5_ and cerebrovascular mortality (Global, 2023-2024). 25](#_Toc176438720)

[**FIGURE S6** | Forest plot examining the association between PM_2.5_ and acute lower respiratory infection (ALRI) mortality (Global, 2023-2024). 26](#_Toc176438721)

[**FIGURE S7** | Forest plot examining the association between PM_2.5_ and lung cancer mortality (Global, 2023-2024). 27](#_Toc176438722)

[**FIGURE S8** | Forest plot examining the association between PM_2.5_ and respiratory mortality (Global, 2023-2024). 28](#_Toc176438723)

[**FIGURE S9** | Forest plot examining the association between PM_2.5_ and chronic obstructive pulmonary disease (COPD) mortality (Global, 2023-2024). 29](#_Toc176438724)

[**FIGURE S10** | Forest plot examining the association between PM_10_ and circulatory mortality (Global, 2023-2024). 30](#_Toc176438725)

[**FIGURE S11** | Forest plot examining the association between PM_10_ and ischaemic heart disease (IHD) mortality (Global, 2023-2024). 31](#_Toc176438726)

[**FIGURE S12** | Forest plot examining the association between PM_10_ and cerebrovascular mortality (Global, 2023-2024). 32](#_Toc176438727)

[**FIGURE S13** | Forest plot examining the association between PM_10_ and lung cancer mortality (Global, 2023-2024). 33](#_Toc176438728)

[**FIGURE S14** | Forest plot examining the association between PM_10_ and respiratory mortality (Global, 2023-2024). 34](#_Toc176438729)

[**FIGURE S15** | Forest plot examining the association between PM_10_ and chronic obstructive pulmonary disease (COPD) mortality (Global, 2023-2024). 35](#_Toc176438730)

[**FIGURE S16** | Funnel plot exploring potential publication bias for PM_2.5_ and all-cause mortality (Global, 2023-2024). 36](#_Toc176438731)

[**FIGURE S17** | Funnel plot exploring potential publication bias for PM_10_ and all-cause mortality (Global, 2023-2024). 37](#_Toc176438732)

[**FIGURE S18** | Funnel plot exploring potential publication bias for PM_2.5_ and circulatory mortality (Global, 2023-2024). 38](#_Toc176438733)

[**FIGURE S19** | Funnel plot exploring potential publication bias for PM_2.5_ and ischaemic heart disease (IHD) mortality (Global, 2023-2024). 39](#_Toc176438734)

[**FIGURE S20** | Funnel plot exploring potential publication bias for PM_2.5_ and cerebrovascular mortality (Global, 2023-2024). 40](#_Toc176438735)

[**FIGURE S21** | Funnel plot exploring potential publication bias for PM_2.5_ and acute lower respiratory infection (ALRI) mortality (Global, 2023-2024). 41](#_Toc176438736)

[**FIGURE S22** | Funnel plot exploring potential publication bias for PM_2.5_ and lung cancer mortality (Global, 2023-2024). 42](#_Toc176438737)

[**FIGURE S23** | Funnel plot exploring potential publication bias for PM_2.5_ and respiratory mortality (Global, 2023-2024). 43](#_Toc176438738)

[**FIGURE S24** | Funnel plot exploring potential publication bias for PM_2.5_ and chronic obstructive pulmonary disease (COPD) mortality (Global, 2023-2024). 44](#_Toc176438739)

[**FIGURE S25** | Funnel plot exploring potential publication bias for PM_10_ and circulatory mortality (Global, 2023-2024). 45](#_Toc176438740)

[**FIGURE S26** | Funnel plot exploring potential publication bias for PM_10_ and ischaemic heart disease (IHD) mortality (Global, 2023-2024). 46](#_Toc176438741)

[**FIGURE S27** | Funnel plot exploring potential publication bias for PM_10_ and cerebrovascular mortality (Global, 2023-2024). 47](#_Toc176438742)

[**FIGURE S28** | Funnel plot exploring potential publication bias for PM_10_ and lung cancer mortality (Global, 2023-2024). 48](#_Toc176438743)

[**FIGURE S29** | Funnel plot exploring potential publication bias for PM_10_ and respiratory mortality (Global, 2023-2024). 49](#_Toc176438744)

[**FIGURE S30** | Funnel plot exploring potential publication bias for PM_10_ and chronic obstructive pulmonary disease (COPD) mortality (Global, 2023-2024). 50](#_Toc176438745)

[**FIGURE S31** | Forest plot of the association between PM_2.5_ and all-cause mortality. Subgroup analysis by WHO region: European Region (EUR), Region of the Americas (AMR), Western Pacific Region (WPR) (Global, 2023-2024). 51](#_Toc176438746)

[**FIGURE S32** | Forest plot of the association between PM_10_ and all-cause mortality. Subgroup analysis by WHO region: European Region (EUR), Region of the Americas (AMR), Western Pacific Region (WPR) (Global, 2023-2024). 52](#_Toc176438747)

[**FIGURE S33** | Forest plot of the association between PM_2.5_ and circulatory mortality. Subgroup analysis by WHO region: European Region (EUR), Region of the Americas (AMR), Western Pacific Region (WPR) (Global, 2023-2024). 53](#_Toc176438748)

[**FIGURE S34** | Forest plot of the association between PM_2.5_ and ischaemic heart disease (IHD) mortality. Subgroup analysis by WHO region: European Region (EUR), Region of the Americas (AMR), Western Pacific Region (WPR) (Global, 2023-2024). 54](#_Toc176438749)

[**FIGURE S35** | Forest plot of the association between PM_2.5_ and cerebrovascular mortality. Subgroup analysis by WHO region: European Region (EUR), Region of the Americas (AMR), Western Pacific Region (WPR) (Global, 2023-2024). 55](#_Toc176438750)

[**FIGURE S36** | Forest plot of the association between PM_2.5_ and acute lower respiratory infection (ALRI) mortality. Subgroup analysis by WHO region: Region of the Americas (AMR), Western Pacific Region (WPR) (Global, 2023-2024). 56](#_Toc176438751)

[**FIGURE S37** | Forest plot of the association between PM_2.5_ and lung cancer mortality. Subgroup analysis by WHO region: European Region (EUR), Region of the Americas (AMR), Western Pacific Region (WPR) (Global, 2023-2024). 57](#_Toc176438752)

[**FIGURE S38** | Forest plot of the association between PM_2.5_ and respiratory mortality. Subgroup analysis by WHO region: European Region (EUR), Region of the Americas (AMR), Western Pacific Region (WPR) (Global, 2023-2024). 58](#_Toc176438753)

[**FIGURE S39** | Forest plot of the association between PM_2.5_ and chronic obstructive pulmonary disease (COPD) mortality. Subgroup analysis by WHO region: European Region (EUR), Region of the Americas (AMR), Western Pacific Region (WPR) (Global, 2023-2024). 59](#_Toc176438754)

[**FIGURE S40** | Forest plot of the association between PM_10_ and circulatory mortality. Subgroup analysis by WHO region: European Region (EUR), Western Pacific Region (WPR) (Global, 2023-2024). 60](#_Toc176438755)

[**FIGURE S41** | Forest plot of the association between PM_10_ and ischaemic heart disease (IHD) mortality. Subgroup analysis by WHO region: European Region (EUR), Region of the Americas (AMR), Western Pacific Region (WPR) (Global, 2023-2024). 61](#_Toc176438756)

[**FIGURE S42** | Forest plot of the association between PM_10_ and cerebrovascular mortality. Subgroup analysis by WHO region: European Region (EUR), Western Pacific Region (WPR) (Global, 2023-2024). 62](#_Toc176438757)

[**FIGURE S43** | Forest plot of the association between PM_10_ and lung cancer mortality. Subgroup analysis by WHO region: European Region (EUR), Region of the Americas (AMR), Western Pacific Region (WPR) (Global, 2023-2024). 63](#_Toc176438758)

[**FIGURE S44** | Forest plot of the association between PM_10_ and respiratory mortality. Subgroup analysis by WHO region: European Region (EUR), Region of the Americas (AMR), Western Pacific Region (WPR) (Global, 2023-2024). 64](#_Toc176438759)

[**FIGURE S45** | Forest plot of the association between PM_10_ and chronic obstructive pulmonary disease (COPD) mortality. Subgroup analysis by WHO region: European Region (EUR), Western Pacific Region (WPR) (Global, 2023-2024). 65](#_Toc176438760)

[**TABLE S17a** | PRISMA checklist (Global, 2023-2024). 66](#_Toc176438761)

[**TABLE S17b** | PRISMA checklist for abstracts (Global, 2023-2024). 69](#_Toc176438762)

[**TABLE S18** | Search strategy (Global, 2023-2024). 70](#_Toc176438763)

[**TABLE S19** | Causes of death included in the review, by category and subcategory (Global, 2023-2024). 71](#_Toc176438764)

[**TABLE S20** | Pooled effect sizes for PM_2.5_ and all-cause and cause-specific mortality. Subgroup analysis by study size (Global, 2023-2024). 72](#_Toc176438765)

[**TABLE S21** | Pooled effect sizes for PM_10_ and all-cause and cause-specific mortality. Subgroup analysis by study size (Global, 2023-2024). 73](#_Toc176438766)

[**TABLE S22** | Description of the certainty of evidence tool and criteria (Global, 2023-2024). 74](#_Toc176438767)

[**TABLE S23** | Overall results of the risk of bias assessment by domain (Global, 2023-2024). 75](#_Toc176438768)

[**TABLE S24** | Results of the certainty of evidence assessment by domain (Global, 2023-2024). 76](#_Toc176438769)

### **TABLE S1** | Meta-regression based on average pollutant concentration (Global, 2023-2024).

| **Pollutant** | **Outcome (mortality)** | **N** | **Coef.** | **SE** | **95% CI** | **p-value** |
| --- | --- | --- | --- | --- | --- | --- |
| PM_2.5_ | All-cause | 51 | -0.0003 | 0.0008 | -0.0020 – 0.0013 | 0.70 |
|  | Circulatory | 39 | -0.0002 | 0.0007 | -0.0016 – 0.0012 | 0.81 |
|  | IHD | 30 | 0.0011 | 0.0013 | -0.0015 – 0.0038 | 0.40 |
|  | Cerebrovascular | 25 | 0.0004 | 0.0013 | -0.0021 – 0.0029 | 0.78 |
|  | ALRI | 10 | -0.0048 | 0.0051 | -0.0148 – 0.0051 | 0.34 |
|  | Lung cancer | 24 | 0.0019 | 0.0011 | -0.0002 – 0.0040 | 0.08 |
|  | Respiratory | 26 | -0.0005 | 0.0020 | -0.0044 – 0.0034 | 0.79 |
|  | COPD | 18 | 0.0023 | 0.0024 | -0.0024 – 0.0070 | 0.34 |
| PM_10_ | All-cause | 23 | 0.0003 | 0.0007 | -0.0011 – 0.0016 | 0.71 |
|  | Circulatory | 21 | 0.0017 | 0.0008 | 0.0001 – 0.0033 | 0.03 |
|  | IHD | 11 | 0.0034 | 0.0021 | -0.0008 – 0.0076 | 0.12 |
|  | Cerebrovascular | 10 | 0.0031 | 0.0009 | 0.0013 – 0.0048 | <0.01 |
|  | ALRI | 1 | N/A | N/A | N/A | N/A |
|  | Lung cancer | 12 | -0.0012 | 0.0010 | -0.0032 – 0.0007 | 0.23 |
|  | Respiratory | 17 | 0.0010 | 0.0009 | -0.0009 – 0.0028 | 0.30 |
|  | COPD | 6 | N/A | N/A | N/A | N/A |

Coef., regression coefficient; SE, standard error: 95% CI, 95% confidence interval for the regression coefficient; IHD, ischaemic heart disease; ALRI, acute lower respiratory infection; COPD, chronic obstructive pulmonary disease; N/A, not applicable (< 10 studies).

### **TABLE S2** | Certainty of the evidence for PM_10_ and all-cause mortality (Global, 2023-2024).

| **Domain** | **Judgement** | **Downgrade/upgrade and conclusion** |
| --- | --- | --- |
| Starting point assessment |  | Moderate certainty of evidence (3/4) |
| Limitations in studies | Excluding studies with high risk of bias had little effect on pooled effects. | No downgrading (0) |
| Indirectness | All studies addressed the PECOS question directly. | No downgrading (0) |
| Inconsistency | Heterogeneity was suspected based on the results of the 80% prediction interval. However, the studies showed consistent positive associations. | No downgrading (0) |
| Imprecision | Pooled effects calculated using more than 940,000 person-years. | No downgrading (0) |
| Publication bias | The funnel plot showed asymmetries, but there were no significant differences between smaller and larger studies. | No downgrading (0) |
| Large effect size | The pooled RR is not large enough to rule out the possibility of unmeasured confounding by other factors. | No upgrading (0) |
| All plausible confounding biases RR to zero | Several potential confounders that would shift the RR in either both directions. | No upgrading (0) |
| Concentration-response gradient | Positive and significant association found in the main analysis. | Upgrade one level (+1) |
| Conclusion |  | **HIGH CERTAINTY OF EVIDENCE (4/4)** |

PECOS, population, exposure, comparator, outcome and study; RR, relative risk.

### **TABLE S3** | Certainty of the evidence for PM_2.5_ and circulatory mortality (Global, 2023-2024).

| **Domain** | **Judgement** | **Downgrade/upgrade and conclusion** |
| --- | --- | --- |
| Starting point assessment |  | Moderate certainty of evidence (3/4) |
| Limitations in studies | Excluding studies with high risk of bias had little effect on pooled effects. | No downgrading (0) |
| Indirectness | All studies addressed the PECOS question directly. | No downgrading (0) |
| Inconsistency | No evidence of heterogeneity provided by the 80% prediction interval. | No downgrading (0) |
| Imprecision | Pooled effects calculated using more than 940,000 person-years. | No downgrading (0) |
| Publication bias | The funnel plot showed asymmetries, and there were significant differences between smaller and larger studies. | Downgrade one level (-1) |
| Large effect size | The pooled RR is not large enough to rule out the possibility of unmeasured confounding by other factors. | No upgrading (0) |
| All plausible confounding biases RR to zero | Several potential confounders that would shift the RR in either both directions. | No upgrading (0) |
| Concentration-response gradient | Positive and significant association found in the main analysis. | Upgrade one level (+1) |
| Conclusion |  | **MODERATE CERTAINTY OF EVIDENCE (3/4)** |

PECOS, population, exposure, comparator, outcome and study; RR, relative risk.

### **TABLE S4** | Certainty of the evidence for PM_2.5_ and ischaemic heart disease (IHD) mortality (Global, 2023-2024).

| **Domain** | **Judgement** | **Downgrade/upgrade and conclusion** |
| --- | --- | --- |
| Starting point assessment |  | Moderate certainty of evidence (3/4) |
| Limitations in studies | Excluding studies with high risk of bias had little effect on pooled effects. | No downgrading (0) |
| Indirectness | All studies addressed the PECOS question directly. | No downgrading (0) |
| Inconsistency | No evidence of heterogeneity provided by the 80% prediction interval. | No downgrading (0) |
| Imprecision | Pooled effects calculated using more than 940,000 person-years. | No downgrading (0) |
| Publication bias | The funnel plot showed asymmetries, but there were no significant differences between smaller and larger studies. | No downgrading (0) |
| Large effect size | The pooled RR is not large enough to rule out the possibility of unmeasured confounding by other factors. | No upgrading (0) |
| All plausible confounding biases RR to zero | Several potential confounders that would shift the RR in either both directions. | No upgrading (0) |
| Concentration-response gradient | Positive and significant association found in the main analysis. | Upgrade one level (+1) |
| Conclusion |  | **HIGH CERTAINTY OF EVIDENCE (4/4)** |

PECOS, population, exposure, comparator, outcome and study; RR, relative risk.

### **TABLE S5** | Certainty of the evidence for PM_2.5_ and cerebrovascular mortality (Global, 2023-2024).

| **Domain** | **Judgement** | **Downgrade/upgrade and conclusion** |
| --- | --- | --- |
| Starting point assessment |  | Moderate certainty of evidence (3/4) |
| Limitations in studies | Excluding studies with high risk of bias had little effect on pooled effects. | No downgrading (0) |
| Indirectness | All studies addressed the PECOS question directly. | No downgrading (0) |
| Inconsistency | No evidence of heterogeneity provided by the 80% prediction interval. | No downgrading (0) |
| Imprecision | Pooled effects calculated using more than 940,000 person-years. | No downgrading (0) |
| Publication bias | The funnel plot showed asymmetries, and there were significant differences between smaller and larger studies. | Downgrade one level (-1) |
| Large effect size | The pooled RR is not large enough to rule out the possibility of unmeasured confounding by other factors. | No upgrading (0) |
| All plausible confounding biases RR to zero | Several potential confounders that would shift the RR in either both directions. | No upgrading (0) |
| Concentration-response gradient | Positive and significant association found in the main analysis. | Upgrade one level (+1) |
| Conclusion |  | **MODERATE CERTAINTY OF EVIDENCE (3/4)** |

PECOS, population, exposure, comparator, outcome and study; RR, relative risk.

### **TABLE S6** | Certainty of the evidence for PM_2.5_ and acute lower respiratory infection (ALRI) mortality (Global, 2023-2024).

| **Domain** | **Judgement** | **Downgrade/upgrade and conclusion** |
| --- | --- | --- |
| Starting point assessment |  | Moderate certainty of evidence (3/4) |
| Limitations in studies | Excluding studies with high risk of bias had little effect on pooled effects. | No downgrading (0) |
| Indirectness | All studies addressed the PECOS question directly. | No downgrading (0) |
| Inconsistency | No evidence of heterogeneity provided by the 80% prediction interval. | No downgrading (0) |
| Imprecision | Pooled effects calculated using more than 940,000 person-years. | No downgrading (0) |
| Publication bias | The funnel plot showed no asymmetry. | No downgrading (0) |
| Large effect size | The pooled RR is not large enough to rule out the possibility of unmeasured confounding by other factors. | No upgrading (0) |
| All plausible confounding biases RR to zero | Several potential confounders that would shift the RR in either both directions. | No upgrading (0) |
| Concentration-response gradient | Positive and significant association found in the main analysis. | Upgrade one level (+1) |
| Conclusion |  | **HIGH CERTAINTY OF EVIDENCE (4/4)** |

PECOS, population, exposure, comparator, outcome and study; RR, relative risk.

### **TABLE S7** | Certainty of the evidence for PM_2.5_ and lung cancer mortality (Global, 2023-2024).

| **Domain** | **Judgement** | **Downgrade/upgrade and conclusion** |
| --- | --- | --- |
| Starting point assessment |  | Moderate certainty of evidence (3/4) |
| Limitations in studies | Excluding studies with high risk of bias had little effect on pooled effects. | No downgrading (0) |
| Indirectness | All studies addressed the PECOS question directly. | No downgrading (0) |
| Inconsistency | Some evidence of heterogeneity, given the wide range of the 80% prediction interval. However, this heterogeneity may be related to geographical differences, as seen in the subgroup analysis. As the heterogeneity was partially explained, no downgrading was applied. | No downgrading (0) |
| Imprecision | Pooled effects calculated using more than 940,000 person-years. | No downgrading (0) |
| Publication bias | The funnel plot showed asymmetries, but there were no significant differences between smaller and larger studies. | No downgrading (0) |
| Large effect size | The pooled RR is not large enough to rule out the possibility of unmeasured confounding by other factors. | No upgrading (0) |
| All plausible confounding biases RR to zero | Several potential confounders that would shift the RR in either both directions. | No upgrading (0) |
| Concentration-response gradient | Positive and significant association found in the main analysis. | Upgrade one level (+1) |
| Conclusion |  | **HIGH CERTAINTY OF EVIDENCE (4/4)** |

PECOS, population, exposure, comparator, outcome and study; RR, relative risk.

### **TABLE S8** | Certainty of the evidence for PM_2.5_ and respiratory mortality (Global, 2023-2024).

| **Domain** | **Judgement** | **Downgrade/upgrade and conclusion** |
| --- | --- | --- |
| Starting point assessment |  | Moderate certainty of evidence (3/4) |
| Limitations in studies | Excluding studies with high risk of bias had little effect on pooled effects. | No downgrading (0) |
| Indirectness | All studies addressed the PECOS question directly. | No downgrading (0) |
| Inconsistency | Heterogeneity was suspected based on the results of the 80% prediction interval. However, the studies showed consistent positive associations. | No downgrading (0) |
| Imprecision | Pooled effects calculated using more than 940,000 person-years. | No downgrading (0) |
| Publication bias | The funnel plot showed asymmetries, but there were no significant differences between smaller and larger studies. | No downgrading (0) |
| Large effect size | The pooled RR is not large enough to rule out the possibility of unmeasured confounding by other factors. | No upgrading (0) |
| All plausible confounding biases RR to zero | Several potential confounders that would shift the RR in either both directions. | No upgrading (0) |
| Concentration-response gradient | Positive and significant association found in the main analysis. | Upgrade one level (+1) |
| Conclusion |  | **HIGH CERTAINTY OF EVIDENCE (4/4)** |

PECOS, population, exposure, comparator, outcome and study; RR, relative risk.

### **TABLE S9** | Certainty of the evidence as assessed for PM_2.5_ and chronic obstructive pulmonary disease (COPD) mortality (Global, 2023-2024).

| **Domain** | **Judgement** | **Downgrade/upgrade and conclusion** |
| --- | --- | --- |
| Starting point assessment |  | Moderate certainty of evidence (3/4) |
| Limitations in studies | Excluding studies with high risk of bias had little effect on pooled effects. | No downgrading (0) |
| Indirectness | All studies addressed the PECOS question directly. | No downgrading (0) |
| Inconsistency | No evidence of heterogeneity provided by the 80% prediction interval. | No downgrading (0) |
| Imprecision | Pooled effects calculated using more than 940,000 person-years. | No downgrading (0) |
| Publication bias | The funnel plot showed no asymmetry. | No downgrading (0) |
| Large effect size | The pooled RR is not large enough to rule out the possibility of unmeasured confounding by other factors. | No upgrading (0) |
| All plausible confounding biases RR to zero | Several potential confounders that would shift the RR in either both directions. | No upgrading (0) |
| Concentration-response gradient | Positive and significant association found in the main analysis. | Upgrade one level (+1) |
| Conclusion |  | **HIGH CERTAINTY OF EVIDENCE (4/4)** |

PECOS, population, exposure, comparator, outcome and study; RR, relative risk.

### **TABLE S10** | Certainty of the evidence for PM_10_ and circulatory mortality (Global, 2023-2024).

| **Domain** | **Judgement** | **Downgrade/upgrade and conclusion** |
| --- | --- | --- |
| Starting point assessment |  | Moderate certainty of evidence (3/4) |
| Limitations in studies | Excluding studies with high risk of bias had little effect on pooled effects. | No downgrading (0) |
| Indirectness | All studies addressed the PECOS question directly. | No downgrading (0) |
| Inconsistency | Some evidence of heterogeneity, given the wide range of the 80% prediction interval. However, this heterogeneity may be related to differences in the ambient levels of the pollutant, as seen in the meta-regression analysis. As the heterogeneity was partially explained, no downgrading was applied. | No downgrading (0) |
| Imprecision | Pooled effects calculated using more than 940,000 person-years. | No downgrading (0) |
| Publication bias | The funnel plot showed no asymmetry. | No downgrading (0) |
| Large effect size | The pooled RR is not large enough to rule out the possibility of unmeasured confounding by other factors. | No upgrading (0) |
| All plausible confounding biases RR to zero | Several potential confounders that would shift the RR in either both directions. | No upgrading (0) |
| Concentration-response gradient | Positive and significant association found in the main analysis. | Upgrade one level (+1) |
| Conclusion |  | **HIGH CERTAINTY OF EVIDENCE (4/4)** |

PECOS, population, exposure, comparator, outcome and study; RR, relative risk.

### **TABLE S11** | Certainty of the evidence for PM_10_ and ischaemic heart disease (IHD) mortality (Global, 2023-2024).

| **Domain** | **Judgement** | **Downgrade/upgrade and conclusion** |
| --- | --- | --- |
| Starting point assessment |  | Moderate certainty of evidence (3/4) |
| Limitations in studies | Excluding studies with high risk of bias had little effect on pooled effects. | No downgrading (0) |
| Indirectness | All studies addressed the PECOS question directly. | No downgrading (0) |
| Inconsistency | The range of the 80% prediction interval includes unity. However, this interval is less than 2 times the 95% confidence interval. In this case, no downgrade was applied. | No downgrading (0) |
| Imprecision | Pooled effects calculated using more than 940,000 person-years. | No downgrading (0) |
| Publication bias | The funnel plot showed no asymmetry. | No downgrading (0) |
| Large effect size | The pooled RR is not large enough to rule out the possibility of unmeasured confounding by other factors. | No upgrading (0) |
| All plausible confounding biases RR to zero | Several potential confounders that would shift the RR in either both directions. | No upgrading (0) |
| Concentration-response gradient | Positive and significant association found in the main analysis. | Upgrade one level (+1) |
| Conclusion |  | **HIGH CERTAINTY OF EVIDENCE (4/4)** |

PECOS, population, exposure, comparator, outcome and study; RR, relative risk.

### **TABLE S12** | Certainty of the evidence for PM_10_ and cerebrovascular mortality (Global, 2023-2024).

| **Domain** | **Judgement** | **Downgrade/upgrade and conclusion** |
| --- | --- | --- |
| Starting point assessment |  | Moderate certainty of evidence (3/4) |
| Limitations in studies | Excluding studies with high risk of bias had little effect on pooled effects. | No downgrading (0) |
| Indirectness | All studies addressed the PECOS question directly. | No downgrading (0) |
| Inconsistency | Some evidence of heterogeneity, given the wide range of the 80% prediction interval. However, this heterogeneity may be related to differences in the ambient levels of the pollutant, as seen in the meta-regression analysis. As the heterogeneity was partially explained, no downgrading was applied. | No downgrading (0) |
| Imprecision | Pooled effects calculated using more than 940,000 person-years. | No downgrading (0) |
| Publication bias | The funnel plot showed no asymmetry. | No downgrading (0) |
| Large effect size | The pooled RR is not large enough to rule out the possibility of unmeasured confounding by other factors. | No upgrading (0) |
| All plausible confounding biases RR to zero | Several potential confounders that would shift the RR in either both directions. | No upgrading (0) |
| Concentration-response gradient | The association between exposure and outcome is positive but not significant. | No upgrading (0) |
| Conclusion |  | **MODERATE CERTAINTY OF EVIDENCE (3/4)** |

PECOS, population, exposure, comparator, outcome and study; RR, relative risk.

### **TABLE S13** | Certainty of the evidence for PM_10_ and lung cancer mortality (Global, 2023-2024).

| **Domain** | **Judgement** | **Downgrade/upgrade and conclusion** |
| --- | --- | --- |
| Starting point assessment |  | Moderate certainty of evidence (3/4) |
| Limitations in studies | Excluding studies with high risk of bias had little effect on pooled effects. | No downgrading (0) |
| Indirectness | All studies addressed the PECOS question directly. | No downgrading (0) |
| Inconsistency | Some evidence of heterogeneity, given the wide range of the 80% prediction interval. However, this heterogeneity may be related to geographical differences, as seen in the subgroup analysis. As the heterogeneity was partially explained, no downgrading was applied. | No downgrading (0) |
| Imprecision | Pooled effects calculated using more than 940,000 person-years. | No downgrading (0) |
| Publication bias | The funnel plot showed no asymmetry. | No downgrading (0) |
| Large effect size | The pooled RR is not large enough to rule out the possibility of unmeasured confounding by other factors. | No upgrading (0) |
| All plausible confounding biases RR to zero | Several potential confounders that would shift the RR in either both directions. | No upgrading (0) |
| Concentration-response gradient | Positive and significant association found in the main analysis. | Upgrade one level (+1) |
| Conclusion |  | **HIGH CERTAINTY OF EVIDENCE (4/4)** |

PECOS, population, exposure, comparator, outcome and study; RR, relative risk.

### **TABLE S14** | Certainty of the evidence for PM_10_ and respiratory mortality (Global, 2023-2024).

| **Domain** | **Judgement** | **Downgrade/upgrade and conclusion** |
| --- | --- | --- |
| Starting point assessment |  | Moderate certainty of evidence (3/4) |
| Limitations in studies | Excluding studies with high risk of bias had little effect on pooled effects. | No downgrading (0) |
| Indirectness | All studies addressed the PECOS question directly. | No downgrading (0) |
| Inconsistency | No evidence of heterogeneity provided by the 80% prediction interval. | No downgrading (0) |
| Imprecision | Pooled effects calculated using more than 940,000 person-years. | No downgrading (0) |
| Publication bias | The funnel plot showed no asymmetry. | No downgrading (0) |
| Large effect size | The pooled RR is not large enough to rule out the possibility of unmeasured confounding by other factors. | No upgrading (0) |
| All plausible confounding biases RR to zero | Several potential confounders that would shift the RR in either both directions. | No upgrading (0) |
| Concentration-response gradient | Positive and significant association found in the main analysis. | Upgrade one level (+1) |
| Conclusion |  | **HIGH CERTAINTY OF EVIDENCE (4/4)** |

PECOS, population, exposure, comparator, outcome and study; RR, relative risk.

### **TABLE S15** | Certainty of the evidence for PM_10_ and chronic obstructive pulmonary disease (COPD) mortality (Global, 2023-2024).

| **Domain** | **Judgement** | **Downgrade/upgrade and conclusion** |
| --- | --- | --- |
| Starting point assessment |  | Moderate certainty of evidence (3/4) |
| Limitations in studies | When two studies with a high risk of confounding bias were excluded, the results were not significant, but the association was still positive. | No downgrading (0) |
| Indirectness | All studies addressed the PECOS question directly. | No downgrading (0) |
| Inconsistency | The range of the 80% prediction interval includes unity. However, this interval is less than 2 times the 95% confidence interval. In this case, no downgrade was applied. | No downgrading (0) |
| Imprecision | Pooled effects calculated using more than 940,000 person-years. | No downgrading (0) |
| Publication bias | Fewer than 10 studies were included, making it difficult to interpret the funnel plot. Only small studies were included. There was no evidence to confirm or reject publication bias. No downgrade was applied. | No downgrading (0) |
| Large effect size | The pooled RR is not large enough to rule out the possibility of unmeasured confounding by other factors. | No upgrading (0) |
| All plausible confounding biases RR to zero | Several potential confounders that would shift the RR in either both directions. | No upgrading (0) |
| Concentration-response gradient | Positive and significant association found in the main analysis. | Upgrade one level (+1) |
| Conclusion |  | **HIGH CERTAINTY OF EVIDENCE (4/4)** |

PECOS, population, exposure, comparator, outcome and study; RR, relative risk.

### **TABLE S16** | Summary of certainty of the evidence assessments for each exposure-outcome pair (Global, 2023-2024).

| **Pollutant** | **Outcome** | **Limitations in studies** |  | **Indirectness** | **Inconsistency** | **Imprecision** | **Publication bias** | **Large effect size** | **All plausible confounding biases RR to zero** | **Concentration-response gradient** | **Conclusion** |
| --- | --- | --- | --- | --- | --- | --- | --- | --- | --- | --- | --- |
| PM_2.5_ | All-cause | (0) |  | (0) | (0) | (0) | (0) | (0) | (0) | (+1) | **High (4/4)** |
|  | Circulatory | (0) |  | (0) | (0) | (0) | (-1) | (0) | (0) | (+1) | **Mod. (3/4)** |
|  | IHD | (0) |  | (0) | (0) | (0) | (0) | (0) | (0) | (+1) | **High (4/4)** |
|  | Cerebrovascular | (0) |  | (0) | (0) | (0) | (-1) | (0) | (0) | (+1) | **Mod. (3/4)** |
|  | ALRI | (0) |  | (0) | (0) | (0) | (0) | (0) | (0) | (+1) | **High (4/4)** |
|  | Lung cancer | (0) |  | (0) | (0) | (0) | (0) | (0) | (0) | (+1) | **High (4/4)** |
|  | Respiratory | (0) |  | (0) | (0) | (0) | (0) | (0) | (0) | (+1) | **High (4/4)** |
|  | COPD | (0) |  | (0) | (0) | (0) | (0) | (0) | (0) | (+1) | **High (4/4)** |
| PM_10_ | All-cause | (0) |  | (0) | (0) | (0) | (0) | (0) | (0) | (+1) | **High. (4/4)** |
|  | Circulatory | (0) |  | (0) | (0) | (0) | (0) | (0) | (0) | (+1) | **High (4/4)** |
|  | IHD | (0) |  | (0) | (0) | (0) | (0) | (0) | (0) | (+1) | **High (4/4)** |
|  | Cerebrovascular | (0) |  | (0) | (0) | (0) | (0) | (0) | (0) | (0) | **Mod. (3/4)** |
|  | Lung cancer | (0) |  | (0) | (0) | (0) | (0) | (0) | (0) | (+1) | **High (4/4)** |
|  | Respiratory | (0) |  | (0) | (0) | (0) | (0) | (0) | (0) | (+1) | **High (4/4)** |
|  | COPD | (0) |  | (0) | (0) | (0) | (0) | (0) | (0) | (+1) | **High. (4/4)** |

IHD, ischaemic heart disease; ALRI, acute lower respiratory infection; COPD, chronic obstructive pulmonary disease; Mod., moderate.

**
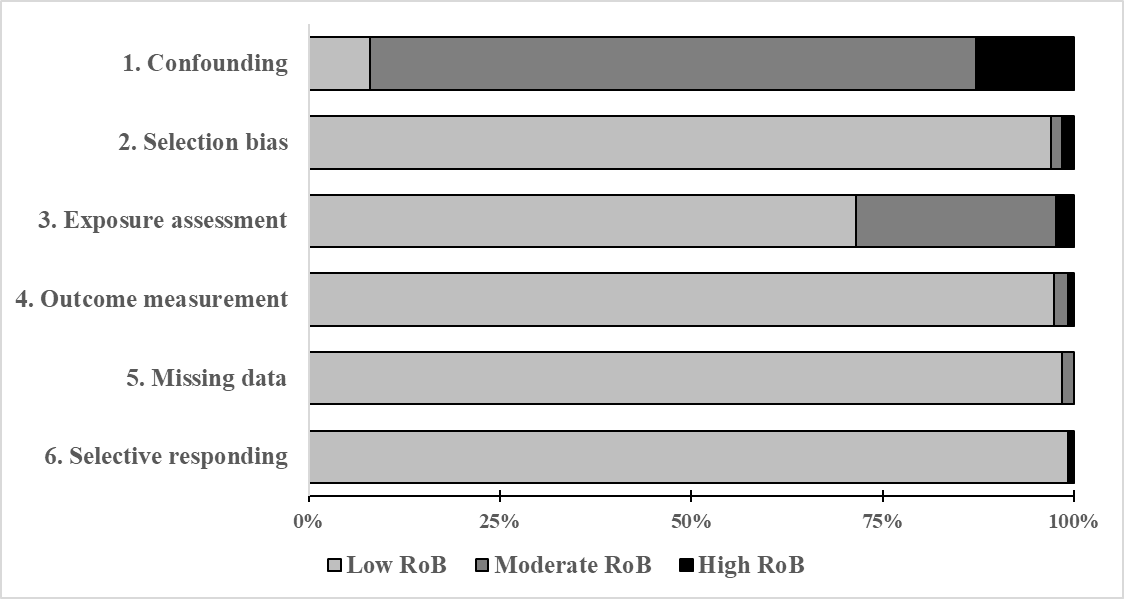
**

### **FIGURE S1** | Summary of the risk of bias assessment (Global, 2023-2024).

**
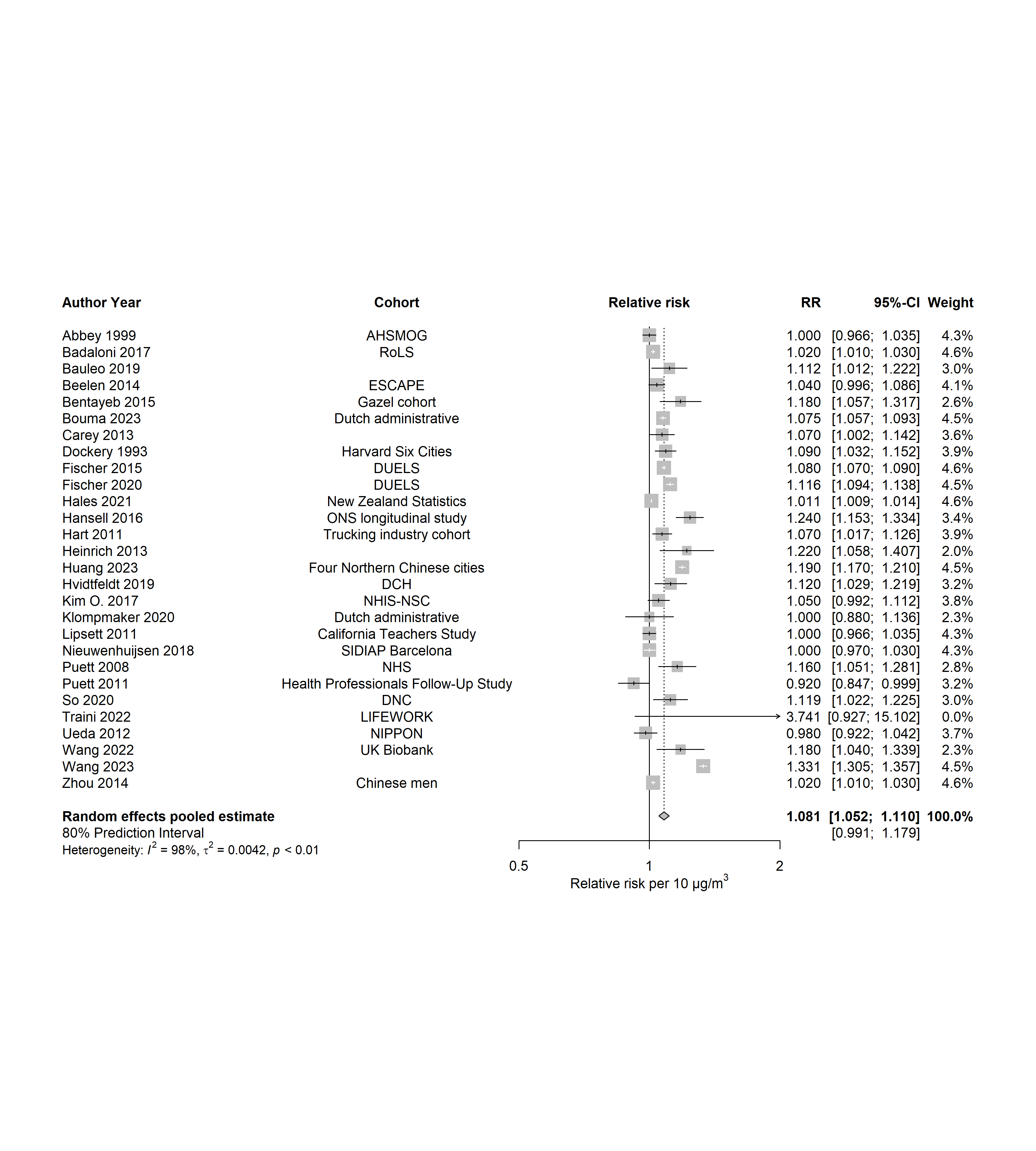
**

### **FIGURE S2** | Forest plot examining the association between PM_10_ and all-cause mortality (Global, 2023-2024).

**
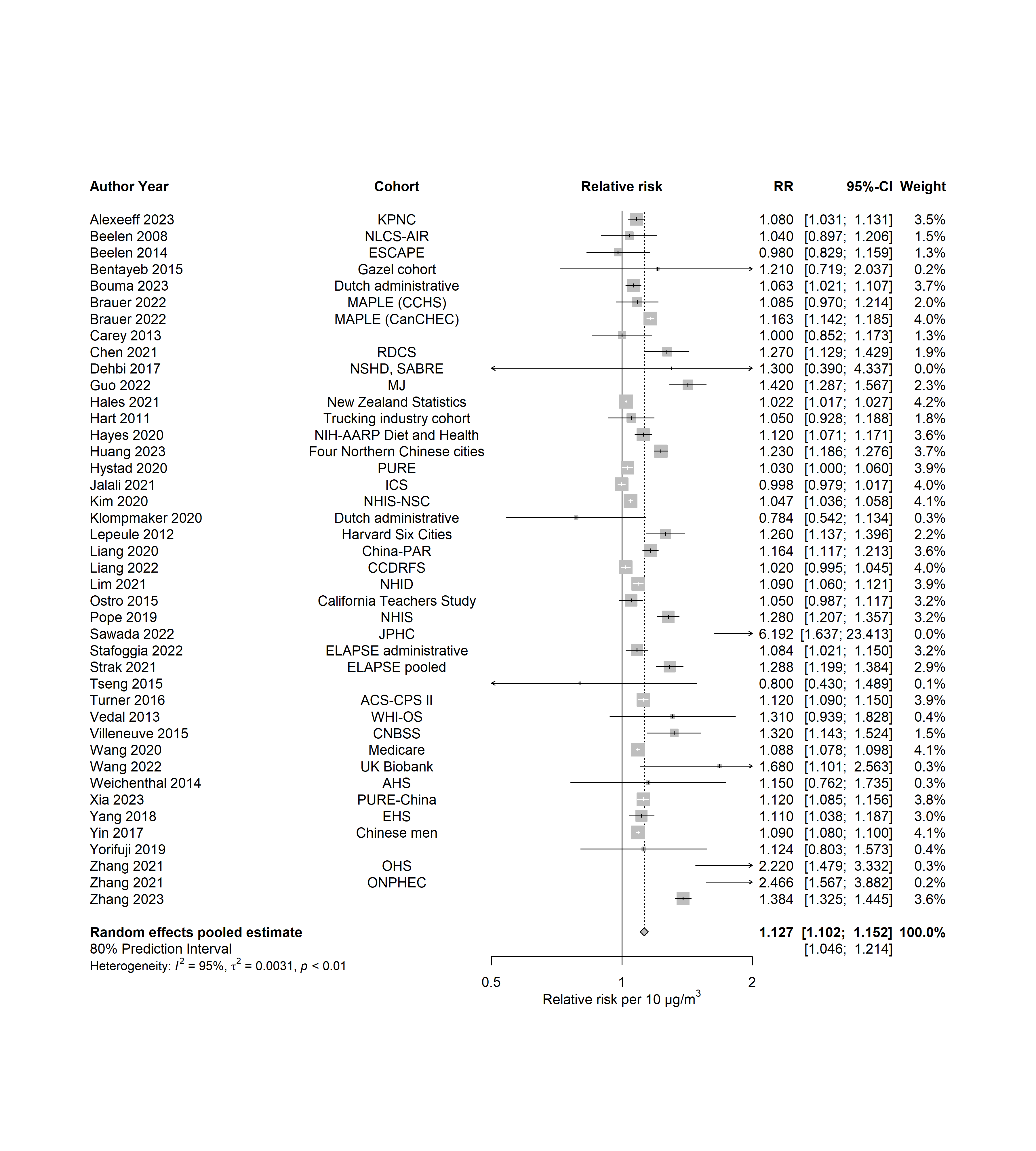
**

### **FIGURE S3** | Forest plot examining the association between PM_2.5_ and circulatory mortality (Global, 2023-2024).


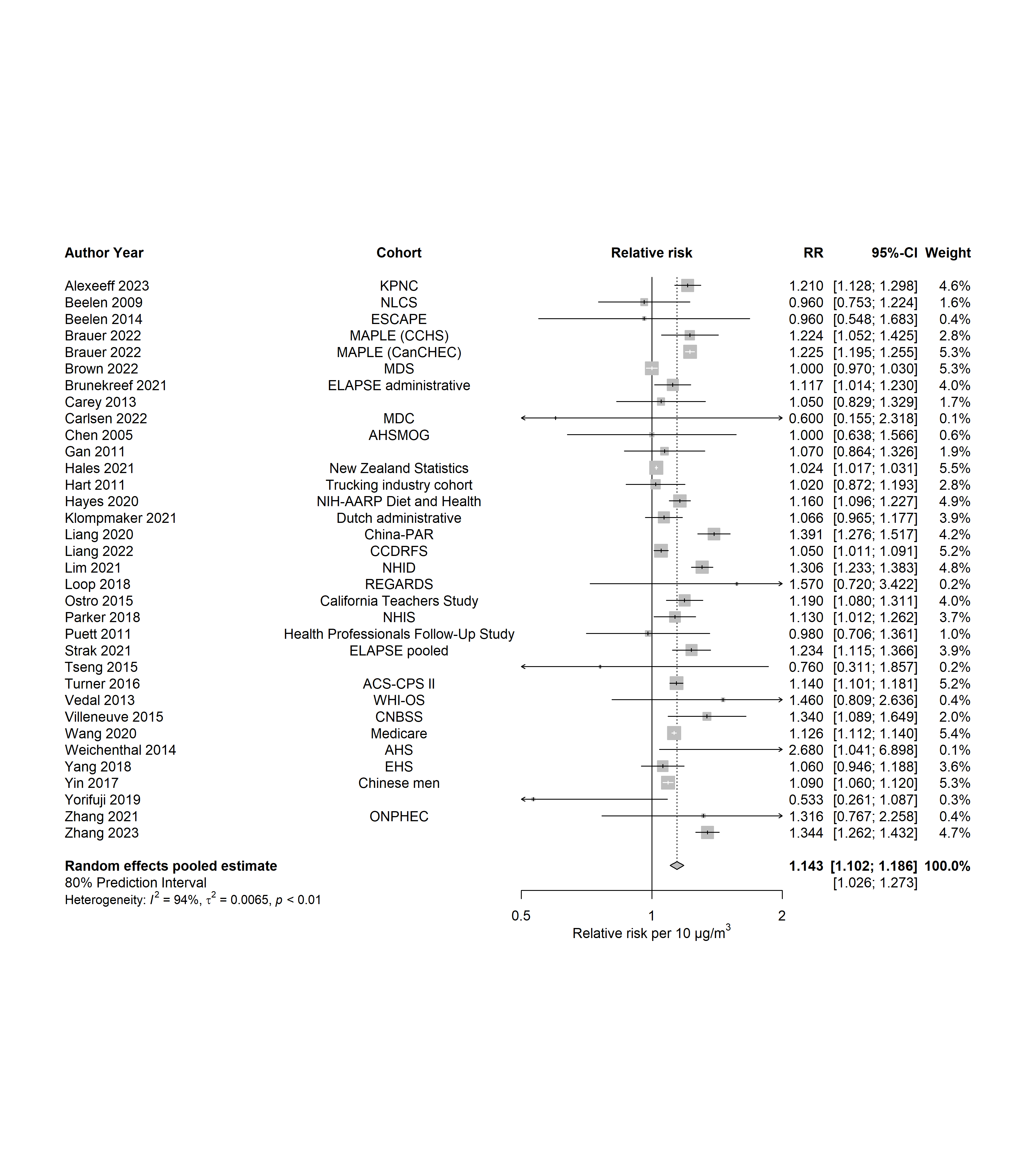


### **FIGURE S4** | Forest plot examining the association between PM_2.5_ and ischaemic heart disease (IHD) mortality (Global, 2023-2024).


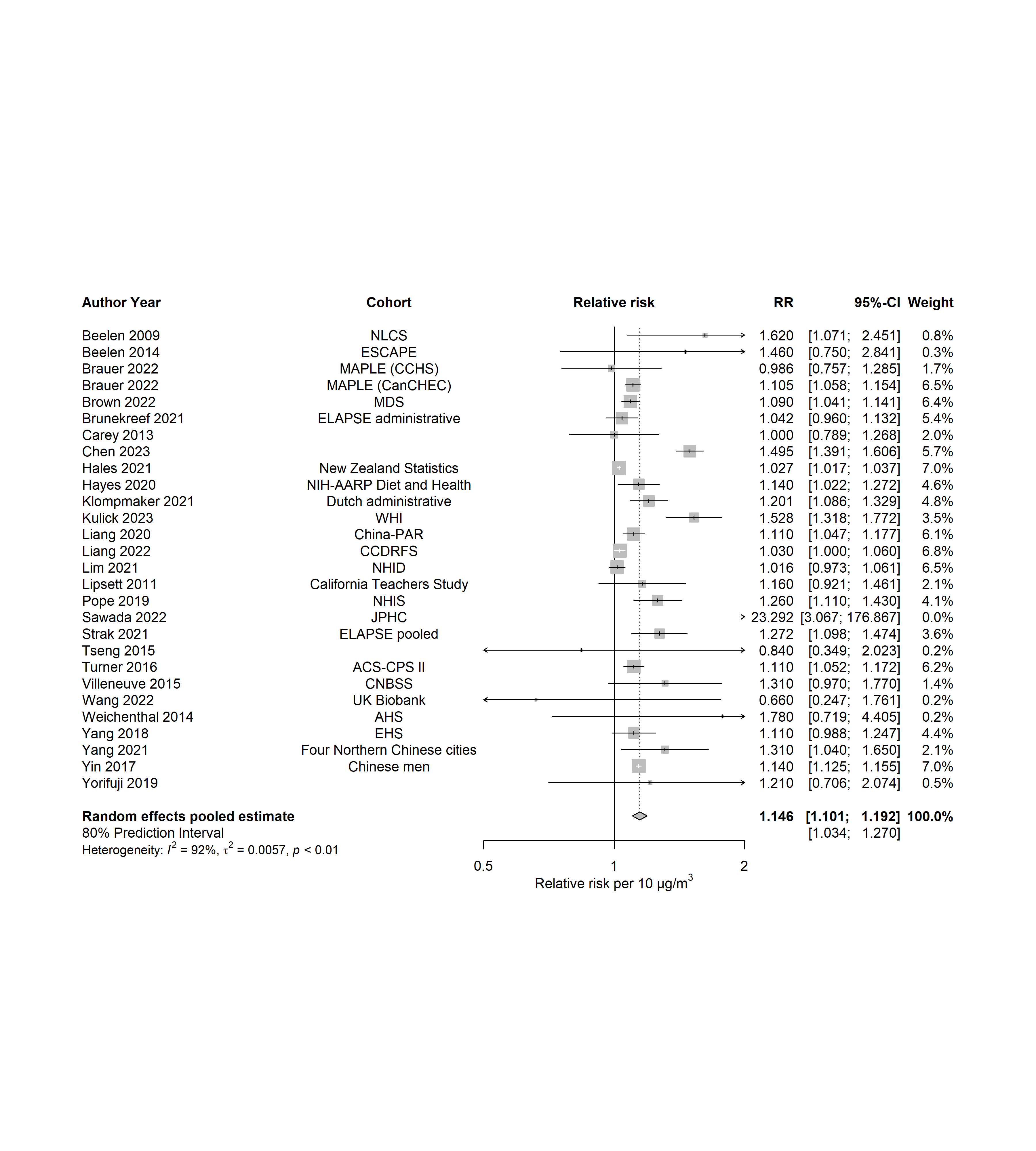


### **FIGURE S5** | Forest plot examining the association between PM_2.5_ and cerebrovascular mortality (Global, 2023-2024).


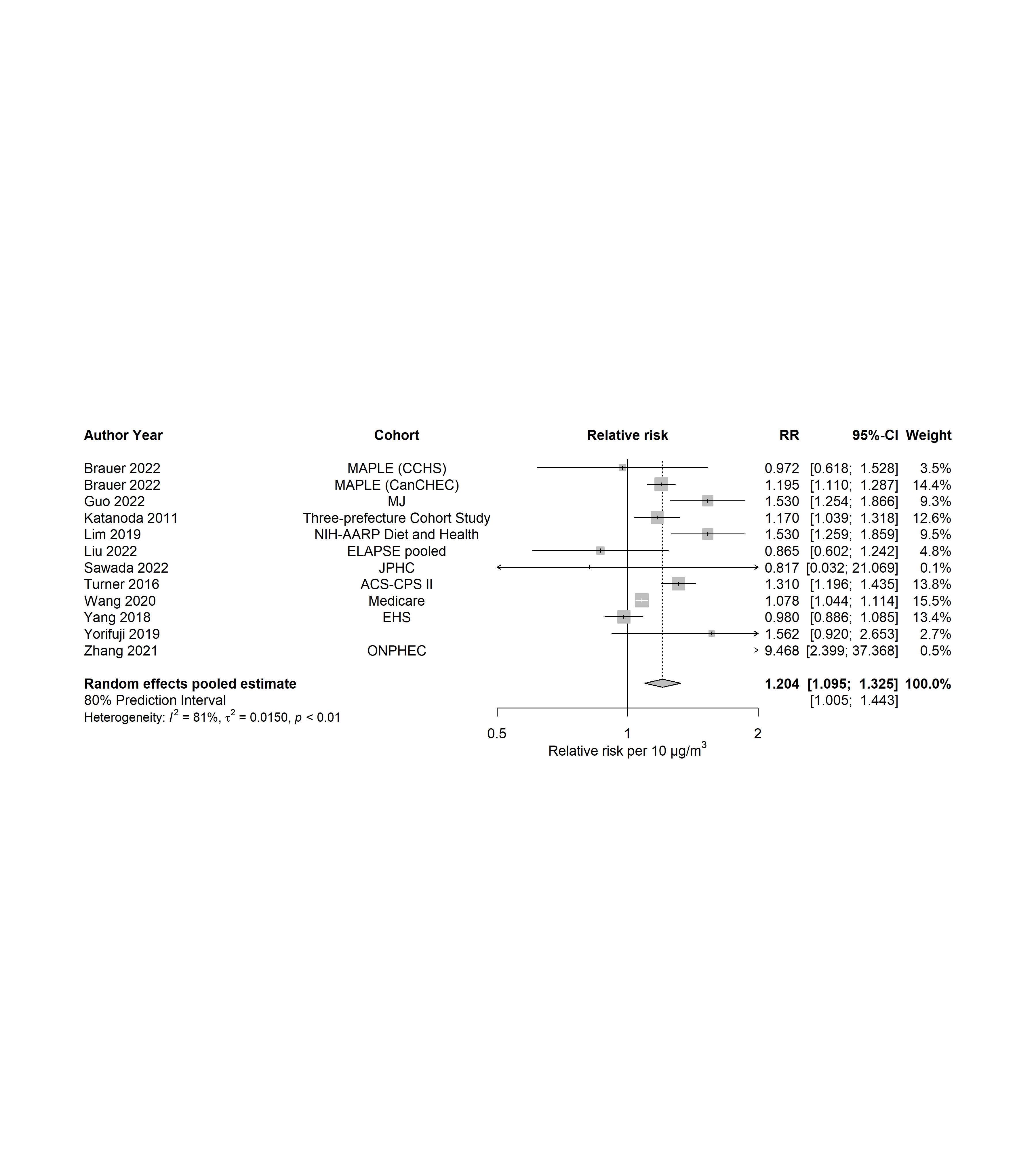


### **FIGURE S6** | Forest plot examining the association between PM_2.5_ and acute lower respiratory infection (ALRI) mortality (Global, 2023-2024).


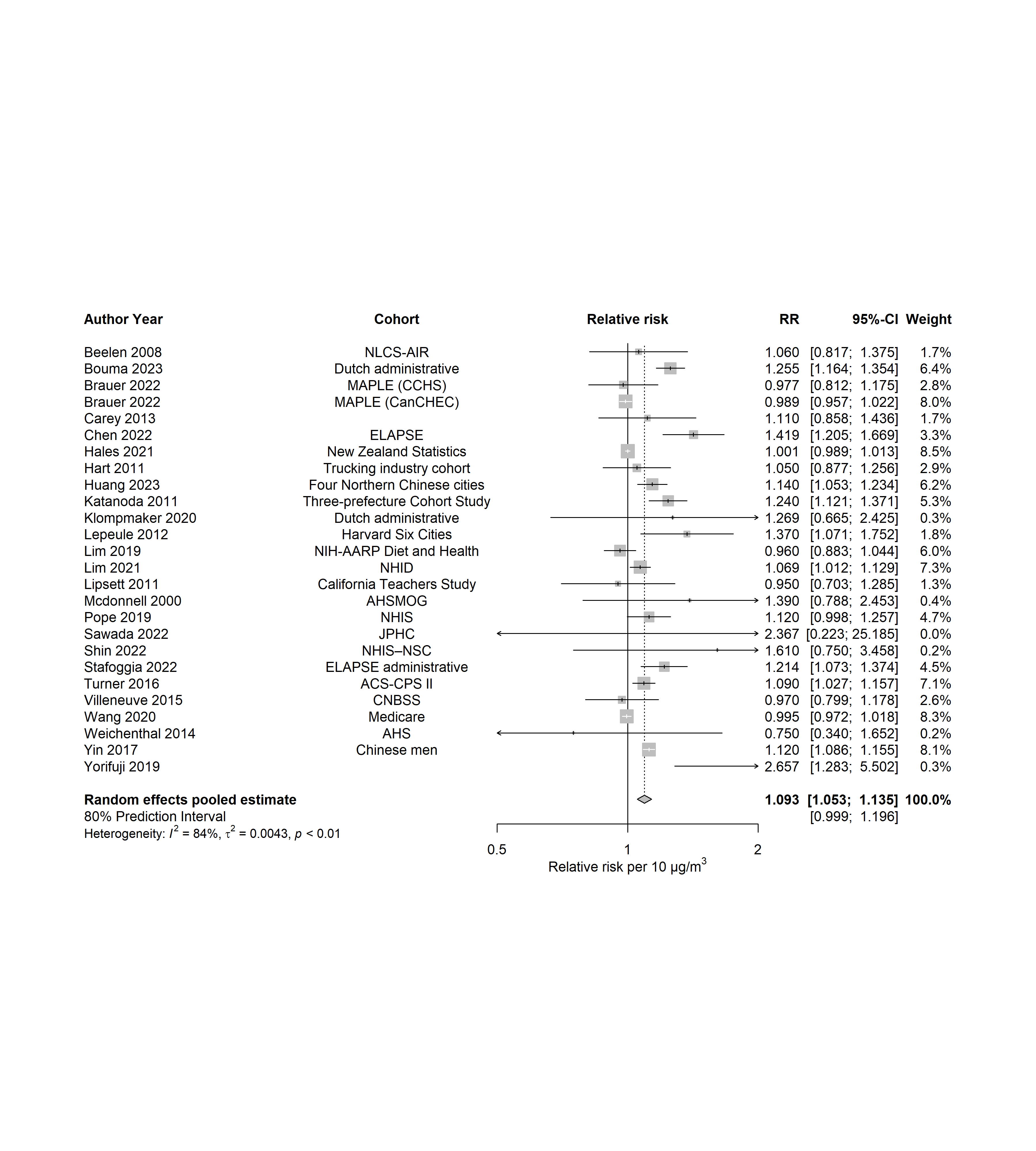


### **FIGURE S7** | Forest plot examining the association between PM_2.5_ and lung cancer mortality (Global, 2023-2024).


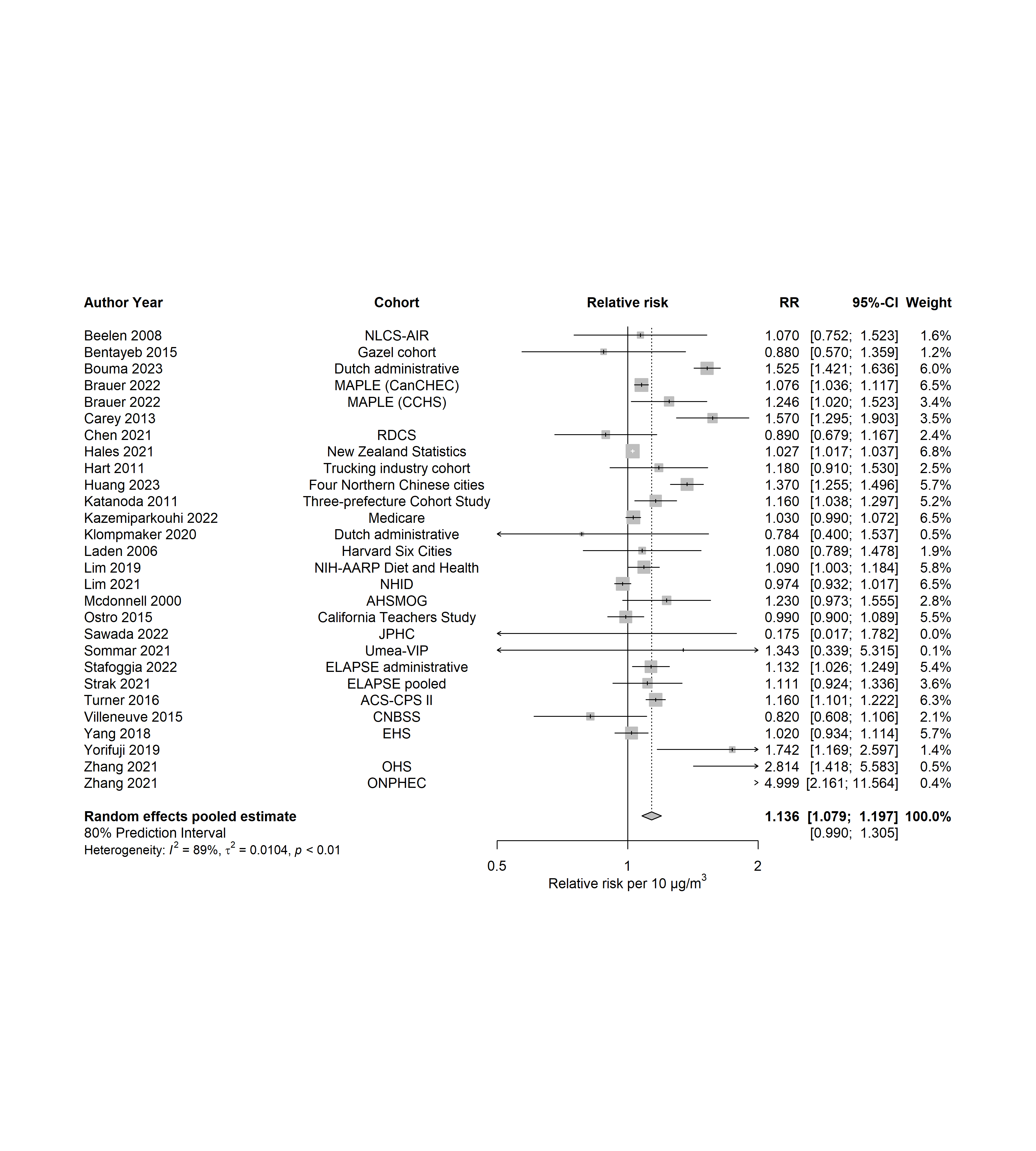


### **FIGURE S8** | Forest plot examining the association between PM_2.5_ and respiratory mortality (Global, 2023-2024).


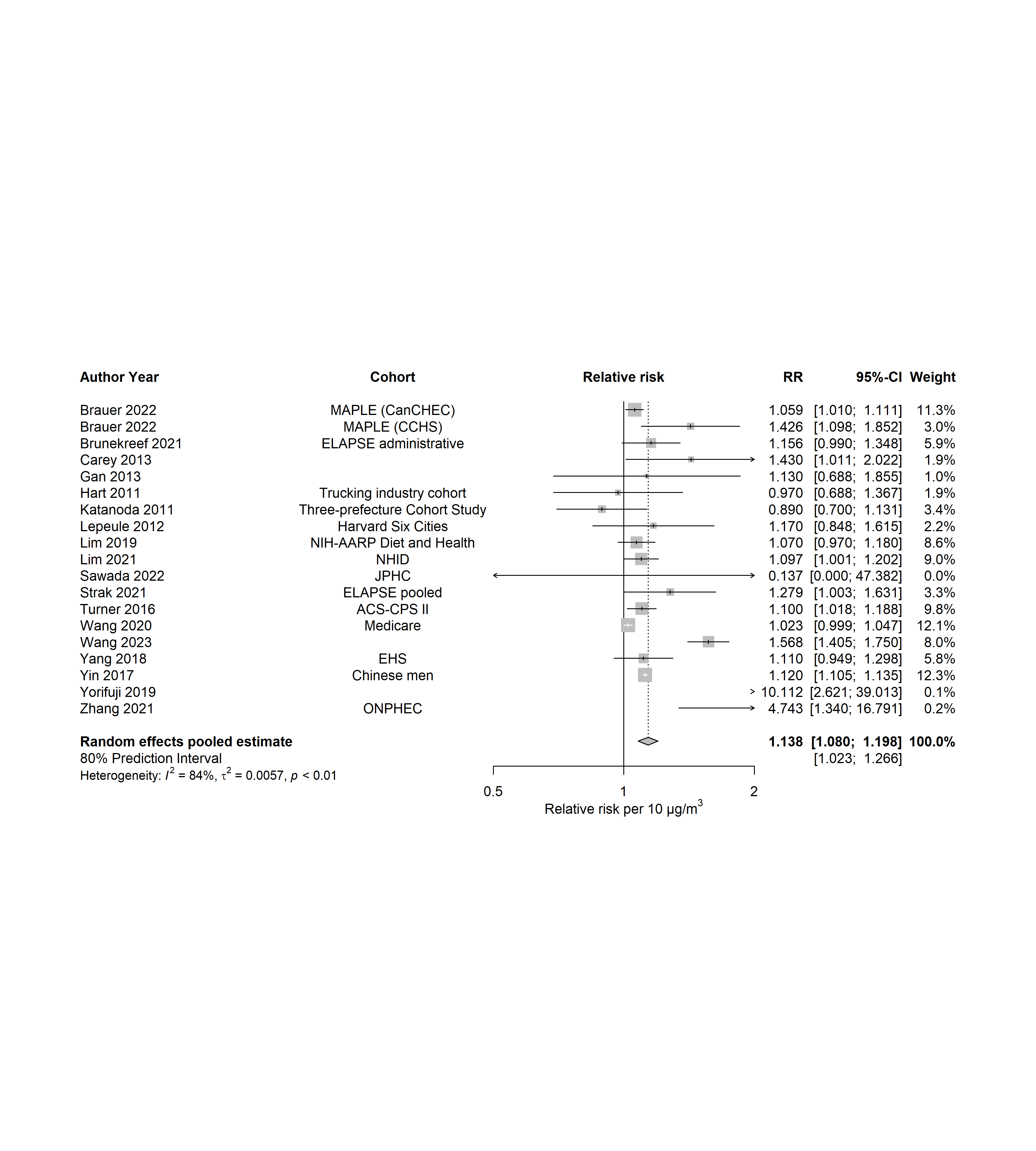


### **FIGURE S9** | Forest plot examining the association between PM_2.5_ and chronic obstructive pulmonary disease (COPD) mortality (Global, 2023-2024).


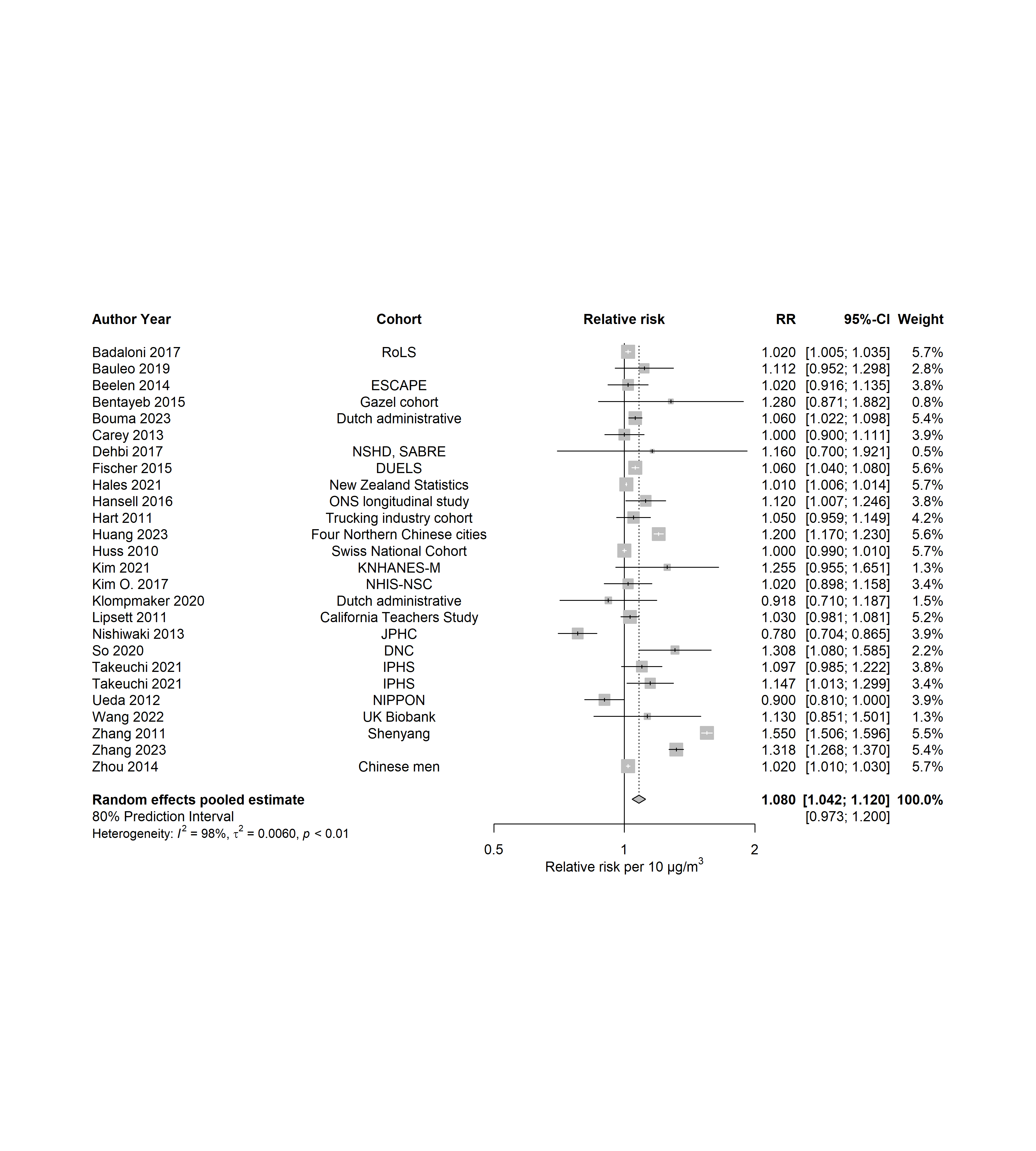


### **FIGURE S10** | Forest plot examining the association between PM_10_ and circulatory mortality (Global, 2023-2024).


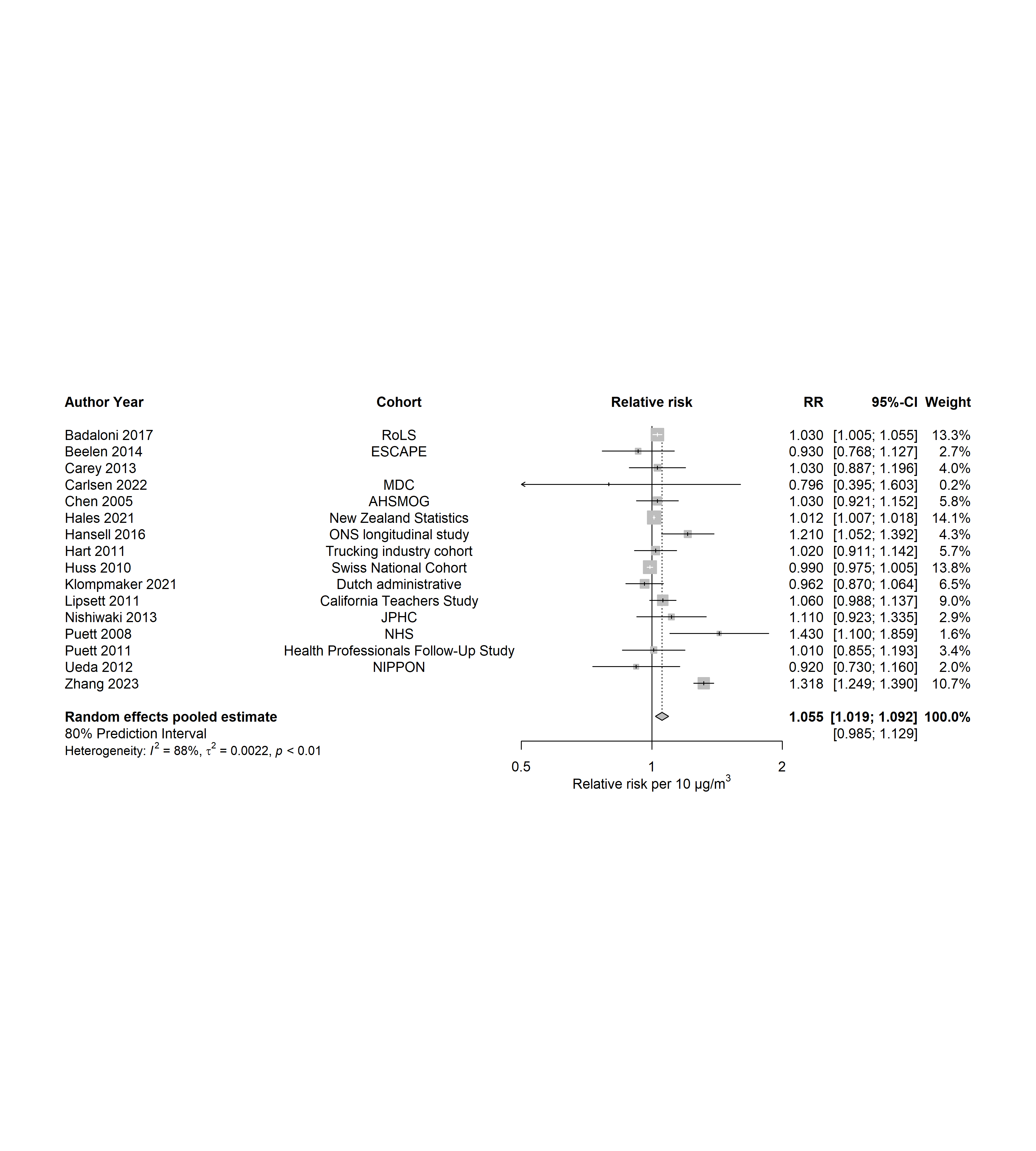


### **FIGURE S11** | Forest plot examining the association between PM_10_ and ischaemic heart disease (IHD) mortality (Global, 2023-2024).


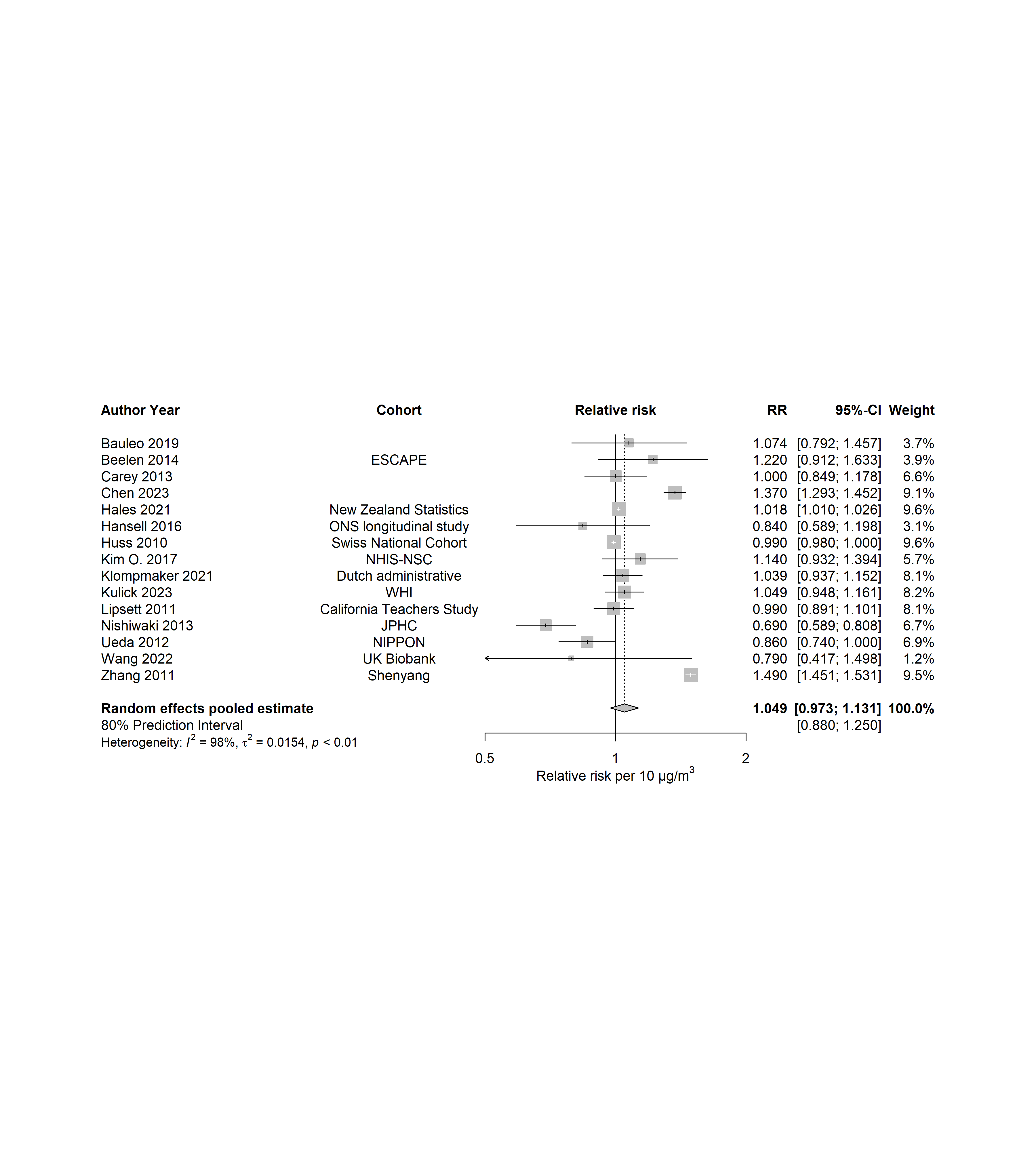


### **FIGURE S12** | Forest plot examining the association between PM_10_ and cerebrovascular mortality (Global, 2023-2024).


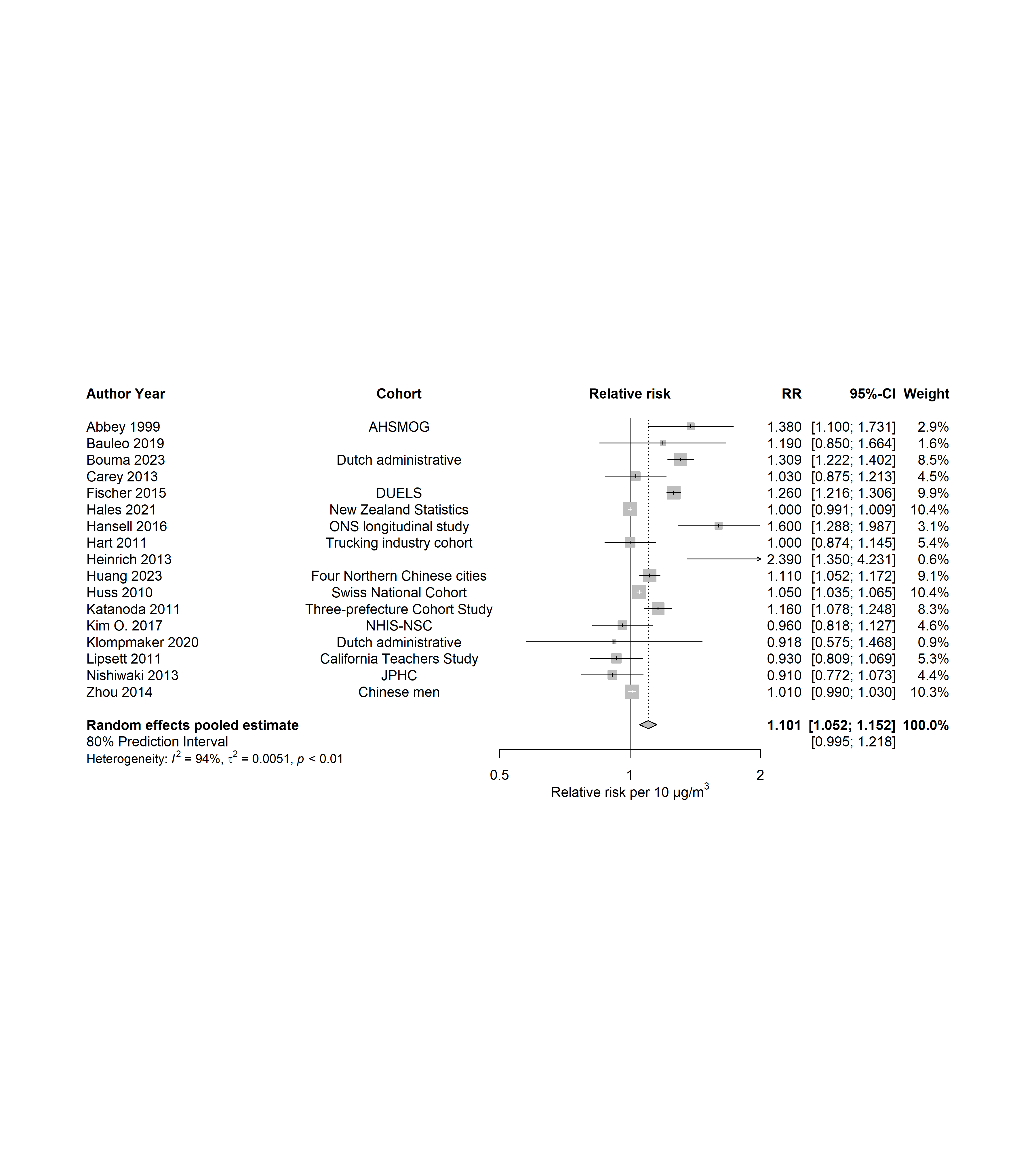


### **FIGURE S13** | Forest plot examining the association between PM_10_ and lung cancer mortality (Global, 2023-2024).


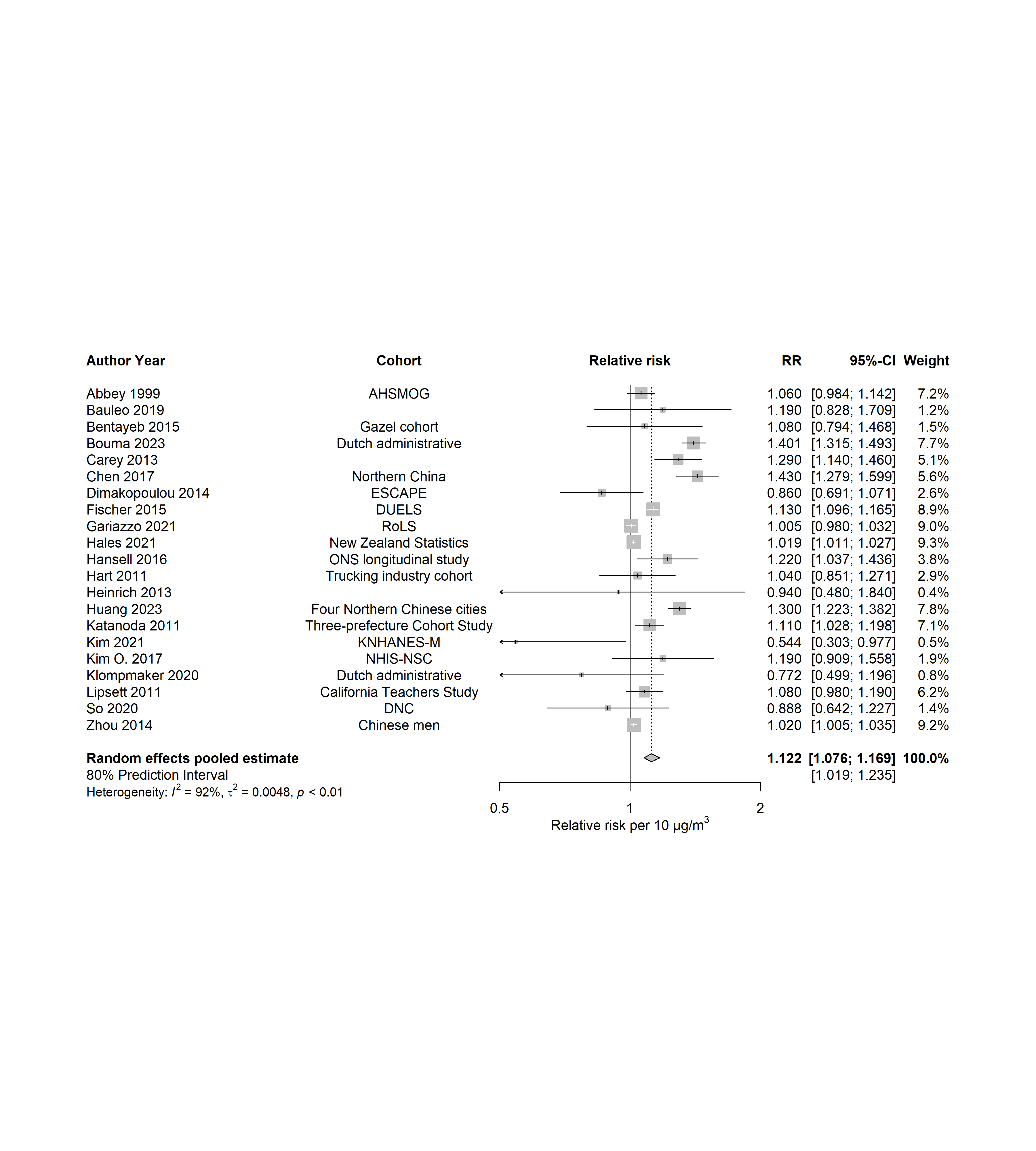


### **FIGURE S14** | Forest plot examining the association between PM_10_ and respiratory mortality (Global, 2023-2024).


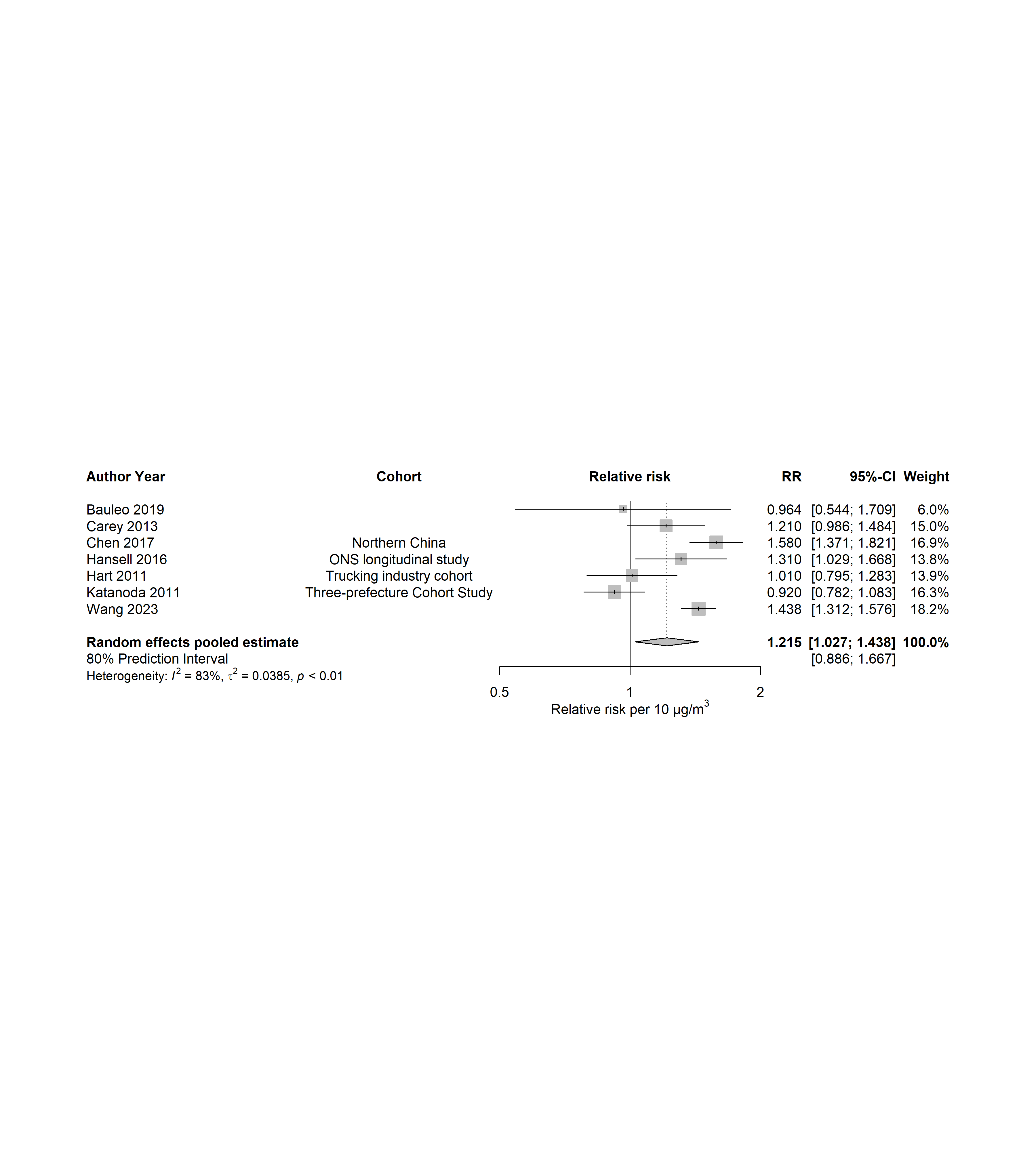


### **FIGURE S15** | Forest plot examining the association between PM_10_ and chronic obstructive pulmonary disease (COPD) mortality (Global, 2023-2024).


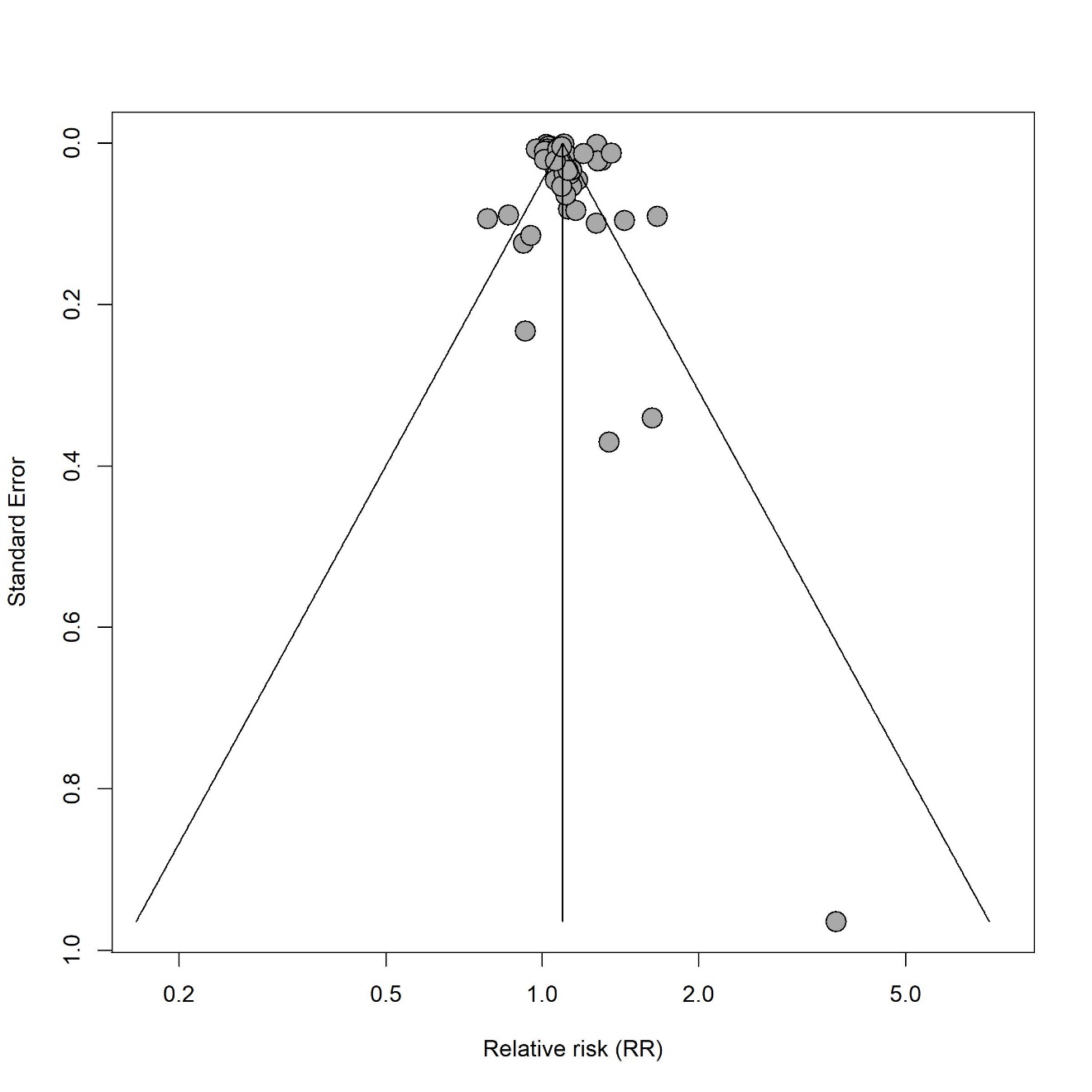


### **FIGURE S16** | Funnel plot exploring potential publication bias for PM_2.5_ and all-cause mortality (Global, 2023-2024).


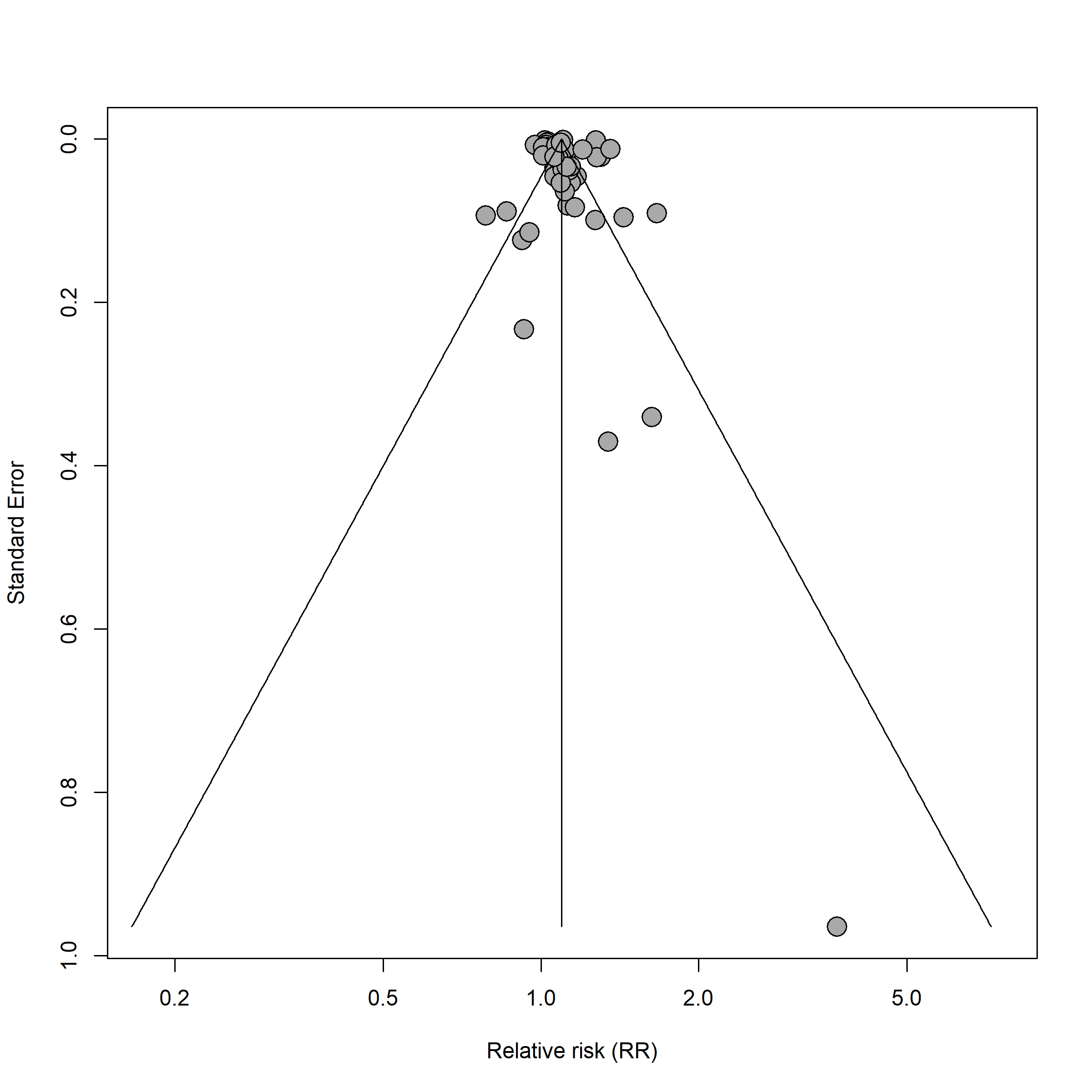


### **FIGURE S17** | Funnel plot exploring potential publication bias for PM_10_ and all-cause mortality (Global, 2023-2024).


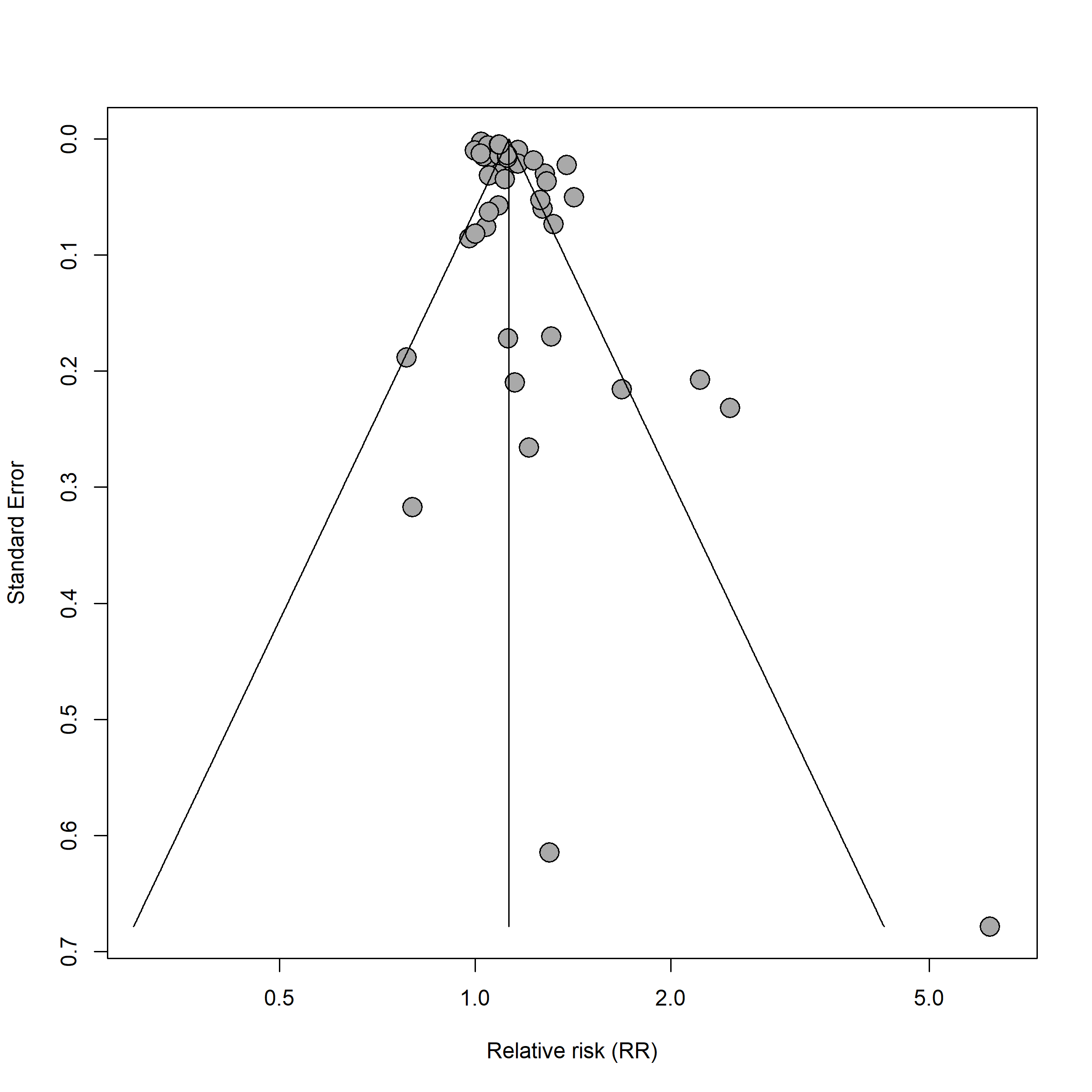


### **FIGURE S18** | Funnel plot exploring potential publication bias for PM_2.5_ and circulatory mortality (Global, 2023-2024).


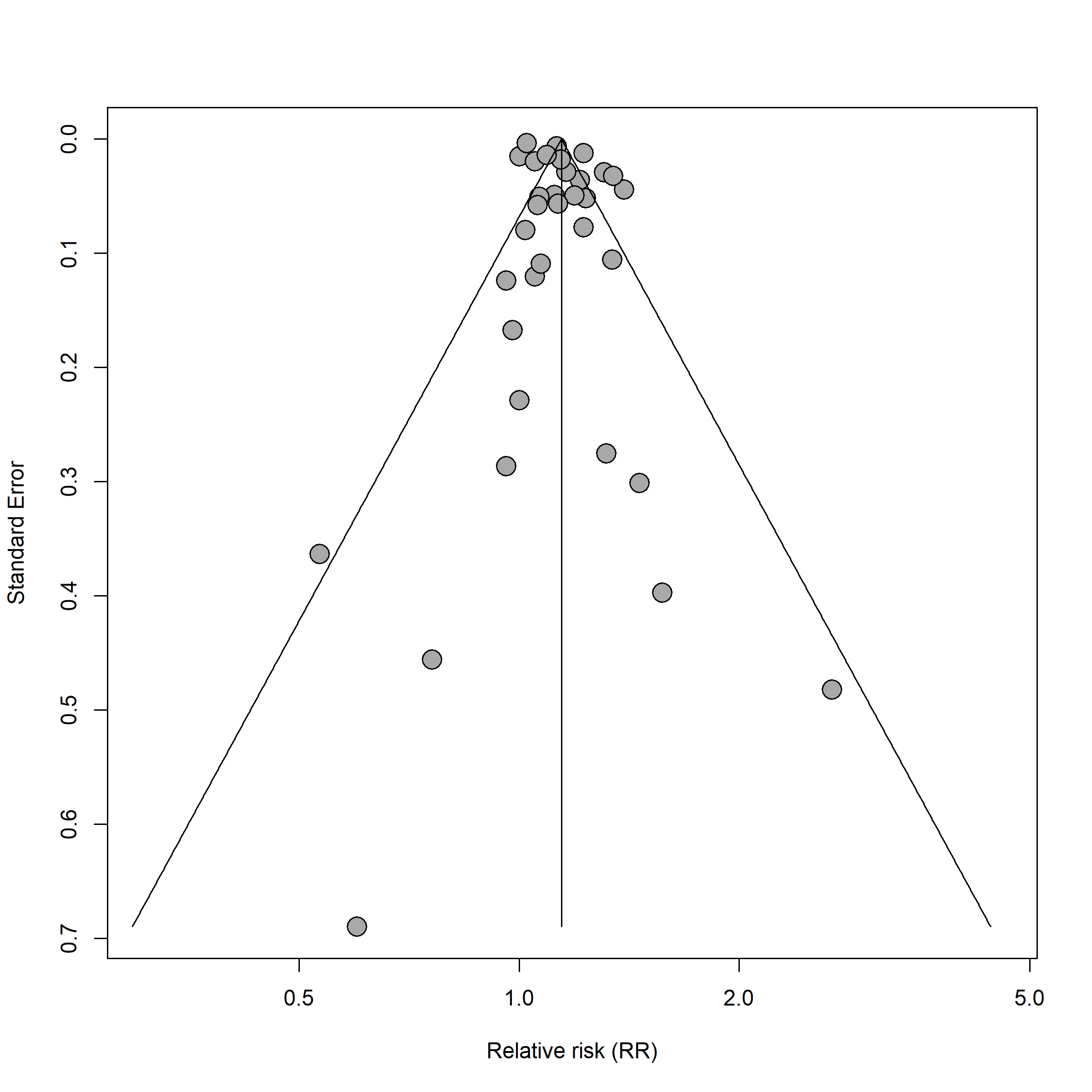


### **FIGURE S19** | Funnel plot exploring potential publication bias for PM_2.5_ and ischaemic heart disease (IHD) mortality (Global, 2023-2024).


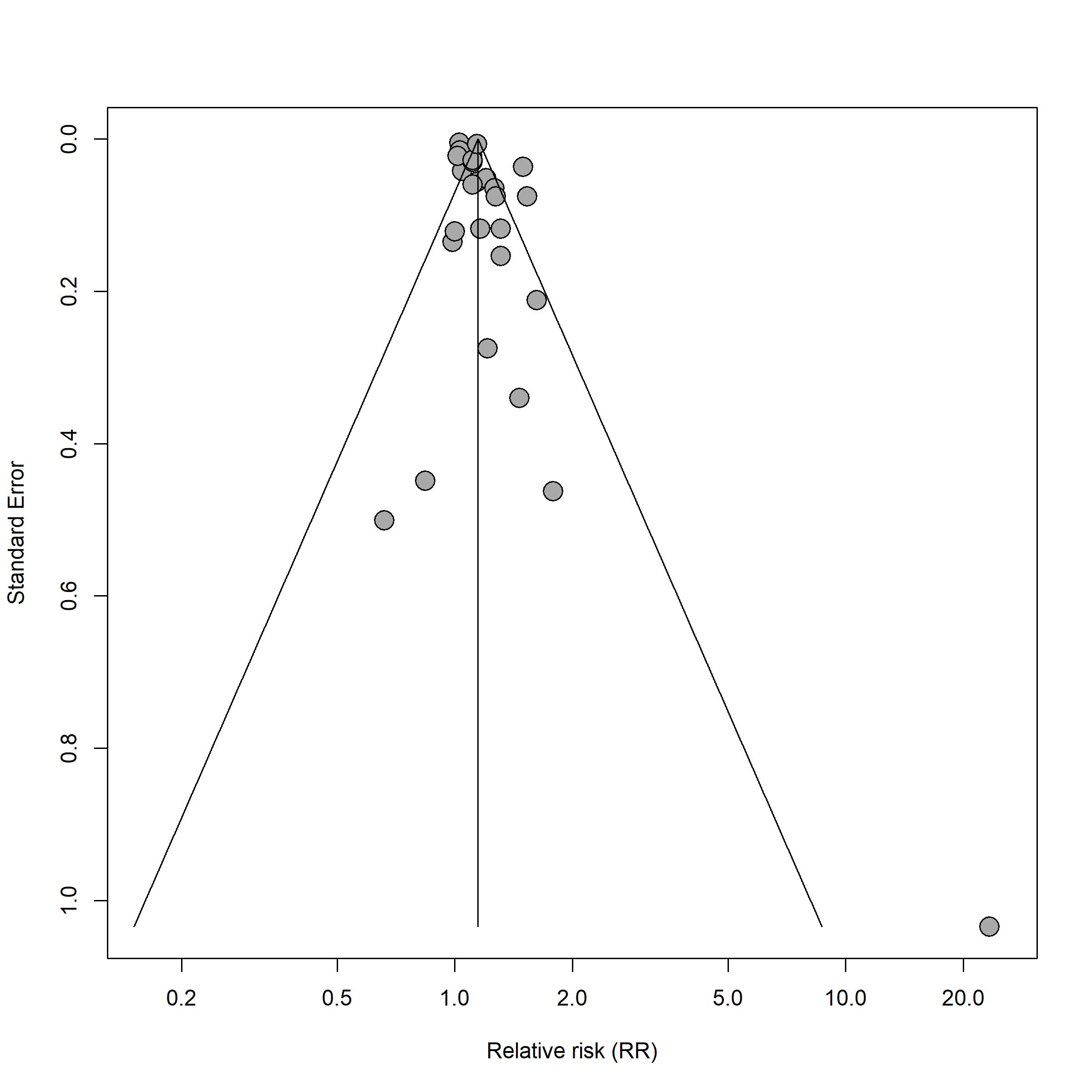


### **FIGURE S20** | Funnel plot exploring potential publication bias for PM_2.5_ and cerebrovascular mortality (Global, 2023-2024).


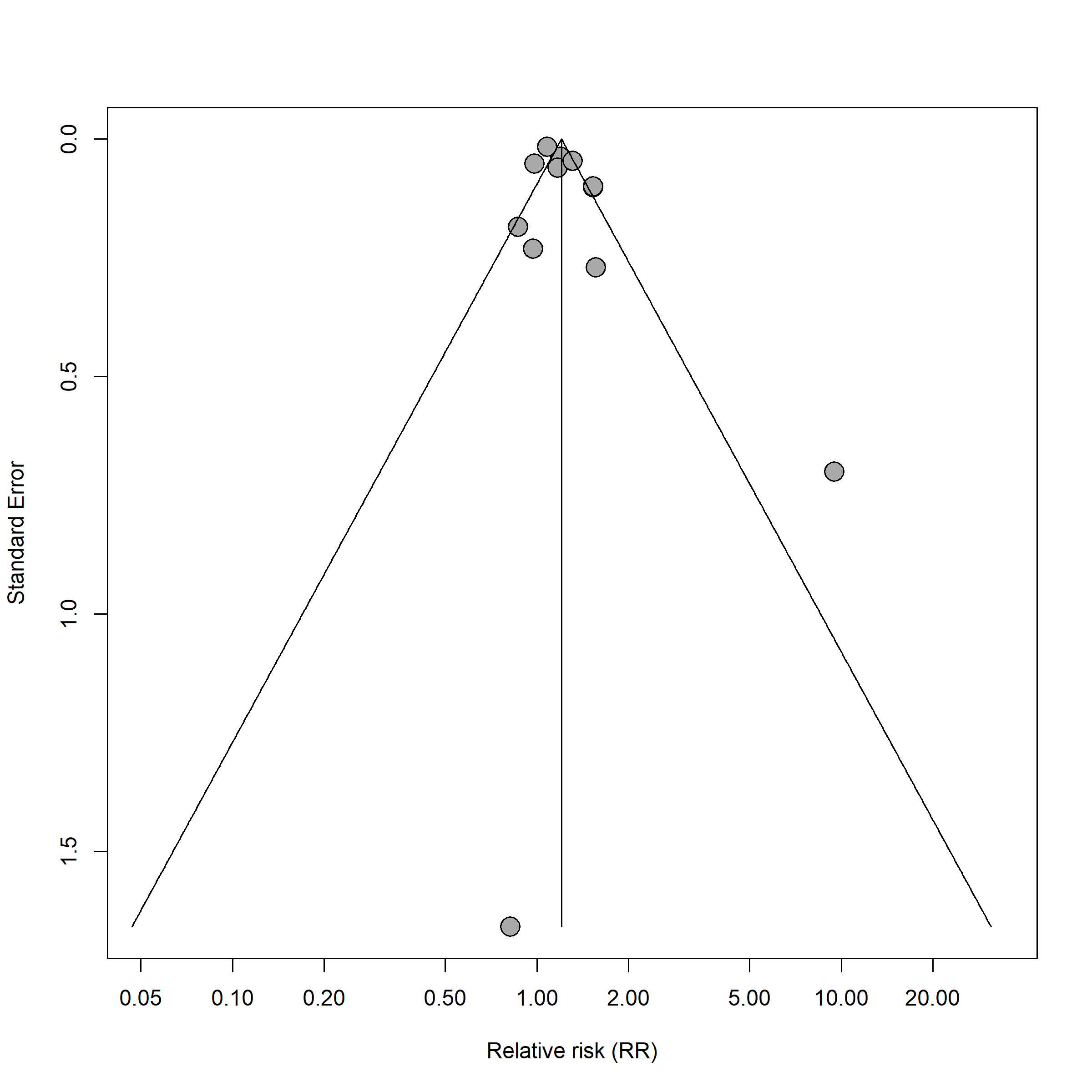


### **FIGURE S21** | Funnel plot exploring potential publication bias for PM_2.5_ and acute lower respiratory infection (ALRI) mortality (Global, 2023-2024).


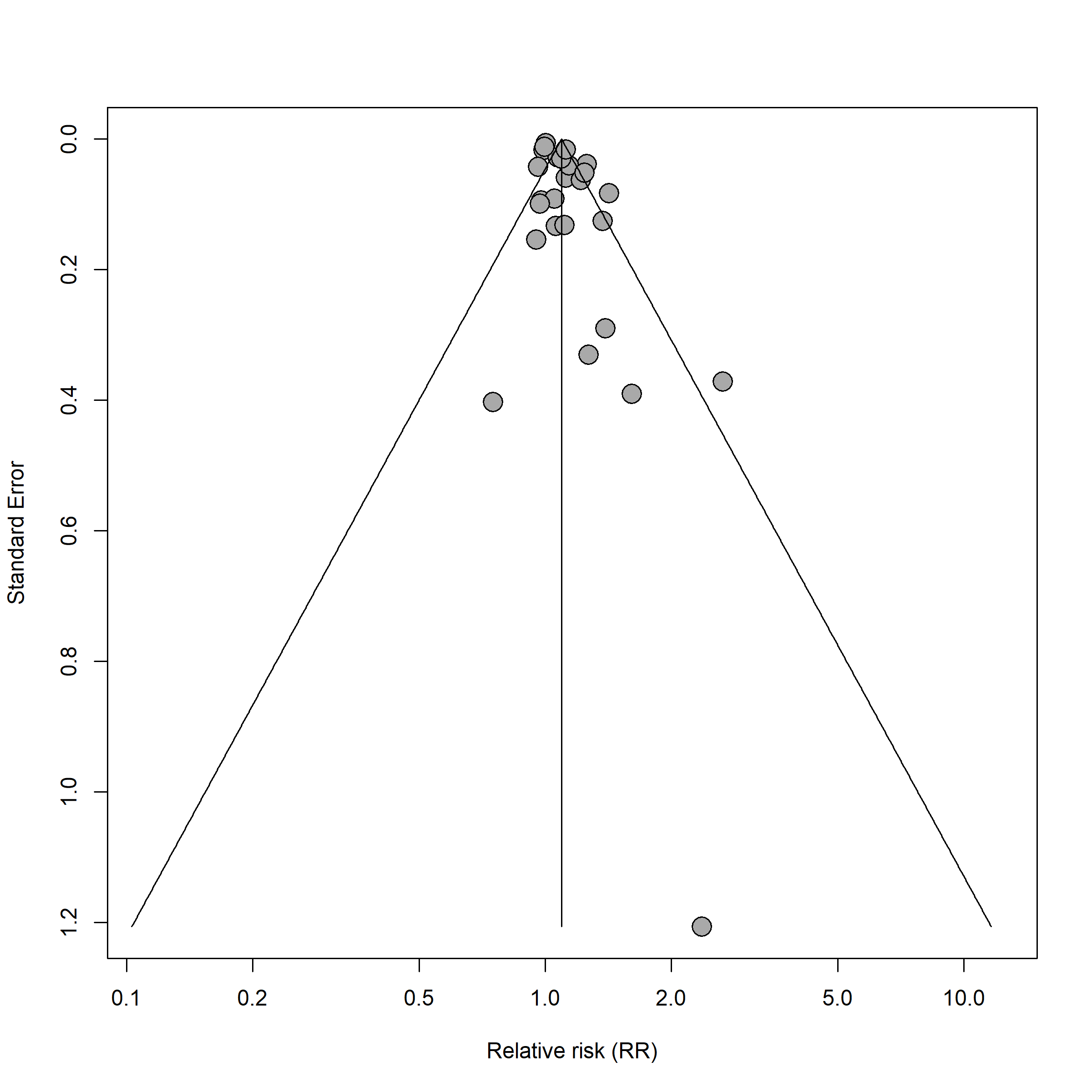


### **FIGURE S22** | Funnel plot exploring potential publication bias for PM_2.5_ and lung cancer mortality (Global, 2023-2024).


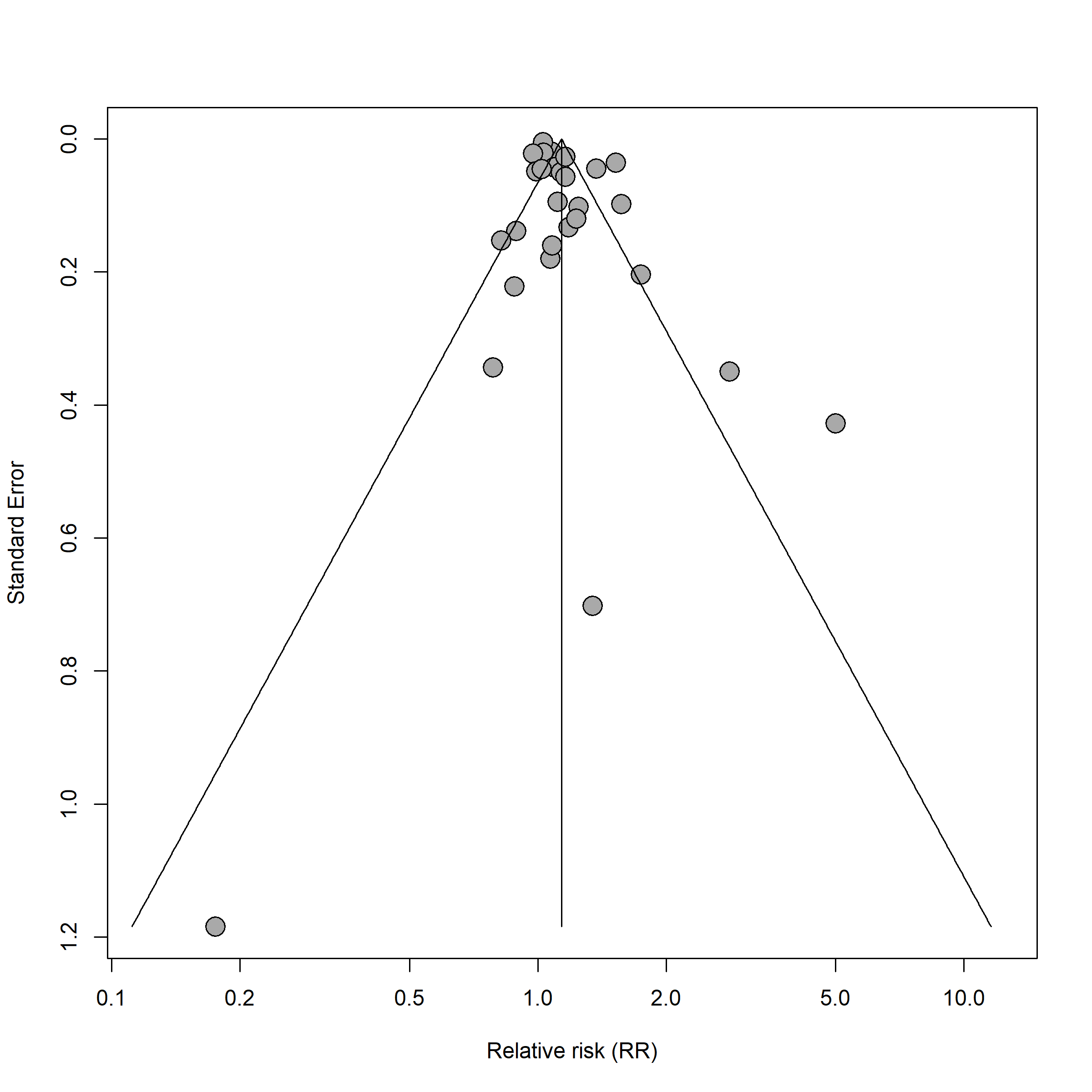


### **FIGURE S23** | Funnel plot exploring potential publication bias for PM_2.5_ and respiratory mortality (Global, 2023-2024).


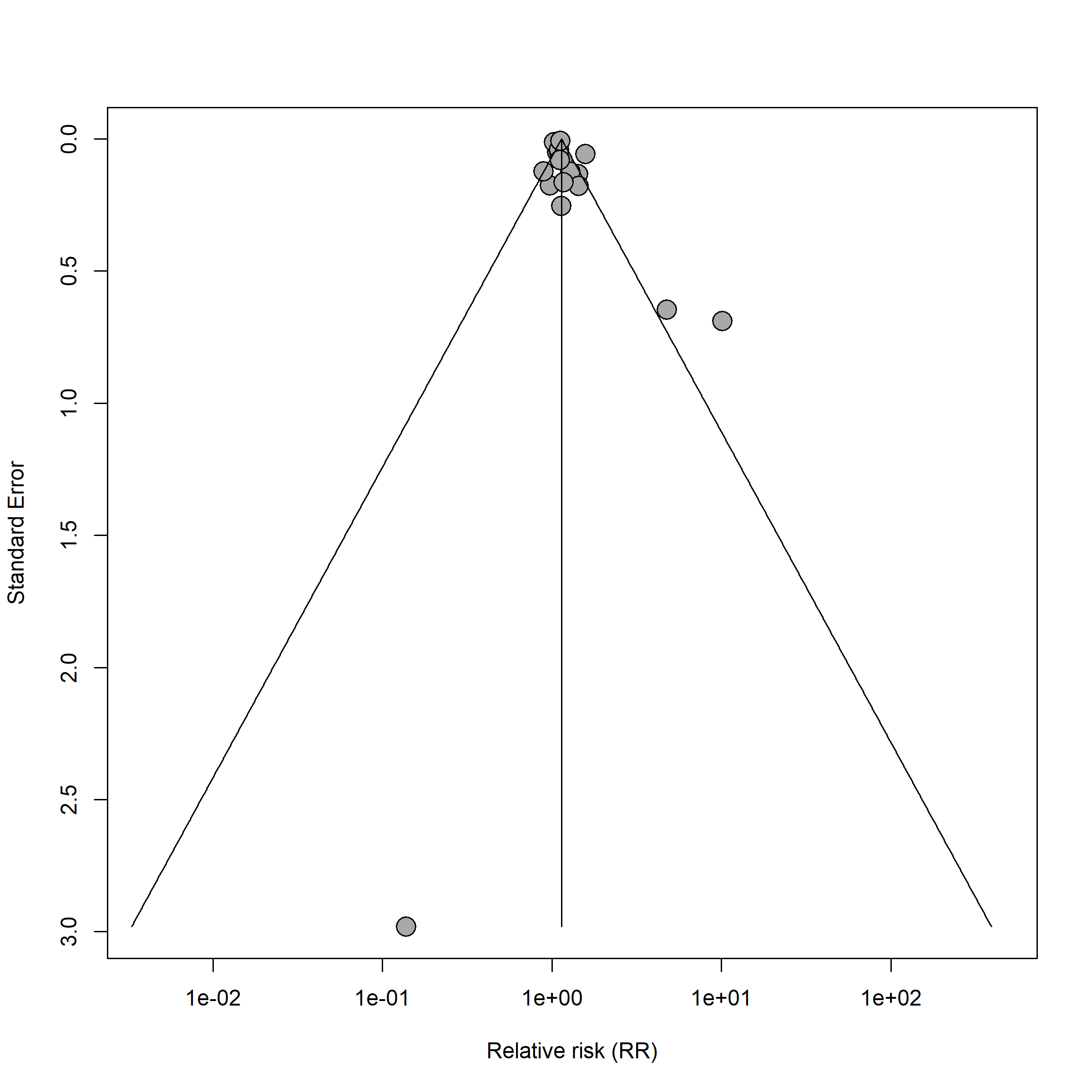


### **FIGURE S24** | Funnel plot exploring potential publication bias for PM_2.5_ and chronic obstructive pulmonary disease (COPD) mortality (Global, 2023-2024).


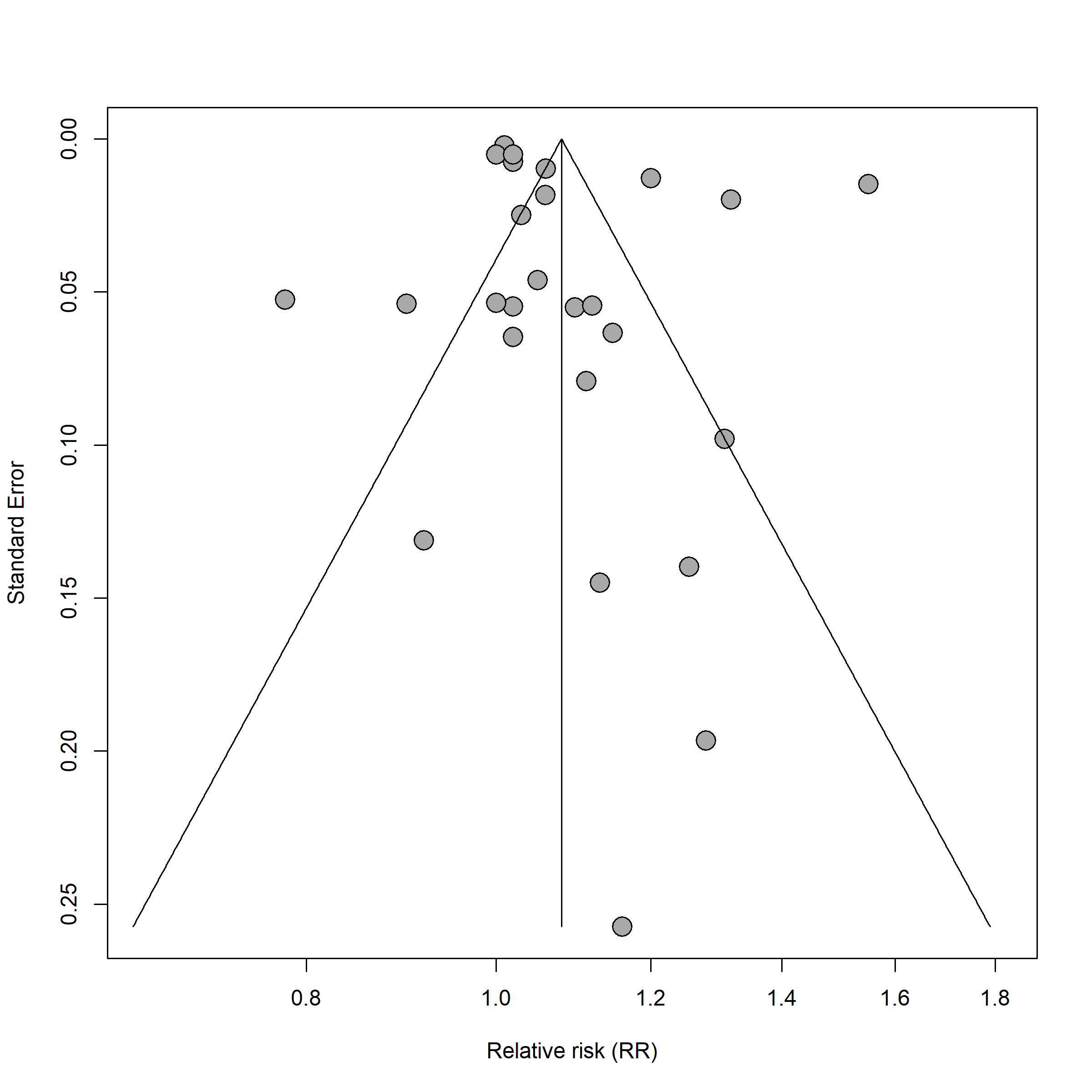


### **FIGURE S25** | Funnel plot exploring potential publication bias for PM_10_ and circulatory mortality (Global, 2023-2024).

**
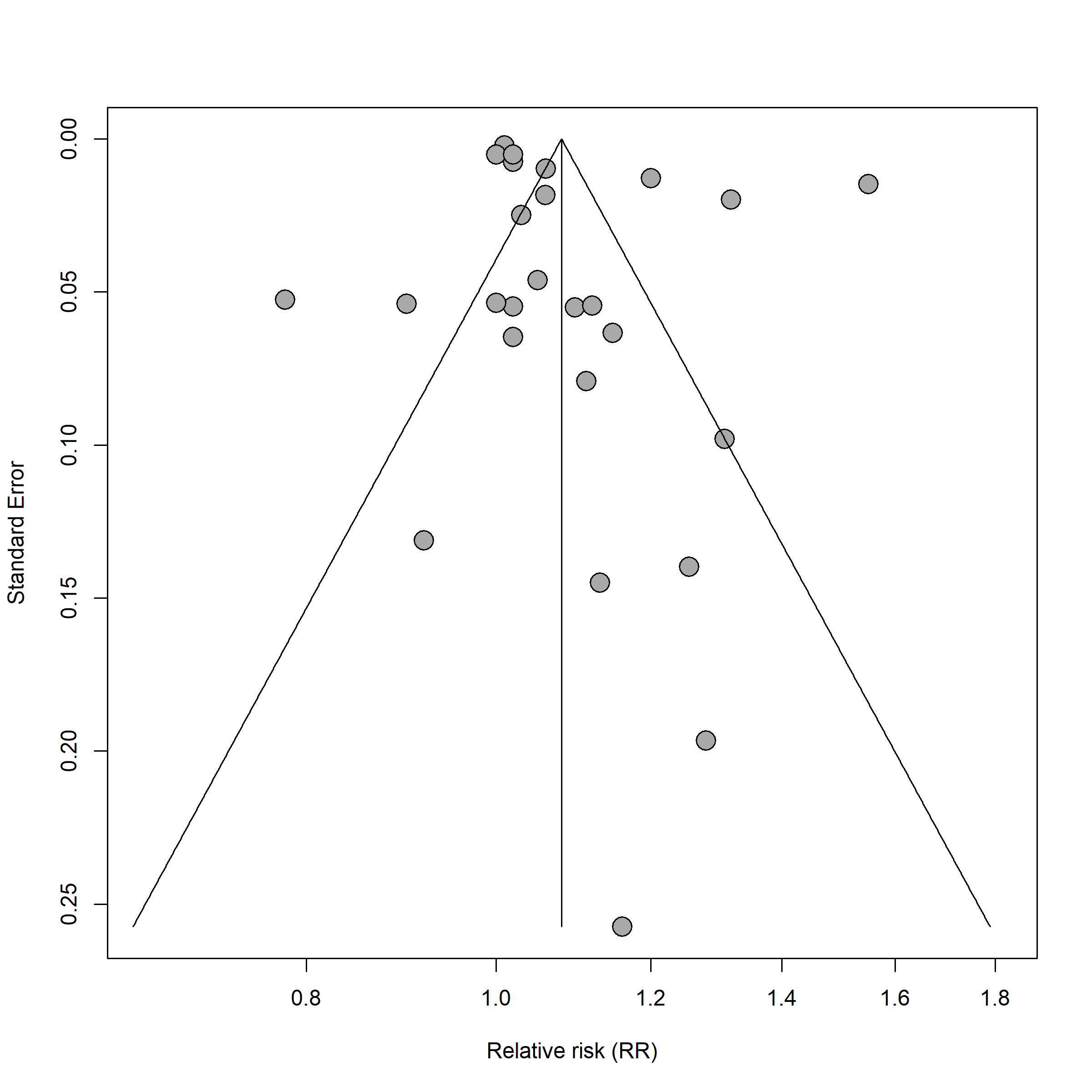
**

### **FIGURE S26** | Funnel plot exploring potential publication bias for PM_10_ and ischaemic heart disease (IHD) mortality (Global, 2023-2024).


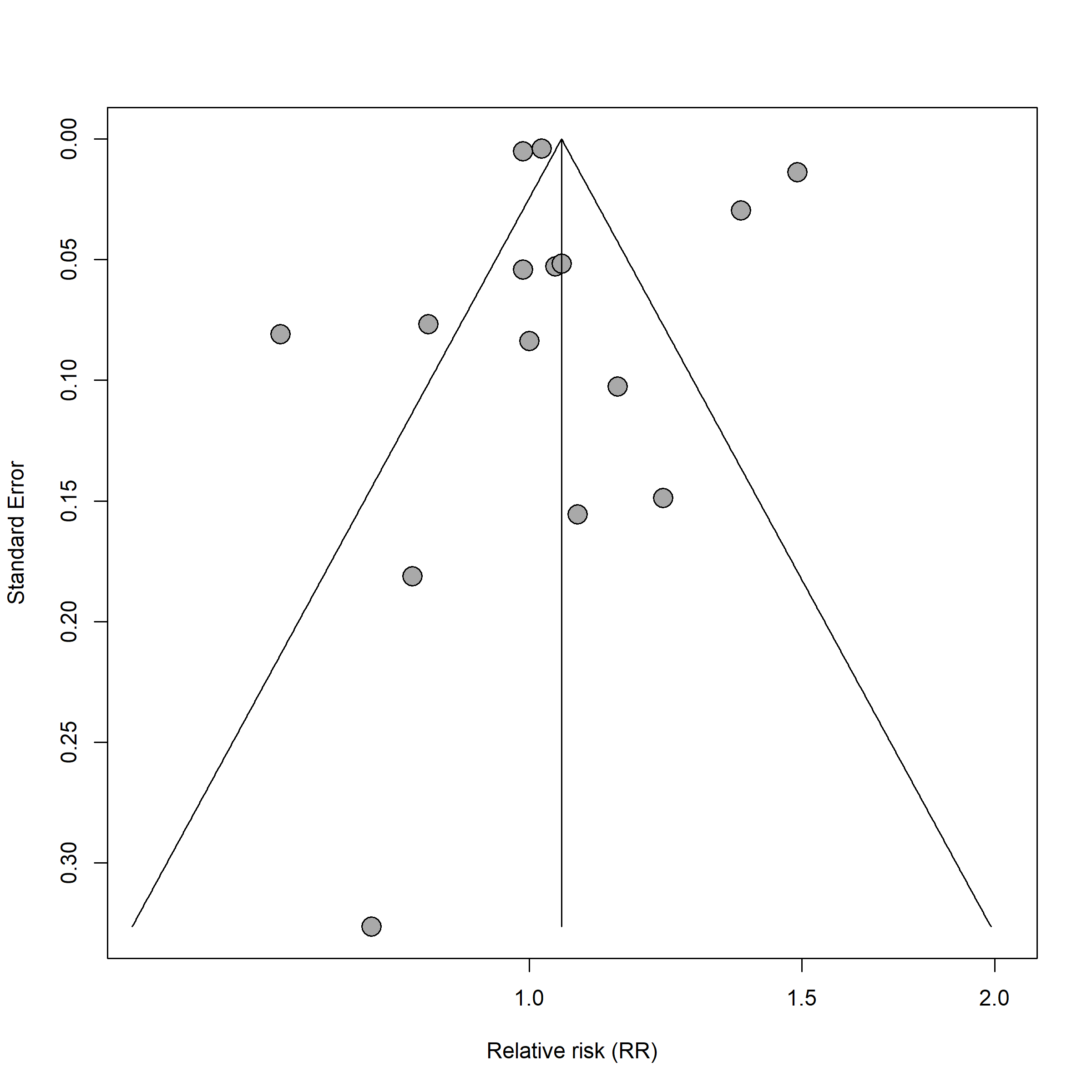


### **FIGURE S27** | Funnel plot exploring potential publication bias for PM_10_ and cerebrovascular mortality (Global, 2023-2024).


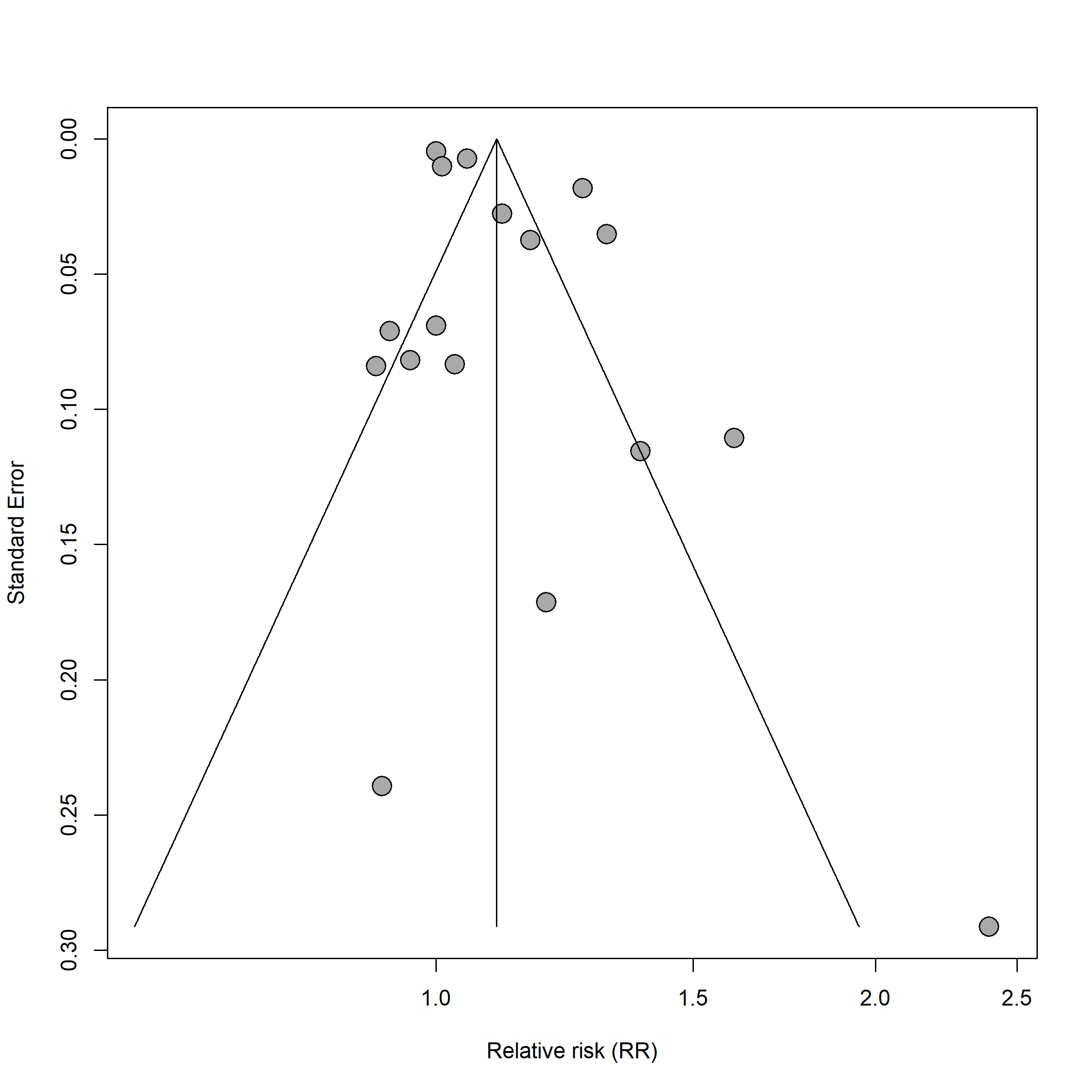


### **FIGURE S28** | Funnel plot exploring potential publication bias for PM_10_ and lung cancer mortality (Global, 2023-2024).


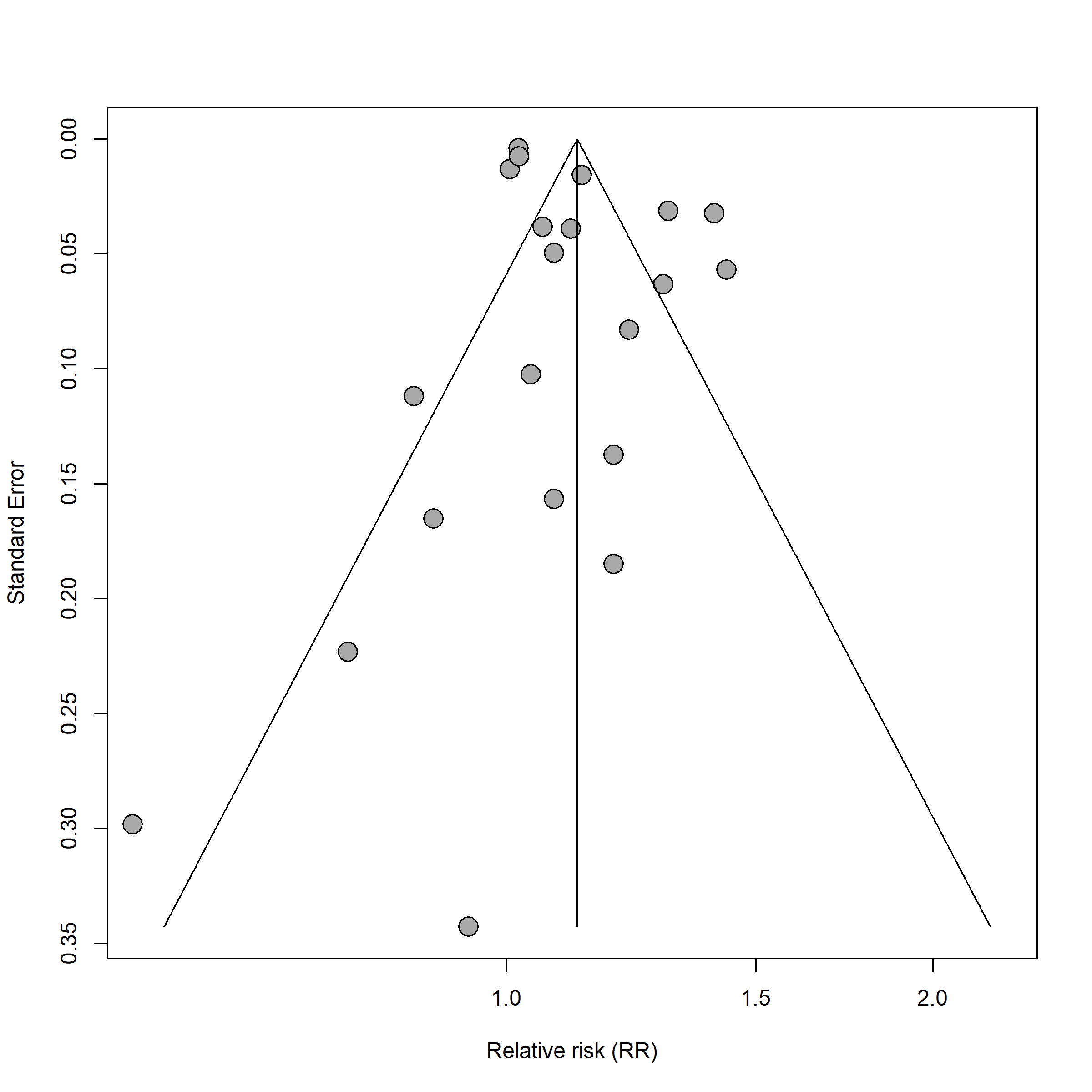


### **FIGURE S29** | Funnel plot exploring potential publication bias for PM_10_ and respiratory mortality (Global, 2023-2024).


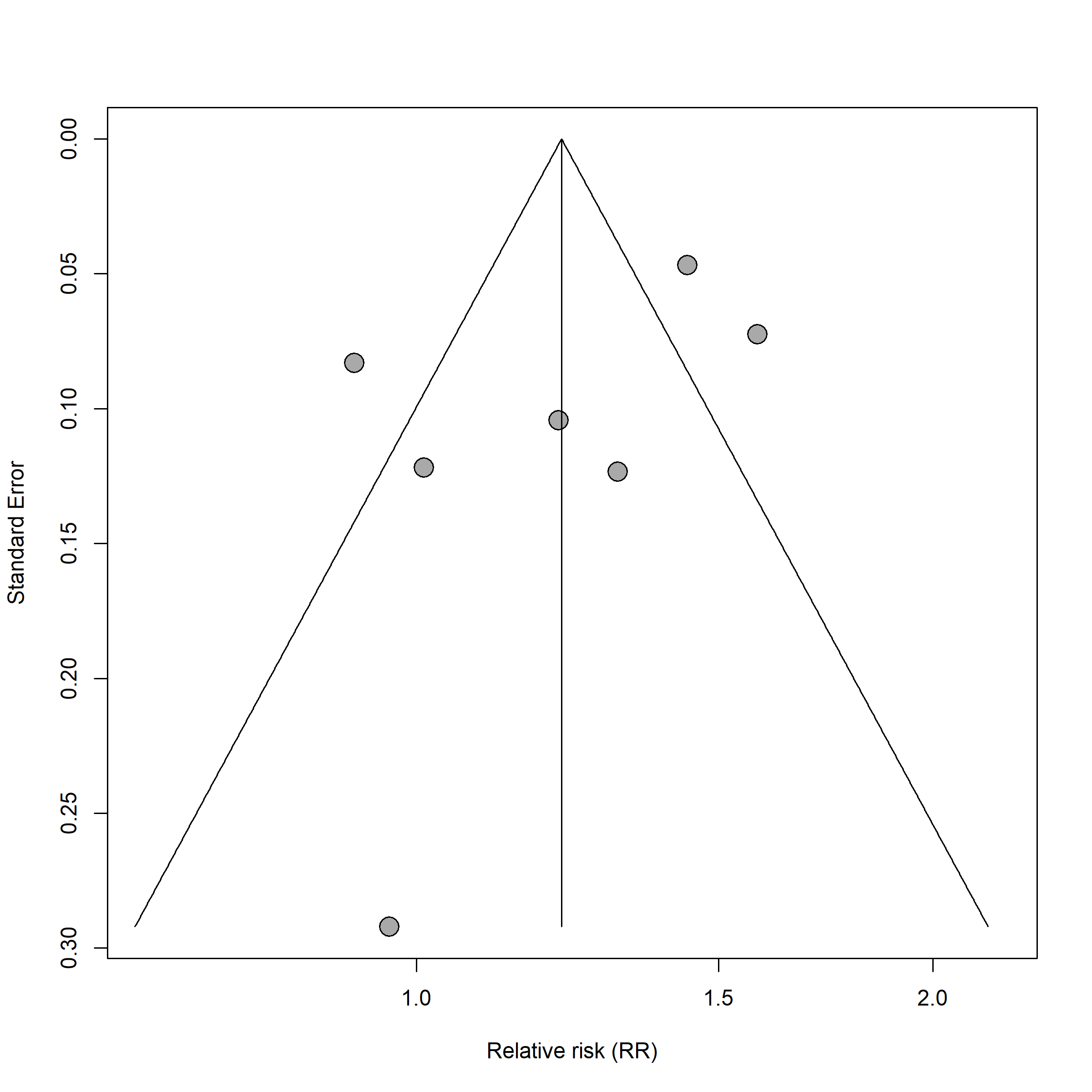


### **FIGURE S30** | Funnel plot exploring potential publication bias for PM_10_ and chronic obstructive pulmonary disease (COPD) mortality (Global, 2023-2024).


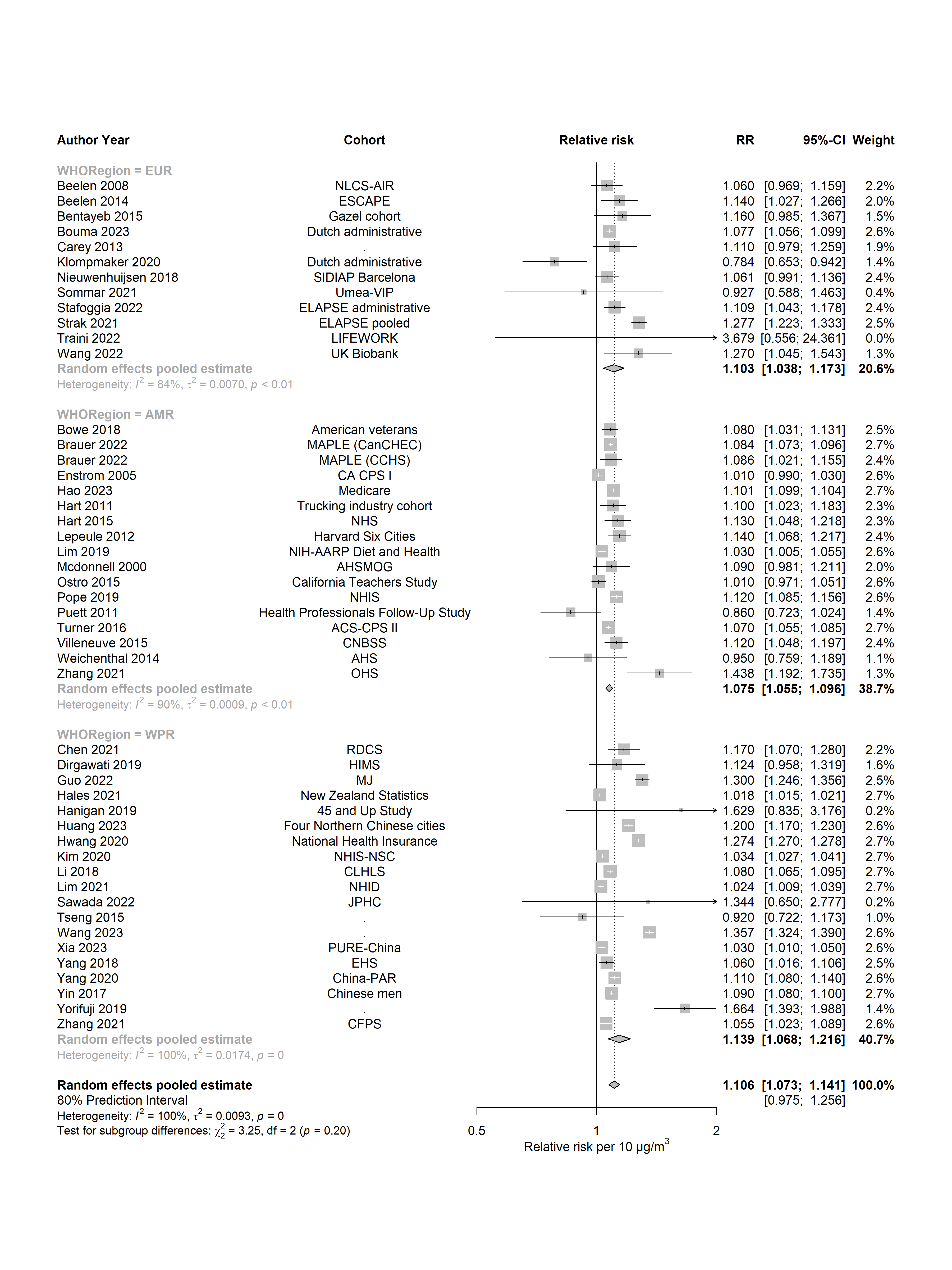


### **FIGURE S31** | Forest plot of the association between PM_2.5_ and all-cause mortality. Subgroup analysis by WHO region: European Region (EUR), Region of the Americas (AMR), Western Pacific Region (WPR) (Global, 2023-2024).


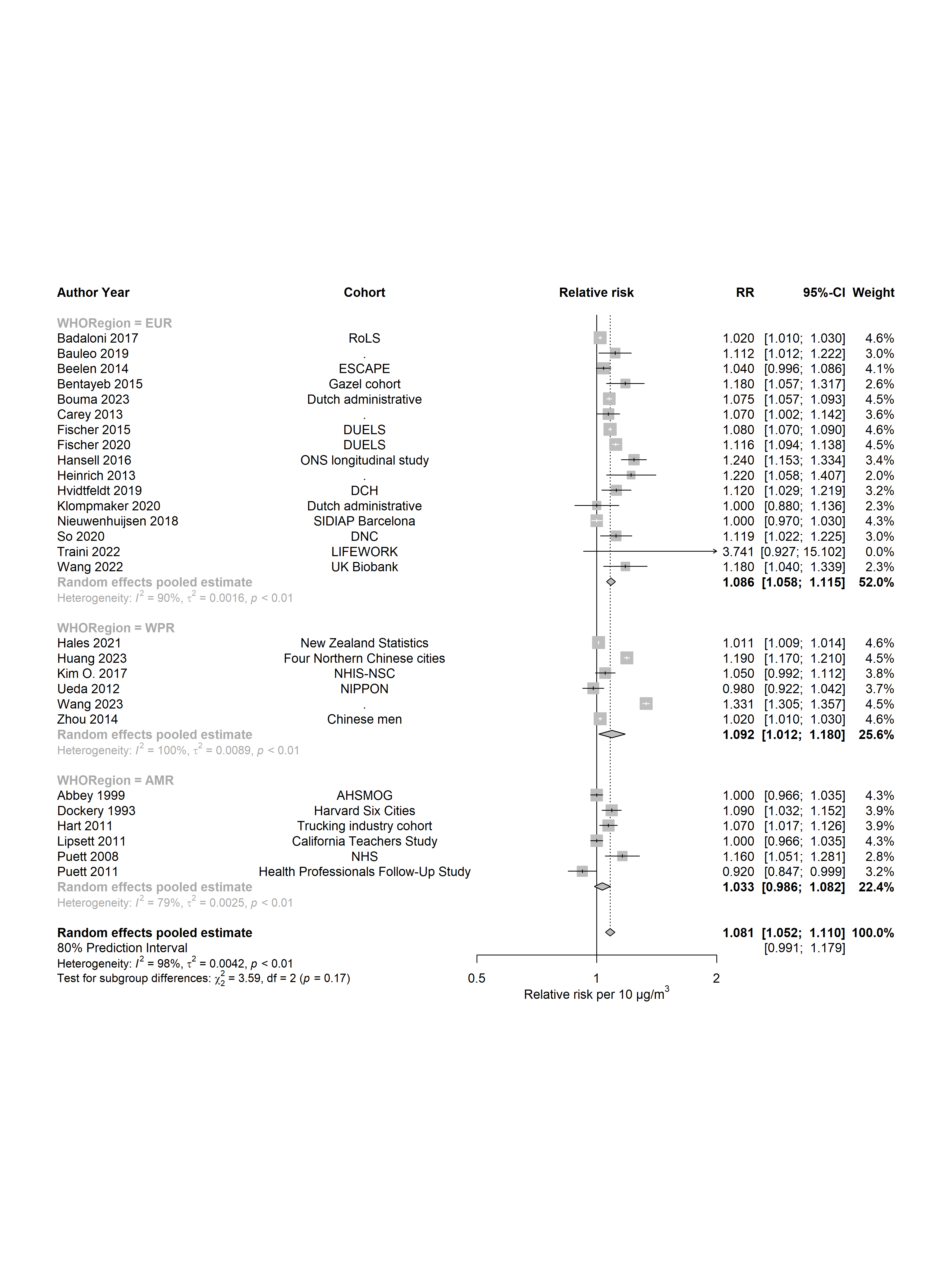


### **FIGURE S32** | Forest plot of the association between PM_10_ and all-cause mortality. Subgroup analysis by WHO region: European Region (EUR), Region of the Americas (AMR), Western Pacific Region (WPR) (Global, 2023-2024).


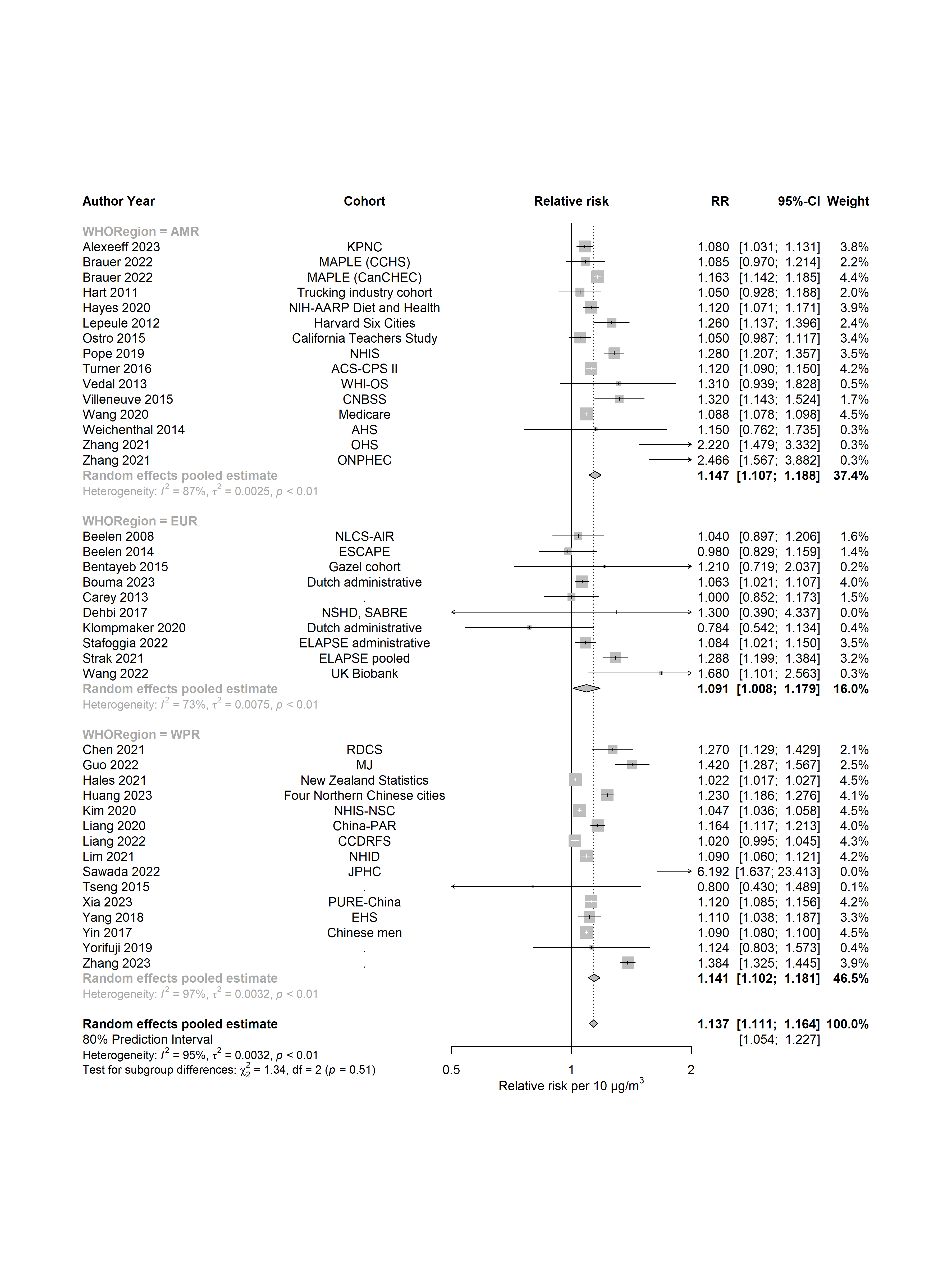


### **FIGURE S33** | Forest plot of the association between PM_2.5_ and circulatory mortality. Subgroup analysis by WHO region: European Region (EUR), Region of the Americas (AMR), Western Pacific Region (WPR) (Global, 2023-2024).


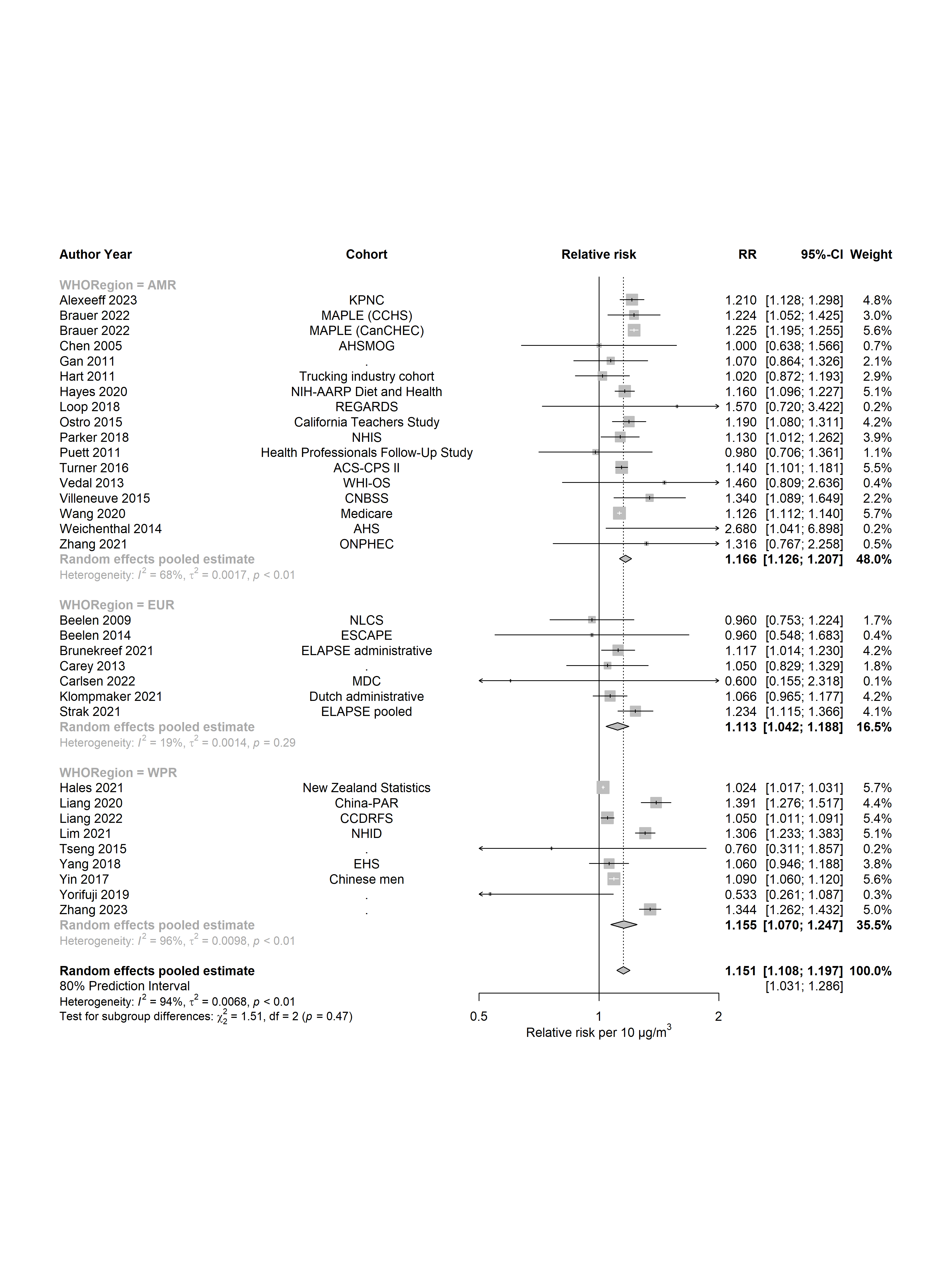


### **FIGURE S34** | Forest plot of the association between PM_2.5_ and ischaemic heart disease (IHD) mortality. Subgroup analysis by WHO region: European Region (EUR), Region of the Americas (AMR), Western Pacific Region (WPR) (Global, 2023-2024).


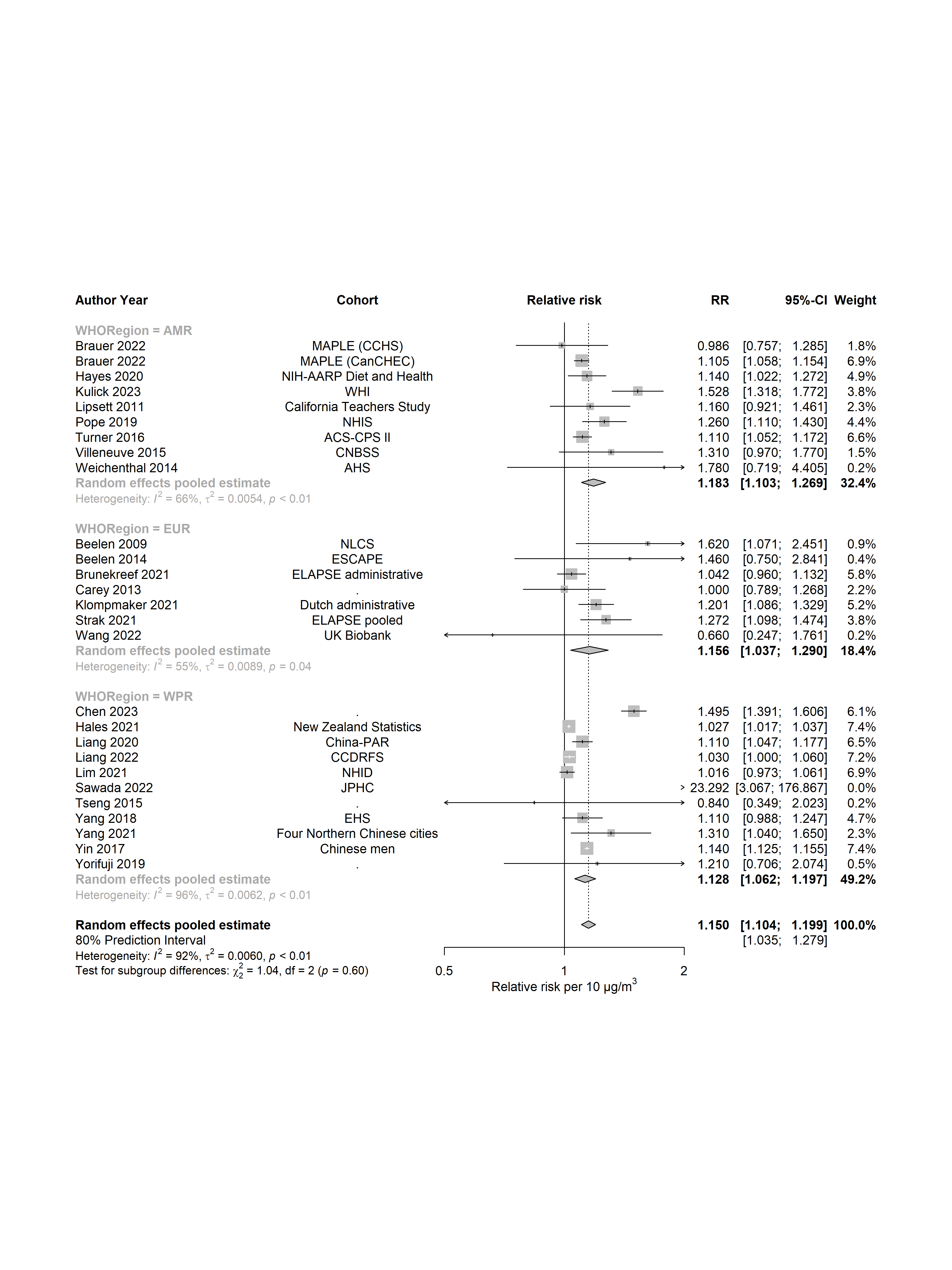


### **FIGURE S35** | Forest plot of the association between PM_2.5_ and cerebrovascular mortality. Subgroup analysis by WHO region: European Region (EUR), Region of the Americas (AMR), Western Pacific Region (WPR) (Global, 2023-2024).


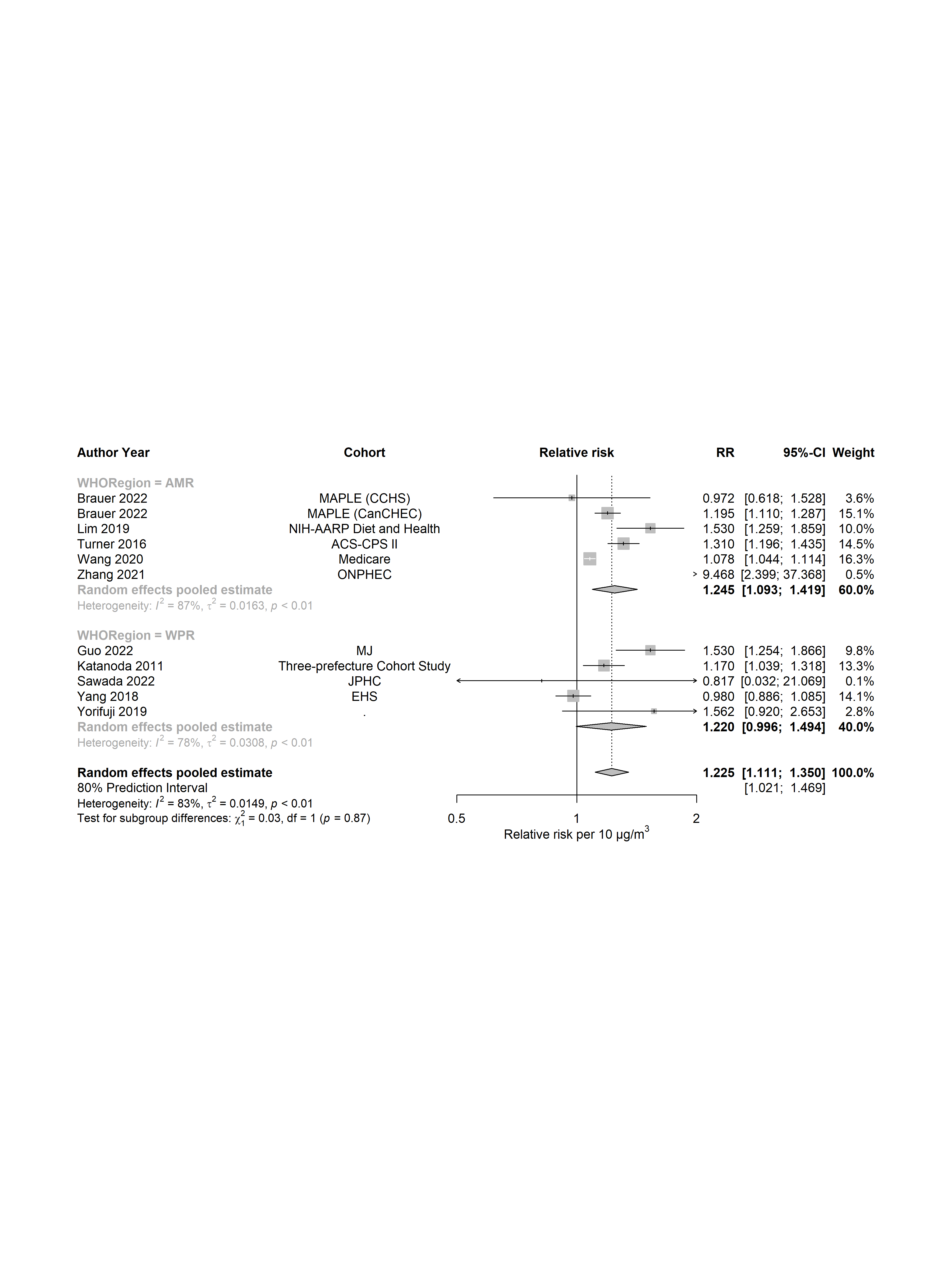


### **FIGURE S36** | Forest plot of the association between PM_2.5_ and acute lower respiratory infection (ALRI) mortality. Subgroup analysis by WHO region: Region of the Americas (AMR), Western Pacific Region (WPR) (Global, 2023-2024).


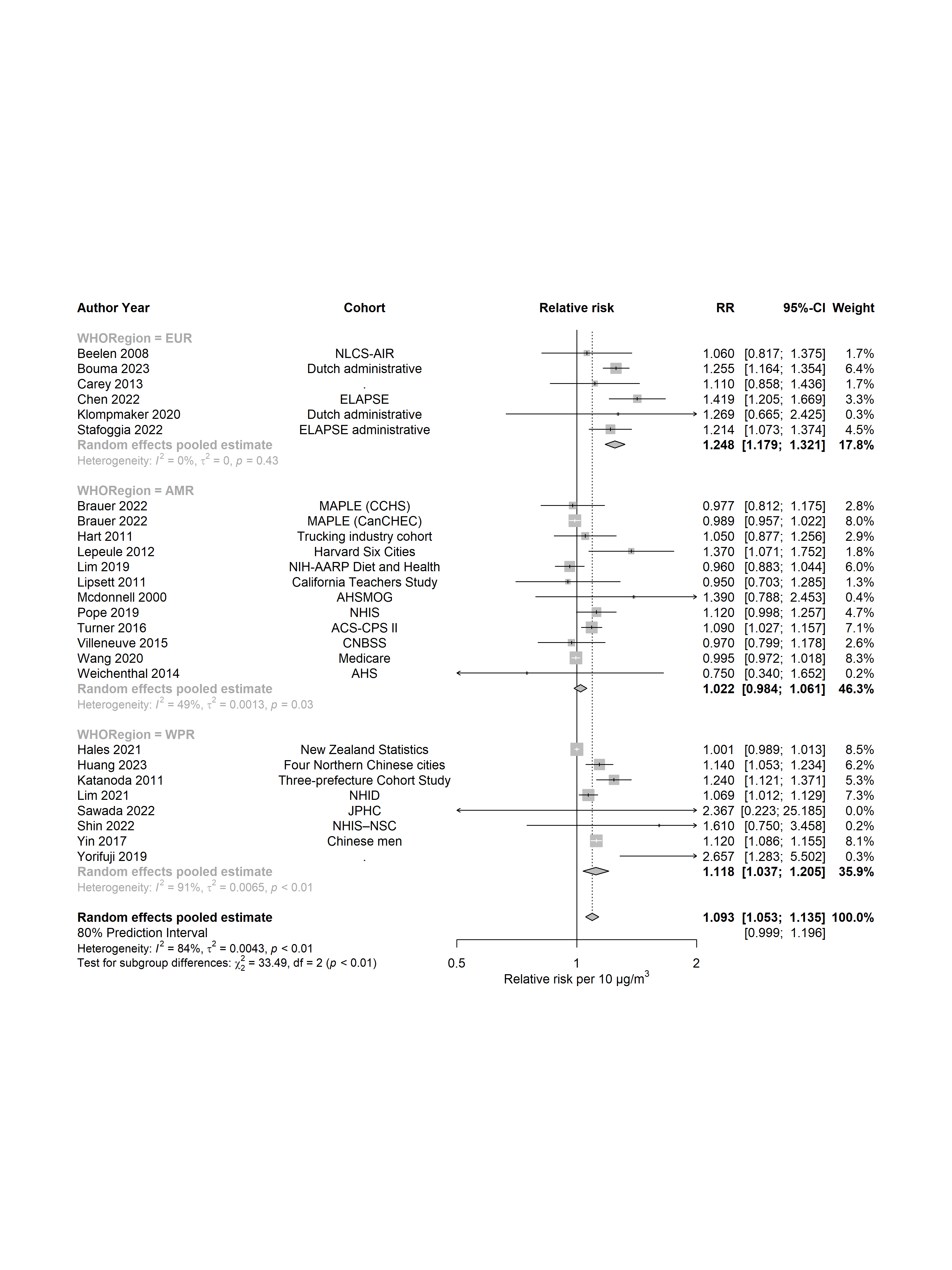


### **FIGURE S37** | Forest plot of the association between PM_2.5_ and lung cancer mortality. Subgroup analysis by WHO region: European Region (EUR), Region of the Americas (AMR), Western Pacific Region (WPR) (Global, 2023-2024).


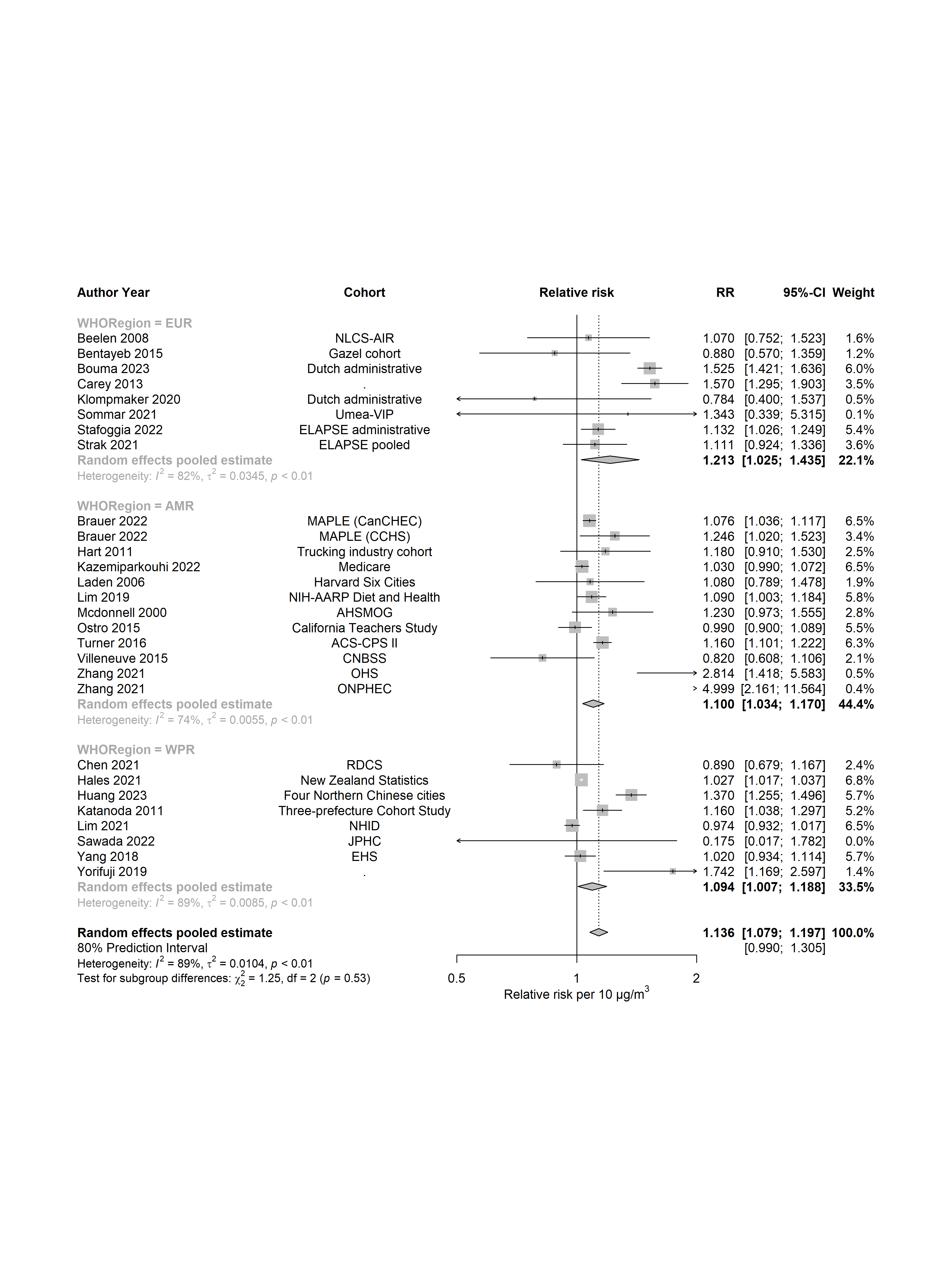


### **FIGURE S38** | Forest plot of the association between PM_2.5_ and respiratory mortality. Subgroup analysis by WHO region: European Region (EUR), Region of the Americas (AMR), Western Pacific Region (WPR) (Global, 2023-2024).


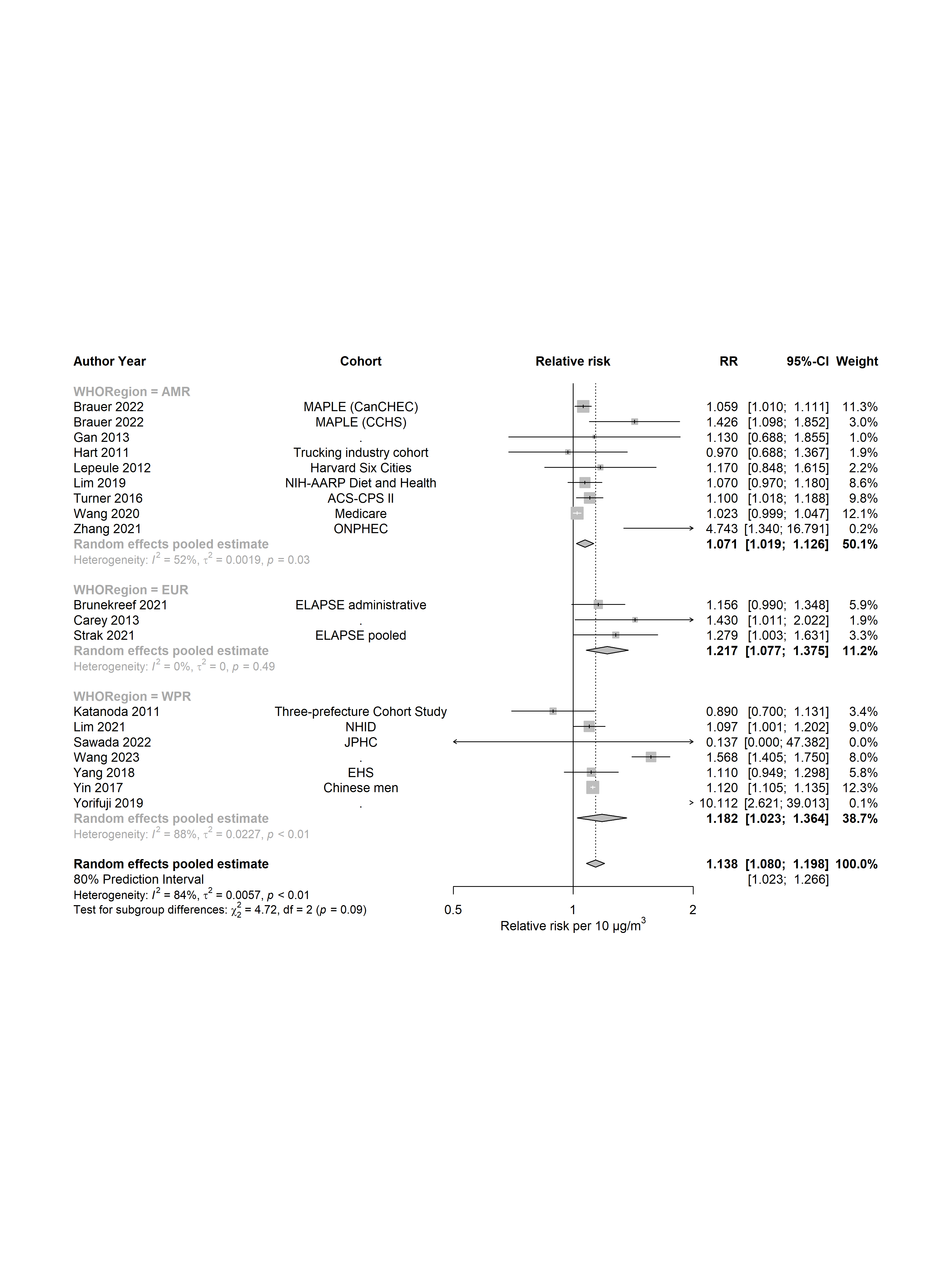


### **FIGURE S39** | Forest plot of the association between PM_2.5_ and chronic obstructive pulmonary disease (COPD) mortality. Subgroup analysis by WHO region: European Region (EUR), Region of the Americas (AMR), Western Pacific Region (WPR) (Global, 2023-2024).


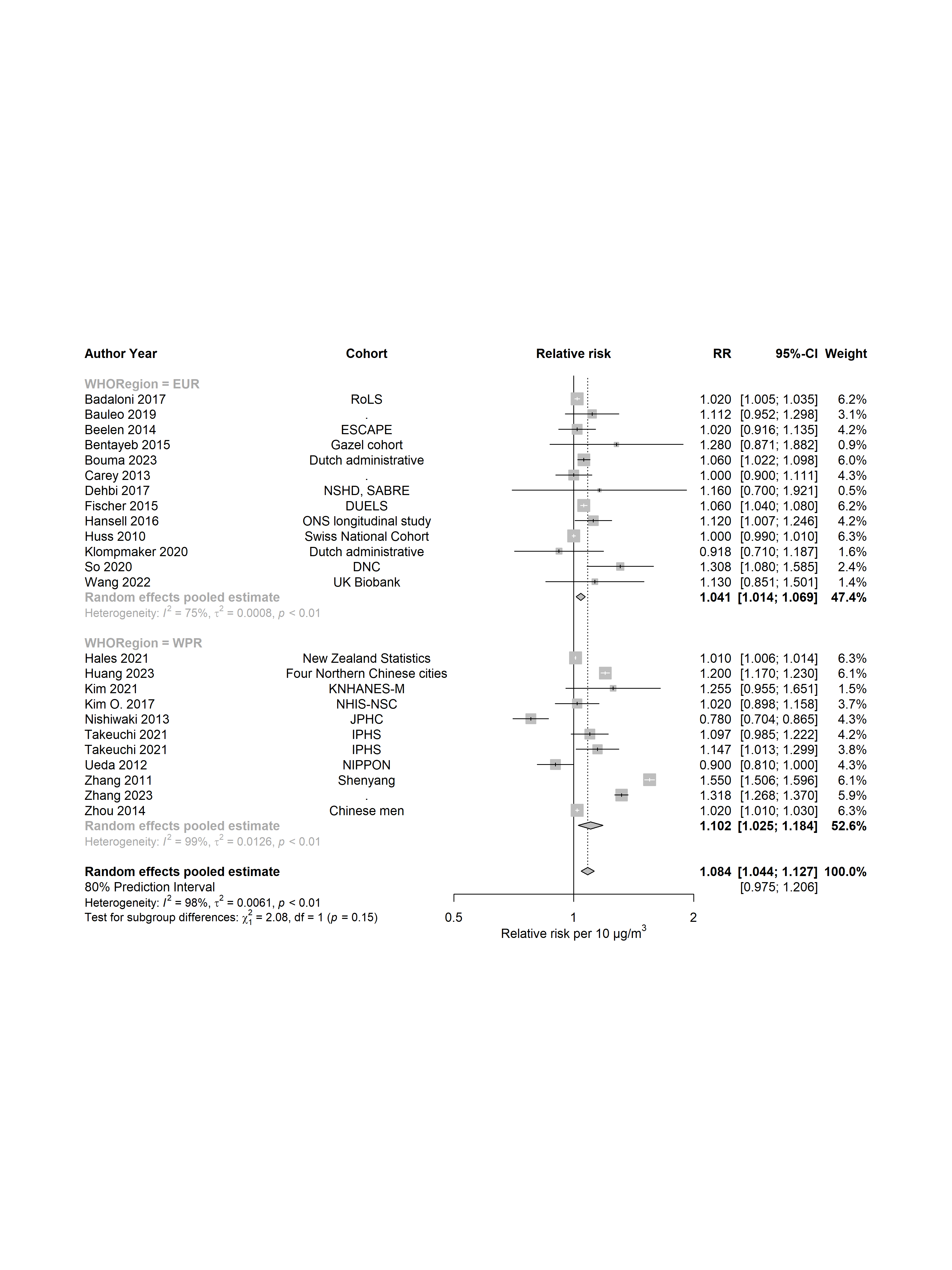


### **FIGURE S40** | Forest plot of the association between PM_10_ and circulatory mortality. Subgroup analysis by WHO region: European Region (EUR), Western Pacific Region (WPR) (Global, 2023-2024).


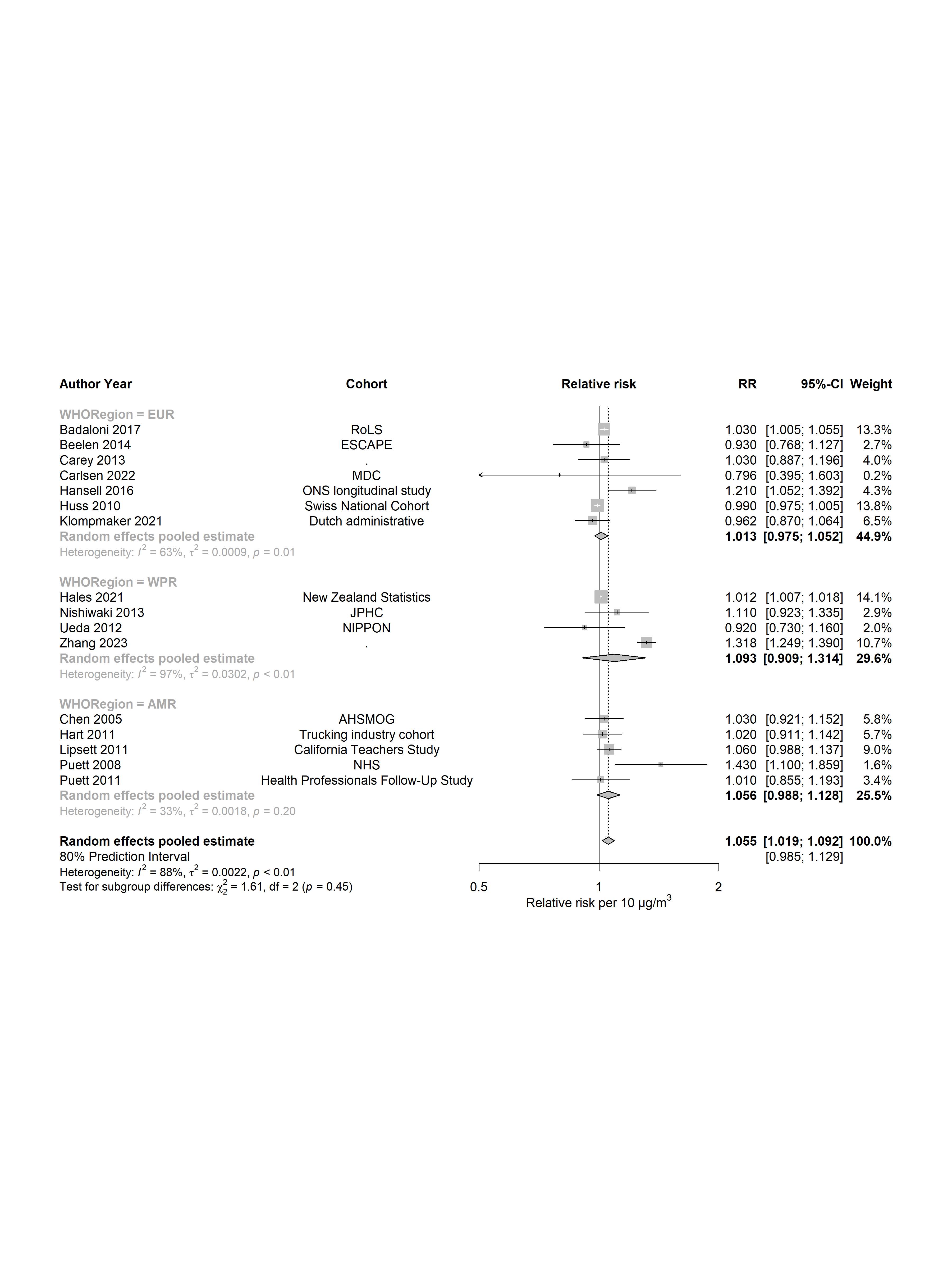


### **FIGURE S41** | Forest plot of the association between PM_10_ and ischaemic heart disease (IHD) mortality. Subgroup analysis by WHO region: European Region (EUR), Region of the Americas (AMR), Western Pacific Region (WPR) (Global, 2023-2024).


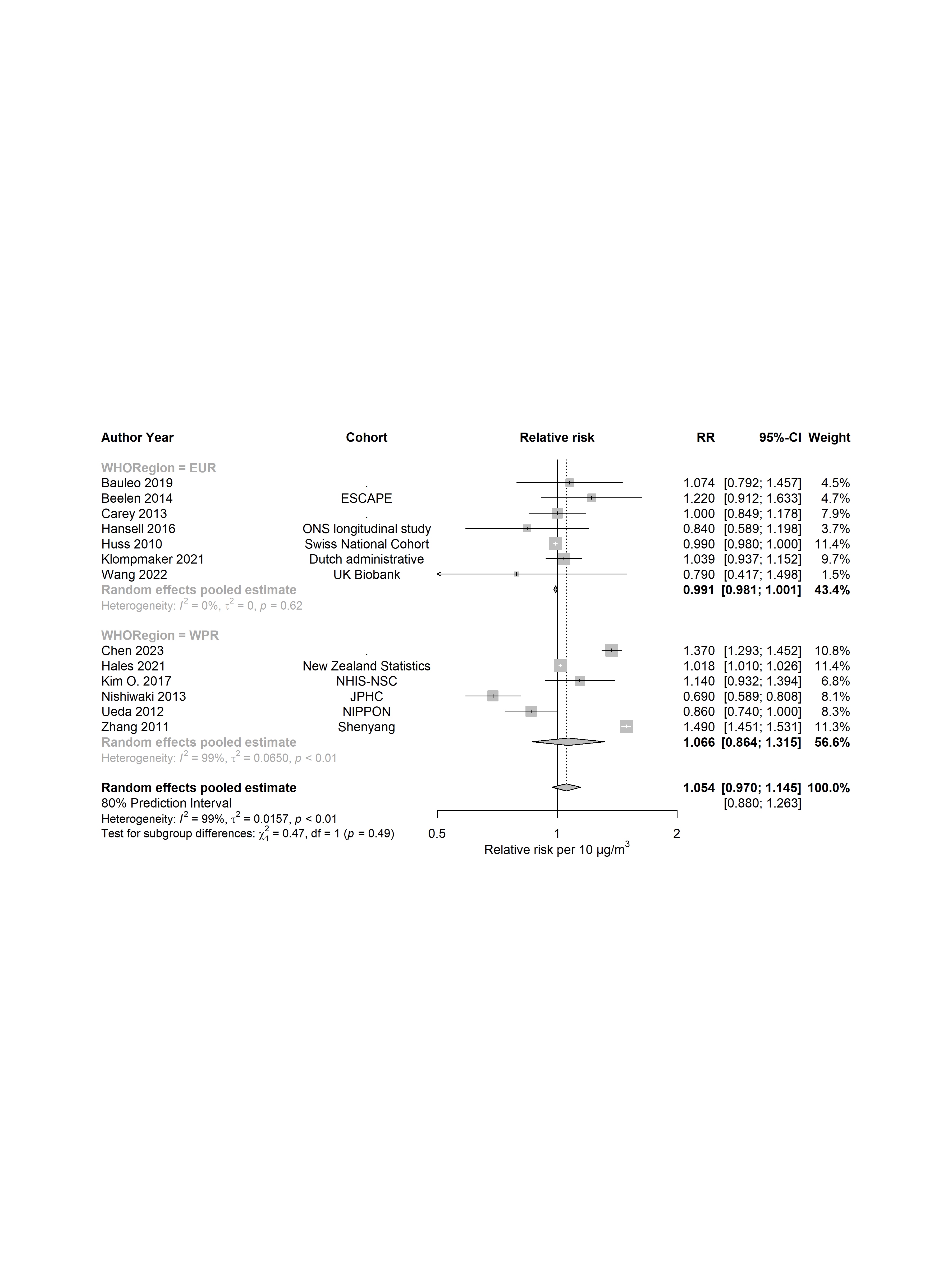


### **FIGURE S42** | Forest plot of the association between PM_10_ and cerebrovascular mortality. Subgroup analysis by WHO region: European Region (EUR), Western Pacific Region (WPR) (Global, 2023-2024).


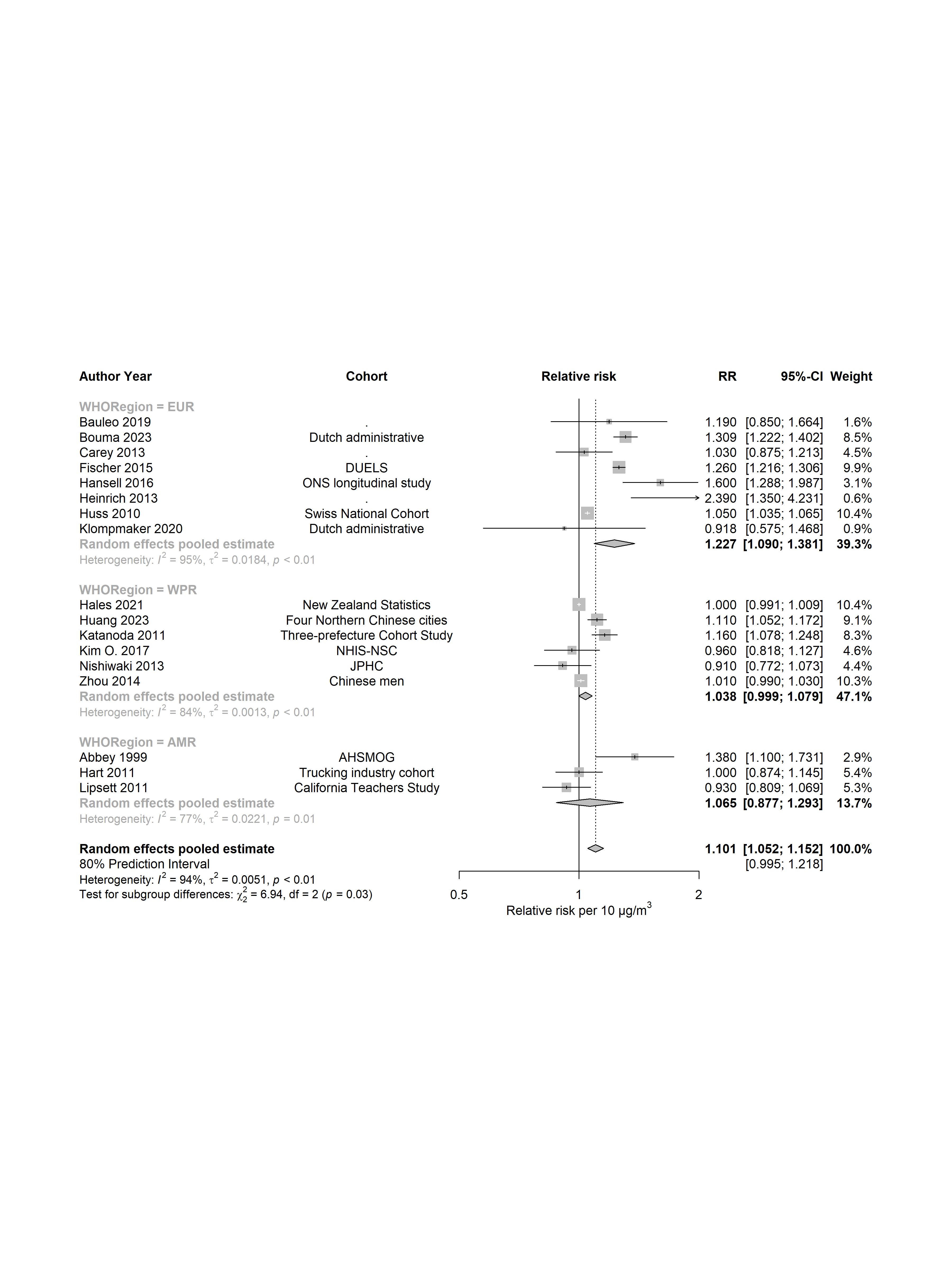


### **FIGURE S43** | Forest plot of the association between PM_10_ and lung cancer mortality. Subgroup analysis by WHO region: European Region (EUR), Region of the Americas (AMR), Western Pacific Region (WPR) (Global, 2023-2024).


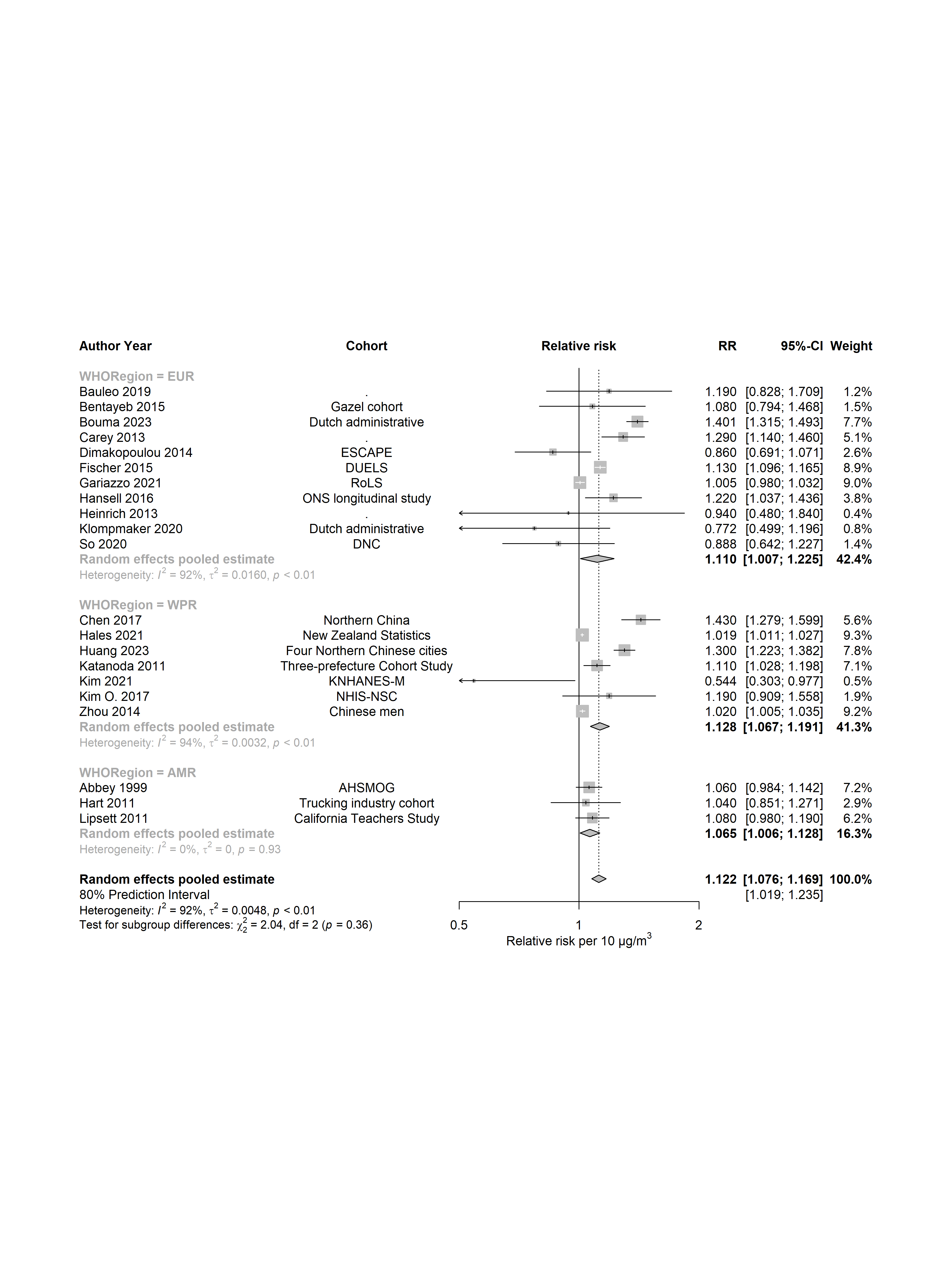


### **FIGURE S44** | Forest plot of the association between PM_10_ and respiratory mortality. Subgroup analysis by WHO region: European Region (EUR), Region of the Americas (AMR), Western Pacific Region (WPR) (Global, 2023-2024).


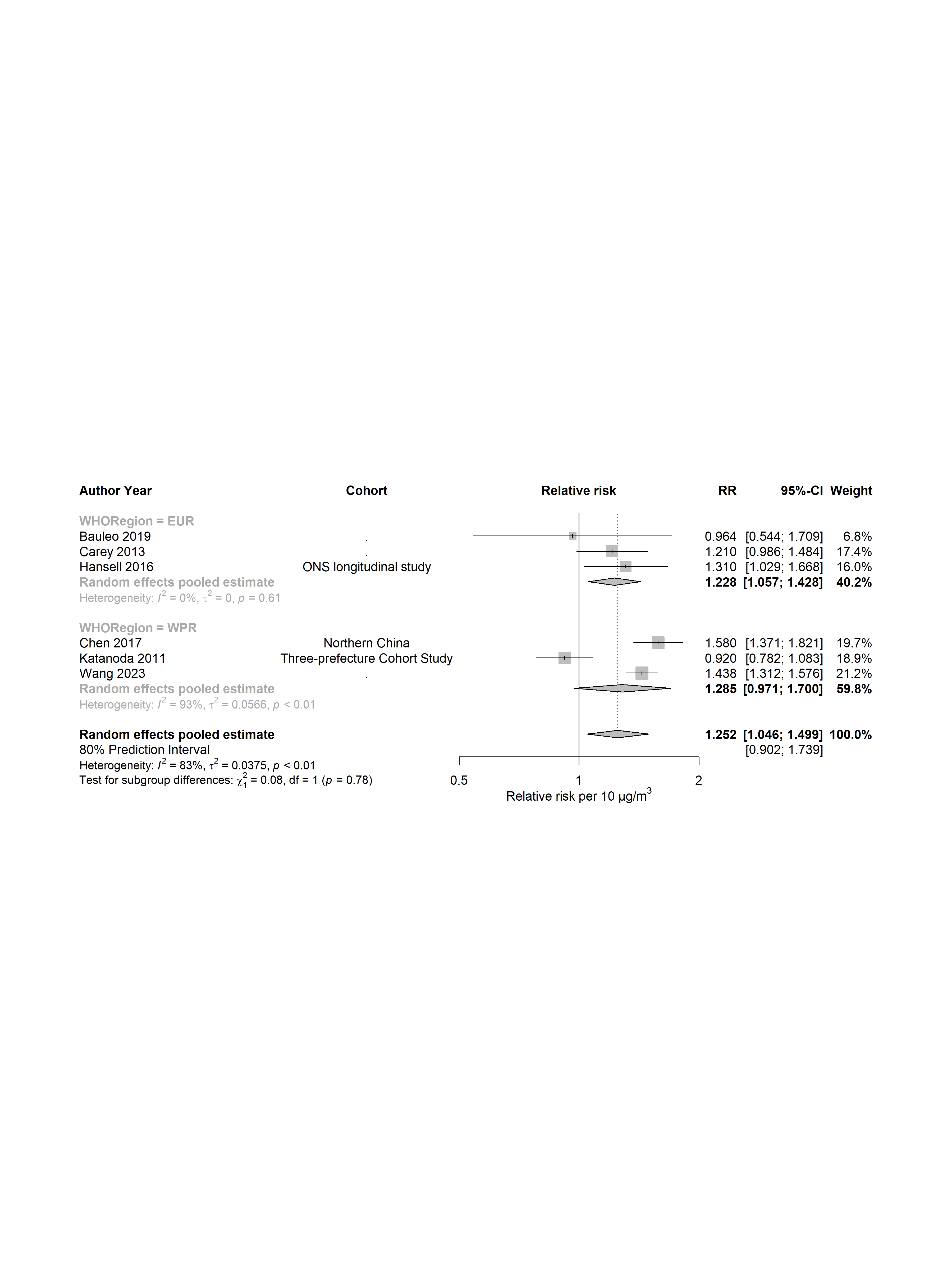


### **FIGURE S45** | Forest plot of the association between PM_10_ and chronic obstructive pulmonary disease (COPD) mortality. Subgroup analysis by WHO region: European Region (EUR), Western Pacific Region (WPR) (Global, 2023-2024).

### **TABLE S17a** | PRISMA checklist (Global, 2023-2024).

| **Section and Topic** | **Item #** | **Checklist item** | **Location where item is reported** |
| --- | --- | --- | --- |
| **TITLE** | | |  |
| Title | 1 | Identify the report as a systematic review. | Title, abstract, methods. |
| **ABSTRACT** | | |  |
| Abstract | 2 | See the PRISMA 2020 for Abstracts checklist. |  |
| **INTRODUCTION** | | |  |
| Rationale | 3 | Describe the rationale for the review in the context of existing knowledge. | Introduction. |
| Objectives | 4 | Provide an explicit statement of the objective(s) or question(s) the review addresses. | Introduction, methodology-review question |
| **METHODS** | | |  |
| Eligibility criteria | 5 | Specify the inclusion and exclusion criteria for the review and how studies were grouped for the syntheses. | Methodology- eligibility criteria and selection procedure |
| Information sources | 6 | Specify all databases, registers, websites, organisations, reference lists and other sources searched or consulted to identify studies. Specify the date when each source was last searched or consulted. | Methodology-search strategy |
| Search strategy | 7 | Present the full search strategies for all databases, registers and websites, including any filters and limits used. | Table S21, online supplemental file 1 |
| Selection process | 8 | Specify the methods used to decide whether a study met the inclusion criteria of the review, including how many reviewers screened each record and each report retrieved, whether they worked independently, and if applicable, details of automation tools used in the process. | Methodology- eligibility criteria and selection procedure |
| Data collection process | 9 | Specify the methods used to collect data from reports, including how many reviewers collected data from each report, whether they worked independently, any processes for obtaining or confirming data from study investigators, and if applicable, details of automation tools used in the process. | Methodology- eligibility criteria and selection procedure |
| Data items | 10a | List and define all outcomes for which data were sought. Specify whether all results that were compatible with each outcome domain in each study were sought (e.g. for all measures, time points, analyses), and if not, the methods used to decide which results to collect. | Methodology- eligibility criteria and selection procedure |
|  | 10b | List and define all other variables for which data were sought (e.g. participant and intervention characteristics, funding sources). Describe any assumptions made about any missing or unclear information. | Methodology- eligibility criteria and selection procedure |
| Study risk of bias assessment | 11 | Specify the methods used to assess risk of bias in the included studies, including details of the tool(s) used, how many reviewers assessed each study and whether they worked independently, and if applicable, details of automation tools used in the process. | Methodology- risk of bias assessment |
| Effect measures | 12 | Specify for each outcome the effect measure(s) (e.g. risk ratio, mean difference) used in the synthesis or presentation of results. | Methodology- data synthesis |
| Synthesis methods | 13a | Describe the processes used to decide which studies were eligible for each synthesis (e.g. tabulating the study intervention characteristics and comparing against the planned groups for each synthesis (item #5)). | Methodology- data synthesis |
|  | 13b | Describe any methods required to prepare the data for presentation or synthesis, such as handling of missing summary statistics, or data conversions. | Methodology- data synthesis |
|  | 13c | Describe any methods used to tabulate or visually display results of individual studies and syntheses. | Methodology- data synthesis |
|  | 13d | Describe any methods used to synthesize results and provide a rationale for the choice(s). If meta-analysis was performed, describe the model(s), method(s) to identify the presence and extent of statistical heterogeneity, and software package(s) used. | Methodology- data synthesis |
|  | 13e | Describe any methods used to explore possible causes of heterogeneity among study results (e.g. subgroup analysis, meta-regression). | Methodology- data synthesis |
|  | 13f | Describe any sensitivity analyses conducted to assess robustness of the synthesized results. | Methodology- data synthesis |
| Reporting bias assessment | 14 | Describe any methods used to assess risk of bias due to missing results in a synthesis (arising from reporting biases). | Methodology- risk of bias assessment |
| Certainty assessment | 15 | Describe any methods used to assess certainty (or confidence) in the body of evidence for an outcome. | Methodology- Certainty of the evidence |
| **RESULTS** | | |  |
| Study selection | 16a | Describe the results of the search and selection process, from the number of records identified in the search to the number of studies included in the review, ideally using a flow diagram. | Results- Description of studies |
|  | 16b | Cite studies that might appear to meet the inclusion criteria, but which were excluded, and explain why they were excluded. | Online supplemental file 2 |
| Study characteristics | 17 | Cite each included study and present its characteristics. | Online supplemental file 4 |
| Risk of bias in studies | 18 | Present assessments of risk of bias for each included study. | Online supplemental file 5 |
| Results of individual studies | 19 | For all outcomes, present, for each study: (a) summary statistics for each group (where appropriate) and (b) an effect estimate and its precision (e.g. confidence/credible interval), ideally using structured tables or plots. | Online supplemental file 4 and 6 |
| Results of syntheses | 20a | For each synthesis, briefly summarise the characteristics and risk of bias among contributing studies. | Results- risk of bias |
|  | 20b | Present results of all statistical syntheses conducted. If meta-analysis was done, present for each the summary estimate and its precision (e.g. confidence/credible interval) and measures of statistical heterogeneity. If comparing groups, describe the direction of the effect. | Results- meta-analysis |
|  | 20c | Present results of all investigations of possible causes of heterogeneity among study results. | Results- meta-analysis (subgroup analyses) |
|  | 20d | Present results of all sensitivity analyses conducted to assess the robustness of the synthesized results. | Results- meta-analysis (sensitivity analysis) |
| Reporting biases | 21 | Present assessments of risk of bias due to missing results (arising from reporting biases) for each synthesis assessed. | Results- meta-analysis (publication bias) |
| Certainty of evidence | 22 | Present assessments of certainty (or confidence) in the body of evidence for each outcome assessed. | Results- certainty of the evidence |
| **DISCUSSION** | | |  |
| Discussion | 23a | Provide a general interpretation of the results in the context of other evidence. | Discussion (first part) |
|  | 23b | Discuss any limitations of the evidence included in the review. | Discussion |
|  | 23c | Discuss any limitations of the review processes used. | Discussion |
|  | 23d | Discuss implications of the results for practice, policy, and future research. | Discussion (last part) |
| **OTHER INFORMATION** | | |  |
| Registration and protocol | 24a | Provide registration information for the review, including register name and registration number, or state that the review was not registered. | Methods- protocol and reporting |
|  | 24b | Indicate where the review protocol can be accessed, or state that a protocol was not prepared. | Methods- protocol and reporting |
|  | 24c | Describe and explain any amendments to information provided at registration or in the protocol. | Methods- deviations from the protocol |
| Support | 25 | Describe sources of financial or non-financial support for the review, and the role of the funders or sponsors in the review. | Funding |
| Competing interests | 26 | Declare any competing interests of review authors. | Competing interests |
| Availability of data, code and other materials | 27 | Report which of the following are publicly available and where they can be found: template data collection forms; data extracted from included studies; data used for all analyses; analytic code; any other materials used in the review. | All the databases needed to replicate the analyses are available online as supplemental files of the article |

### **TABLE S17b** | PRISMA checklist for abstracts (Global, 2023-2024).

| **Section and Topic** | **Item #** | **Checklist item** | **Reported (Yes/No)** |
| --- | --- | --- | --- |
| **TITLE** | | |  |
| Title | 1 | Identify the report as a systematic review. | Yes |
| **BACKGROUND** | | |  |
| Objectives | 2 | Provide an explicit statement of the main objective(s) or question(s) the review addresses. | Yes |
| **METHODS** | | |  |
| Eligibility criteria | 3 | Specify the inclusion and exclusion criteria for the review. | No |
| Information sources | 4 | Specify the information sources (e.g. databases, registers) used to identify studies and the date when each was last searched. | Yes |
| Risk of bias | 5 | Specify the methods used to assess risk of bias in the included studies. | No |
| Synthesis of results | 6 | Specify the methods used to present and synthesise results. | Yes |
| **RESULTS** | | |  |
| Included studies | 7 | Give the total number of included studies and participants and summarise relevant characteristics of studies. | Yes/No |
| Synthesis of results | 8 | Present results for main outcomes, preferably indicating the number of included studies and participants for each. If meta-analysis was done, report the summary estimate and confidence/credible interval. If comparing groups, indicate the direction of the effect (i.e. which group is favoured). | Yes |
| **DISCUSSION** | | |  |
| Limitations of evidence | 9 | Provide a brief summary of the limitations of the evidence included in the review (e.g. study risk of bias, inconsistency and imprecision). | Yes |
| Interpretation | 10 | Provide a general interpretation of the results and important implications. | Yes |
| **OTHER** | | |  |
| Funding | 11 | Specify the primary source of funding for the review. | No |
| Registration | 12 | Provide the register name and registration number. | Yes |

### **TABLE S18** | Search strategy (Global, 2023-2024).

| **PubMed (01/09/2018 to 22/05/2024)** |
| --- |
| (mortality[MH] OR death*[MH] or mortality[TIAB] OR death*[TIAB]) AND (cohort*[TIAB] OR cox[TIAB] OR hazard*[TIAB]) AND (air pollution[MH] OR PM10[TIAB] OR PM2[TIAB] OR particle*[TIAB] OR particulate*[TIAB] OR ozone[TIAB] OR O3[TIAB] OR NO2[TIAB] OR nitrogen dioxide[TIAB]) |
| **Embase (2018 to 22/05/2024)** |
| ((mortality or death).sh or (mortality or death).tw) and (air pollution.sh or (particle* or particulate* or PM10 or PM2* or nitrogen dioxide or NO2 or ozone or O3).tw) and (cohort* or cox or hazard).tw |

### **TABLE S19** | Causes of death included in the review, by category and subcategory (Global, 2023-2024).

| **Disease category by protocol** | **Coding in our databases** | **Disease subcategory**  **(included in the disease category)** |
| --- | --- | --- |
| All-causes  (A00 – Z99) | ACM | Non accidental causes  (A00-R99) |
| Circulatory diseases  (I00 – I99) | CIRC | Cardiovascular diseases  (I10-I70) |
| Ischaemic heart diseases (IHD)  (I20 – I25) | IHD | Acute myocardial infarction (I21-I23)  CHD  (I20 - I25) |
| Cerebrovascular diseases  (I60 – I69) | CeVD | Stroke  (I64) |
| Acute lower respiratory infection (ALRI)  (J12 – J18, J20 – J22) | ALRI | Pneumonia  (J12-J18) |
| lung cancer  (C33-C34: malignant neoplasm of the trachea, bronchus or lung only) | LC |  |
| Respiratory disease  (J00 - J99) | RESP |  |
| COPD  (J40 - J44, J47) | COPD |  |
| Post-neonatal mortality  (ages 1 - 12 months) | PNN |  |

### **TABLE S20** | Pooled effect sizes for PM_2.5_ and all-cause and cause-specific mortality. Subgroup analysis by study size (Global, 2023-2024).

| **Outcome (mortality)** | **Study size** | **N** | **RR (95% CI)** | **I^2^ (%)** | **p-value** |
| --- | --- | --- | --- | --- | --- |
| All-cause | Larger | 9 | 1.086 (1.013-1.160) | 99.9 | 0.77 |
|  | Smaller | 44 | 1.096 (1.071-1.120) | 96.4 |  |
| Circulatory | Larger | 7 | 1.084 (1.040-1.129) | 98.0 | 0.03 |
|  | Smaller | 35 | 1.146 (1.112-1.181) | 93.1 |  |
| IHD | Larger | 8 | 1.129 (1.063-1.199) | 98.3 | 0.59 |
|  | Smaller | 26 | 1.153 (1.102-1.206) | 74.5 |  |
| Cerebrovascular | Larger | 6 | 1.067 (1.027-1.109) | 80.1 | <0.01 |
|  | Smaller | 22 | 1.193 (1.129-1.261) | 85.0 |  |
| ALRI | Larger | N/A | N/A | N/A | N/A |
|  | Smaller | 10 | 1.247 (1.070-1.453) | 79.8 |  |
| Lung cancer | Larger | 6 | 1.055 (1.010-1.102) | 89.8 | 0.10 |
|  | Smaller | 20 | 1.116 (1.061-1.174) | 55.4 |  |
| Respiratory | Larger | 6 | 1.108 (1.025-1.197) | 96.3 | 0.44 |
|  | Smaller | 22 | 1.157 (1.072-1.249) | 74.1 |  |
| COPD | Larger | 4 | 1.051 (1.012-1.092) | 43.9 | 0.01 |
|  | Smaller | 15 | 1.192 (1.091-1.302) | 77.8 |  |

RR, pooled relative risks; 95% CI, 95% confidence interval; I^2^, test for heterogeneity; p-value, significance of test for difference between subgroups (interaction); IHD, ischaemic heart disease; ALRI, acute lower respiratory infection; COPD, chronic obstructive pulmonary disease; Larger, studies including more than 1,000,000 participants; smaller, studies including less than 1,000,000 participants; N/A, not applicable.

### **TABLE S21** | Pooled effect sizes for PM_10_ and all-cause and cause-specific mortality. Subgroup analysis by study size (Global, 2023-2024).

| **Outcome (mortality)** | **Study size** | **N** | **RR (95% CI)** | **I^2^ (%)** | **p-value** |
| --- | --- | --- | --- | --- | --- |
| All-cause | Larger | 5 | 1.059 (1.022-1.097) | 98.7 | 0.35 |
|  | Smaller | 23 | 1.090 (1.022-1.097) | 97.3 |  |
| Circulatory | Larger | 5 | 1.025 (1.008-1.042) | 89.2 | 0.12 |
|  | Smaller | 21 | 1.097 (1.009-1.193) | 97.9 |  |
| IHD | Larger | 4 | 1.008 (0.990-1.025) | 71.8 | 0.10 |
|  | Smaller | 12 | 1.084 (0.994-1.182) | 80.1 |  |
| Cerebrovascular | Larger | 3 | 1.006 (0.980-1.032) | 89.2 | 0.66 |
|  | Smaller | 12 | 1.040 (0.897-1.207) | 95.4 |  |
| ALRI | Larger | 0 | N/A | N/A | N/A |
|  | Smaller | 1 | N/A | N/A |  |
| Lung cancer | Larger | 4 | 1.140 (1.051-1.236) | 98.6 | 0.35 |
|  | Smaller | 13 | 1.084 (1.011-1.161) | 79.3 |  |
| Respiratory | Larger | 3 | 1.168 (1.020-1.338) | 98.5 | 0.46 |
|  | Smaller | 18 | 1.106 (1.045-1.170) | 86.5 |  |
| COPD | Larger | 0 | N/A | N/A | N/A |
|  | Smaller | 7 | 1.215 (1.027-1.438) | 82.9 |  |

RR, pooled relative risks; 95% CI, 95% confidence interval; I^2^, test for heterogeneity; p-value, significance of test for difference between subgroups (interaction); IHD, ischaemic heart disease; ALRI, acute lower respiratory infection; COPD, chronic obstructive pulmonary disease; Larger, studies including more than 1,000,000 participants; smaller, studies including less than 1,000,000 participants; N/A, not applicable.

### **TABLE S22** | Description of the certainty of evidence tool and criteria (Global, 2023-2024).

| *General description* | The tool consists of five domains that can be used to downgrade the certainty of the evidence: limitation in studies, indirectness, inconsistency, imprecision and publication bias. Three other domains are then assessed, but these are used to update the certainty of the evidence: large magnitude of effect, occurrence of all possible confounders shifting towards the null effect, and evidence of a concentration-response gradient (of function). The procedure is as follows 1) select a given exposure-outcome pair, 2) assign a moderate level of certainty, 3) apply the first five domains and decide whether to downgrade the certainty of the evidence according to these criteria, 4) apply the other three domains and decide whether to upgrade the certainty of the evidence, and 5) summarise the evidence. The quality of the evidence starts at 3 points, and each downgrading or upgrading of the evidence means the addition or subtraction of one point. The final rating of the certainty of the evidence could have four values: 1) high, meaning that further research is very unlikely to change the confidence in the estimate of the effect; 2) moderate, meaning that further research is likely to have an important effect on the confidence in the estimate of the effect; 3) low, meaning that further research is very likely to have an important effect on the confidence in the estimate of the effect; and 4) very low, meaning that the estimate of the effect is very uncertain. Below we provide more details on the criteria used for each domain. |
| --- | --- |
| *Limitations in studies* | We downgraded the evidence after analysing the results of the sensitivity assessment. The evidence was downgraded if there were differences in the direction or significance of the effect estimates before and after excluding studies at high risk of bias. |
| *Indirectness* | This domain analyses the difference between the research question in the original studies and in the systematic review. We did not consider this domain relevant because the PECOS question was well defined and consistent with the research questions in the individual studies. |
| *Inconsistency* | This domain analyses the presence of inconsistent results due to heterogeneity around pooled effect estimates. The domain is complex, because considerable heterogeneity is expected in observational studies. Our first approach was to observe the 80% prediction interval. However, we also considered the consistency in the direction of the associations, because regardless of the variability, if a significant proportion of studies are consistent in the direction of the associations, the association itself can be considered consistent. This consistency assessment included the proportion of studies with significant positive or negative relative risks. We also considered whether the variation in the estimates could be explained by means of the subgroup analysis. Given the complexity of the criteria, the assessment had to be made on a case-by-case basis. |
| *Imprecision* | If effect estimates were calculated with less than 940,000 person-years for the sum of all included studies for a given exposure-outcome association, the evidence was reduced by one level. This cut-off value was calculated for the previous review and in general for the reviews that informed the 2021 WHO global air quality guidelines. |
| *Publication bias* | Because tests to measure asymmetry in the funnel plots are affected by heterogeneity in observational studies of air pollution and cannot be considered reliable, we also examined the difference in relative risks estimated from smaller and larger cohort studies, considering larger a study with more than 1 million participants. For a given exposure-outcome pair to be considered affected by publication bias, three conditions should be met: 1) a significant Egger's test; 2) a visually detectable asymmetry of the funnel plot; and 3) a statistical difference in the effect estimates between smaller and larger studies. |
| *Large effect size* | Consistent with the previous review, we judged that the information on the relationship between air pollution exposures and confounders was not sufficient to make robust assumptions, and in addition, the relative risks between exposures and mortality would typically be lower than the effect estimates between common confounders and mortality, as in the case of smoking. Accordingly, we did not upgrade the evidence based on this domain. |
| *Confounding domain* | We considered that we had no theoretical basis to state that the potential confounders should shift the relative risks in only one direction, then the evidence was not upgraded based on this domain. |
| *Concentration- response gradient* | Regardless of the shape of the concentration-response function, if the meta-analysis found a positive and significant association for a given exposure-outcome pair, the evidence was upgraded by one level. |

### **TABLE S23** | Overall results of the risk of bias assessment by domain (Global, 2023-2024).

| *Confounding domain* | A total of 15 studies were considered to have a high risk of bias because fewer than all critical confounders were included in the models, such as BMI or smoking. Many other studies were considered to have a moderate risk of bias because they did not adjust for one or more of the additional confounders. We rated a number of studies as moderate because they used an indirect method to adjust for BMI or smoking. |
| --- | --- |
| *Selection bias* | In the only study rated as 'high risk of bias', the rationale was related to the inclusion of different populations in terms of age and socioeconomic status exposed to different ambient pollutant concentrations. |
| *Exposure assessment* | Five studies were considered to be at high risk of bias due to the time period used to measure exposure. In these studies, information on PM levels was only collected at baseline or calculated as an average over the period, which could lead to bias if this exposure varied over time. Many studies were considered to have a low risk of bias because a time-varying analysis was performed. |
| *Outcome assessment* | Only three studies were flagged as being at high risk of bias. One of these studies did not report any coding of mortality information, e.g. International Classification of Diseases (ICD). However, this assessment was only applied to specific-cause mortality, as it was assumed that the information on all-cause mortality was not affected by this lack of coding. In the other two studies, data on health outcomes were collected retrospectively from family members in the surveys. |
| *Missing data* | With regard to outcome bias, missing information is generally unlikely because almost all studies used official registries to collect data. For exposure, some studies reported a small proportion of missing data, while others used imputation methods to overcome this problem. In any case, bias due to missing data was considered unlikely. Therefore, no studies were considered to be at high risk of bias, and only to studies were considered to be at moderate risk of bias. |
| *Selective reporting* | All studies included in this update were considered to have a low risk of bias. The only exception was one study included in the previous review, which selectively reported associations in men because the same effects were weak or reversed in women. |

### **TABLE S24** | Results of the certainty of evidence assessment by domain (Global, 2023-2024).

| *Limitations in studies* | None of the exposure-outcome pairs showed different values for the direction and significance of the associations before and after excluding studies with a high risk of bias. The only exception was the association between PM_10_ and COPD mortality, which showed non-significant results for confounding but not for selection bias. As the direction of the association remained the same, we decided not to downgrade the evidence. |
| --- | --- |
| *Indirectness* | As all studies in this systematic review addressed the research question, this domain was not considered for downgrading. |
| *Inconsistency* | For PM_2.5_ and PM_10_ and the association with all-cause mortality, the prediction interval included unity and was twice the 95% confidence interval, but heterogeneity was not considered to affect the association because almost all individual values in the studies were positive and significant. For cause-specific mortality, some of the exposure-outcome pairs had wide prediction intervals including unity, but the heterogeneity was discarded because consistent positive effect estimates were reported across studies, or because the source of the heterogeneity was explained by differences in pollutant levels or differences among WHO regions. |
| *Imprecision* | For all exposure-outcome pairs, associations were estimated with a number of person-years well above 940,000. |
| *Publication bias* | Six exposure-outcome pairs showed evidence of funnel plot asymmetry. However, the evidence was not downgraded in this domain if the comparison of pooled estimates between smaller and larger studies was not significant. These comparisons can be seen in the online Supplemental Material, tables S24 and S25. In these tables, it can be seen that among the pairs showing funnel plot asymmetry, the PM2.5 and circulatory or cerebrovascular mortality pairs showed statistical differences between smaller and larger studies, which determined the downgrading of the evidence. |
| *Large effect size* | We did not upgrade for this domain, because the relative risks obtained from air pollution studies are typically low compared to interventional studies. |
| *All plausible confounding biases RR to zero* | We did not upgrade for this domain because several potential confounders would shift the relative risk in either direction. |
| *Concentration-response gradient* | In general, we have upgraded the evidence in this domain, as the associations were positive and significant in all but one pair, with the addition of a number of studies reporting different shapes for this concentration-response function. The exception was PM_10_ and cerebrovascular mortality, where we estimated a non-significant association and then did not upgrade the certainty of the evidence. |
